# The Aurora kinase/β-catenin axis contributes to dexamethasone resistance in leukemia

**DOI:** 10.1038/s41698-021-00148-5

**Published:** 2021-02-17

**Authors:** Kinjal Shah, Mehreen Ahmed, Julhash U. Kazi

**Affiliations:** 1 Division of Translational Cancer Research, Department of Laboratory Medicine, Lund University, Lund, Sweden; 2 Lund Stem Cell Center, Department of Laboratory Medicine, Lund University, Lund, Sweden

**Keywords:** Acute lymphocytic leukaemia, Cancer therapeutic resistance

## Abstract

Glucocorticoids, such as dexamethasone and prednisolone, are widely used in cancer treatment. Different hematological malignancies respond differently to this treatment which, as could be expected, correlates with treatment outcome. In this study, we have used a glucocorticoid-induced gene signature to develop a deep learning model that can predict dexamethasone sensitivity. By combining gene expression data from cell lines and patients with acute lymphoblastic leukemia, we observed that the model is useful for the classification of patients. Predicted samples have been used to detect deregulated pathways that lead to dexamethasone resistance. Gene set enrichment analysis, peptide substrate-based kinase profiling assay, and western blot analysis identified Aurora kinase, S6K, p38, and β-catenin as key signaling proteins involved in dexamethasone resistance. Deep learning-enabled drug synergy prediction followed by in vitro drug synergy analysis identified kinase inhibitors against Aurora kinase, JAK, S6K, and mTOR that displayed synergy with dexamethasone. Combining pathway enrichment, kinase regulation, and kinase inhibition data, we propose that Aurora kinase or its several direct or indirect downstream kinase effectors such as mTOR, S6K, p38, and JAK may be involved in β-catenin stabilization through phosphorylation-dependent inactivation of GSK-3β. Collectively, our data suggest that activation of the Aurora kinase/β-catenin axis during dexamethasone treatment may contribute to cell survival signaling which is possibly maintained in patients who are resistant to dexamethasone.

## Introduction

Acute lymphoblastic leukemia (ALL) is the most common pediatric malignancy of lymphoid progenitor cells, with ~80% of cases occurring in children and the remainder in adults^1^. Glucocorticoids such as dexamethasone and prednisolone are important drugs in the chemotherapeutic regimen for the treatment of ALL^1^. Apart from their strong anti-inflammatory and immune-suppressive actions, glucocorticoids induce growth arrest and apoptosis in ALL. Their action is exerted by binding to the glucocorticoid receptor (GR), a member of the nuclear hormone receptor superfamily that also acts as a transcription factor^2^. Sensitivity to glucocorticoids serves as a positive prognostic indicator, and patients unresponsive to glucocorticoids often relapse, which leads to poor prognosis. Multiple mechanisms of glucocorticoid resistance have been identified such as alterations in expression and function of GR or of GR-associated proteins like chaperones and co-chaperones, thereby affecting their function, and causing defects in the target genes, leading to the inhibition of apoptosis and defective metabolism, cross-talk with other cell signaling pathways and transcription factors, as well as changes in chromatin accessibility^2–5^.

The current chemotherapeutic regimen used for the treatment of ALL, which consists of corticosteroids in conjunction with chemotherapeutic drugs, has resulted in the long-term survival of 80–90% in children and of 40% in adults, though the complete remission (CR) rates are similar in both groups^6^. Despite these positive statistics, the highest cancer-related mortality in children is associated with ALL due to relapse or treatment-related toxic effects. This shows a great need for improving the existing treatments by not only identifying new molecular targets to develop better and less toxic therapeutic agents but also by identifying patients who require less intensive therapy^7^.

The heterogeneity in the patient’s responses to different treatments has resulted in great advancements in the bioinformatics area, where different machine learning approaches are used to better match patients to drugs. Several studies have compared machine learning approaches for monotherapy prediction. The ridge regression model built using baseline gene expression data from cell lines was shown to outperform several machine learning algorithms including random forests, nearest shrunken centroids, principal component regression, lasso, and elastic net to predict in vivo chemotherapy response^8^. A landmark study compared 44 drug sensitivity prediction algorithms in which genomic, epigenomic, and proteomic profiling data for human breast cancer cell lines were used^9^. In that study, models developed using Bayesian multitask multiple kernel learning and random forests were found to perform better than other algorithms. Another large-scale study compared multiple machine learning algorithms using multi-omics data and reported elastic net and ridge regression as the top-performing algorithms^10^. These studies used several thousand features for a limited number of samples to develop the model; therefore, the selection of features might have affected the performance of the algorithm. Besides those low complexity models, deep learning models have been implicated in monotherapy prediction^11^. Because deep learning models are highly sensitive to the feature-to-sample ratio, the feature selection approach was found to be an effective method in which only features highly conserved with the phenotype were used to build the model^12–14^. Such methods are also useful in identifying a potential molecular marker and driver of sensitivity to the drugs^12^.

In this study, we employed transcriptomics, peptide substrate-based kinase profiling, deep learning models, and drug synergy studies to identify the deregulated signaling pathways in dexamethasone-resistant ALL. We show that the short-term dexamethasone treatment induces the Aurora kinase/β-catenin signaling axis which is also found to be enriched in ALL patients that are predicted to be dexamethasone resistant.

## Results

### Glucocorticoids induce a distinct gene signature in ALL

**Fig. 1:**
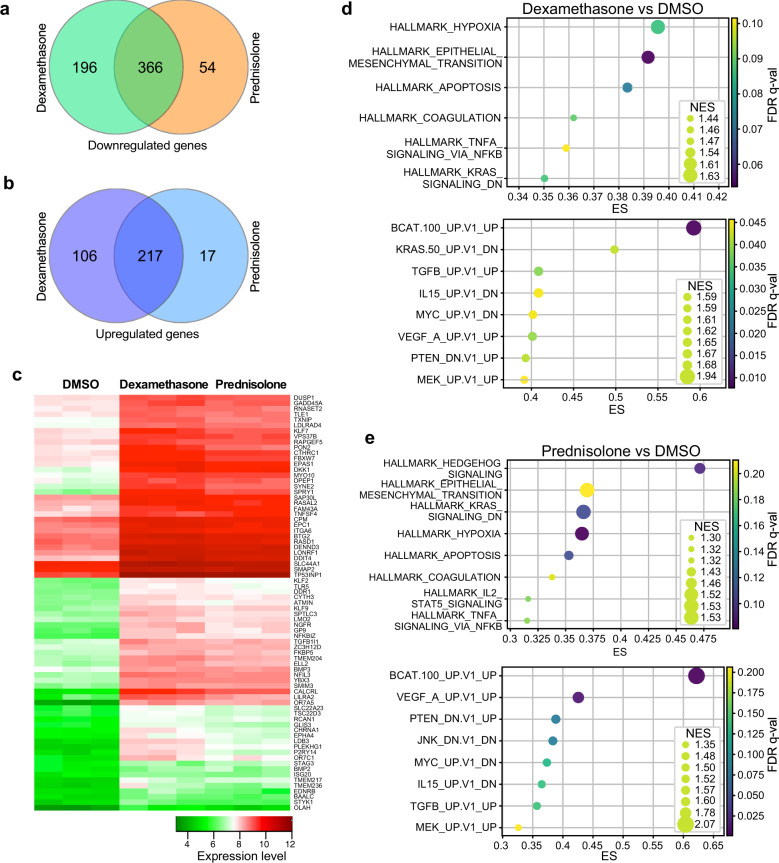
Glucocorticoid-induced regulation in gene expression. SUP-B15 cells were treated with 1 µM dexamethasone, 2 µM prednisolone, or DMSO for 6 h. Global gene expression was measured by Affymetrix Human Gene 2.0 ST Array. **a** The number of downregulated genes in dexamethasone- and prednisolone-treated cells. **b** The number of upregulated genes in dexamethasone and prednisolone-treated cells. **c** The heatmap displaying clusters of upregulated genes in dexamethasone- and prednisolone-treated cells was generated by the heatmap.2 function of Gplots library in R. **d** Gene set enrichments in dexamethasone-treated cells were measured by GSEA (Hallmarks and Oncogenic signatures) and visualized by the pyplot.scatter function of Matplotlib. **e** Gene set enrichments in prednisolone-treated cells were analyzed and visualized as described above.

Long-term treatment with glucocorticoids induces resistance that is mediated by several mechanisms. Even with the drug that can induce a complete response, some cells always survive the treatment. This surviving proportion of cells is referred to as drug-tolerant cells, which eventually provoke drug resistance. Drug-tolerant cells display dynamic fluctuations in gene expression, where survival is thought to be mediated by a group of genes that are upregulated during the drug treatment^15^. To understand how genes are regulated in ALL during glucocorticoid treatment, we used the SUP-B15 cell line as a model system. SUP-B15 cells are highly sensitive to glucocorticoids such as dexamethasone and prednisolone (Supplementary Fig. 1a). We observed that these drugs at micromolar concentrations inhibited more than 90% of cell viability after 48 h of incubation (Supplementary Fig. 1b) and induced a significant level of apoptosis after 24 h of incubation (Supplementary Fig. 1c). However, apoptosis remained almost equal to the DMSO control after treatment of the drugs for 4 h or 6 h (Supplementary Fig. 1c). Therefore, to identify glucocorticoid-induced gene regulations at an earlier time point, we treated SUP-B15 cells with dexamethasone (1 µM), prednisolone (2 µM), and DMSO for 6 h before collecting total RNA. The experiment was repeated three times and all nine samples were analyzed by Affymetrix Human Gene 2.0 ST array. Deregulated genes were identified by individually comparing expression between dexamethasone- and DMSO-treated samples, and prednisolone- and DMSO-treated samples. Significance Analysis of Microarrays (SAM)^16^ was used to detect the deregulated genes. We used a 5% false discovery rate (FDR) as the cut-off. Most of the deregulated genes were common between dexamethasone- and prednisolone-treated cells (Supplementary Table 1a–d), where 366 common genes were downregulated (Fig. 1a) and 217 common genes were upregulated in drug-treated cells (Fig. 1b). A comparison between dexamethasone- and prednisolone-treated samples did not result in any significantly deregulated genes (data not shown). These data suggest that both dexamethasone and prednisolone act on cells by a similar mechanism. Upregulated (Supplementary Fig. 2a) or downregulated (Supplementary Fig. 2b) genes were analyzed by hierarchical clustering showing several different clusters. However, clusters for dexamethasone- and prednisolone-treated cells remain similar, as observed by SAM. Further analysis of the top-listed commonly upregulated genes also showed similar clusters (Fig. 1c). Several core cellular signaling regulators such as DUSP1, FBXW7, Sprouty family proteins, etc. were upregulated in both dexamethasone- and prednisolone-treated cells in a similar fashion. Furthermore, gene set enrichment analysis (GSEA) identified enrichment of similar signaling pathways in dexamethasone- and prednisolone-treated cells (Fig. 1d, e). Although SUP-B15 displayed potential variations in gene expression in response to glucocorticoids, several glucocorticoid-resistant ALL cell lines such as JURKAT, MOLT-4, and CCRF-CEM did not show any significant difference in gene expression upon treatment with dexamethasone (1 µM) or prednisolone (2 µM, data not shown).

### Dexamethasone treatment induces activation of serine/threonine protein kinases

**Fig. 2:**
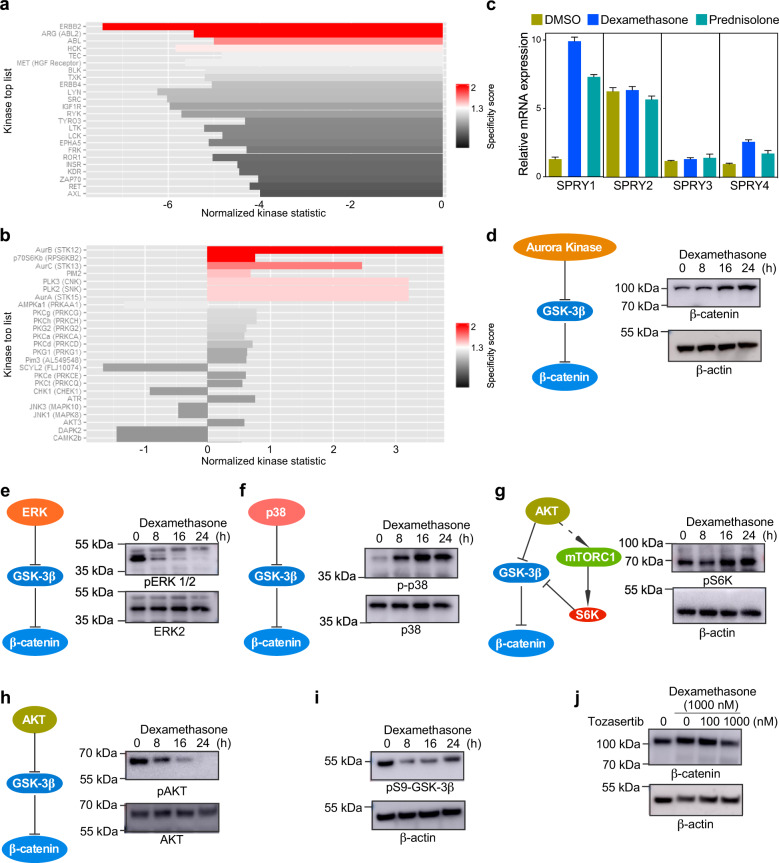
Glucocorticoid-induced regulation of kinase activation. SUP-B15 cells were treated with 1 µM dexamethasone or DMSO for 6 h before lysis. **a** To measure protein tyrosine kinase activity in dexamethasone-treated cells, lysates were applied in Pamgene peptide substrate-based tyrosine kinase array and analyzed for tyrosine kinase activity using Pamgene software. **b** To measure protein serine/threonine kinase activity in dexamethasone-treated cells, lysates were applied in Pamgene peptide substrate-based serine/threonine kinase array and analyzed for serine/threonine kinase activity using Pamgene software. **c** Changes in the expression of Sprouty family genes in dexamethasone and prednisolone-treated SUP-B15 cells compared to DMSO-treated cells. Error bars show standard deviation. **d**–**i** SUP-B15 cells were treated with 1 µM dexamethasone for the indicated time period before lysis. Lysates were analyzed by SDS-PAGE and western blotting using specific antibodies as labeled. **j** SUP-B15 cells were treated with 1 µM dexamethasone and with different concentrations of Tozasertib for 24 h before lysis. Lysates were analyzed by SDS-PAGE and western blotting using specific antibodies as labeled. Blots shown in each panel were from the same experiment and processed similarly.

Protein kinases regulate core cellular signaling by phosphorylating the substrate proteins. Because glucocorticoid treatment potentially deregulated gene expression leading to specific pathway enrichment, we hypothesized that glucocorticoid treatment will also modulate the core cellular signaling by altering the activation of protein kinases. To measure global kinase activity, we used peptide substrate-based kinase profiling. Pamgene peptide substrate-based kinase profiling has been demonstrated to be a powerful technique for determining kinase activity^17,18^. SUP-B15 cells were treated with dexamethasone (1 µM) or DMSO for 6 h before lysis. Lysates were applied to the Pamgene protein tyrosine and protein serine/threonine chips following the manufacturer’s guidelines. While comparing kinase activity enrichment between DMSO and dexamethasone-treated cells, we observed that the activity of protein tyrosine kinases was completely downregulated in dexamethasone-treated cells (Fig. 2a) and the activity of several protein serine/threonine kinases was upregulated (Fig. 2b). To understand the possible mechanisms of how kinase activities were regulated, we used the “negative regulation of protein kinase activity (GO:0006469)” related gene signature to compare gene expression data in dexamethasone- and prednisolone-treated SUP-B15 cells. We observed that SPRY1 expression was more than fivefold upregulated in both dexamethasone- and prednisolone-treated cells (Fig. 2c). Sprouty family proteins include four members (SPRY1-4) that can control receptor tyrosine kinase activation^19,20^. The upregulation of serine/threonine kinase activity, for example, Aurora family kinases, Polo-like kinases (PLKs), p70S6K, and PIM2, seems to be interesting. Further, using a panel of 378 kinase inhibitors, we observed that ALL cell lines that were either sensitive (NALM6 and 697) or resistant (TANOUE and JURKAT) to dexamethasone were highly responsive to inhibitors targeting Aurora kinases, PLKs, and PI3K/mTOR pathway components (Supplementary Fig. 3). Aurora kinase activity can influence several signaling proteins including β-catenin^21^. Because we observed that cells treated with dexamethasone and prednisolone display enrichment in the β-catenin responsive genes (Fig. 1d, e), we reasoned that Aurora kinase may regulate the β-catenin pathway in dexamethasone-treated cells. β-catenin is a component of the canonical WNT signaling pathway where its protein level is maintained by the AXIN/APC/CK1/GSK-3β complex. This complex phosphorylates β-catenin resulting in β-TrCP mediated ubiquitination and, thereby, degradation in proteasome^22^. Aurora kinase directly phosphorylates GSK-3β on serine 9 residue, which reduces the catalytic activity of GSK-3β, resulting in the accumulation of β-catenin^23^. We observed that the β-catenin level was upregulated in dexamethasone-treated cells (Fig. 2d). GSK-3β inactivation can also be mediated by several serine/threonine kinases such as ERK^24^, p38^25^, and AKT^26^. GSEA shows two opposite signatures in the MAP kinase pathway: KRAS downregulation and MEK upregulation (Fig. 1d, e). We observed that while ERK phosphorylation was inhibited upon dexamethasone treatment (Fig. 2e), p38 phosphorylation was upregulated (Fig. 2f). The KRAS downregulation signature is in agreement with the downregulation of ERK phosphorylation in dexamethasone-treated cells. Nevertheless, MEK signaling can activate p38 without affecting ERK phosphorylation at a certain condition^27^. Additionally, dexamethasone-induced activation of p38 was previously described in dexamethasone-sensitive RS4;11 and SUP-B15 cells^28^. Finally, cells treated with dexamethasone displayed an upregulation of S6K phosphorylation (Fig. 2g), similar to the kinase profiling data (Fig. 2b), while it showed a downregulation of AKT phosphorylation (Fig. 2h). S6K is a downstream effector of the PI3K/AKT pathway and can be activated by mTORC1 complex^26^. Likewise, S6K can directly phosphorylate and inactivate GSK-3β^29^. However, dexamethasone-induced S6K phosphorylation is likely to be independent of AKT activation. Because we observed that ERK and AKT phosphorylation was inhibited, while p38 and S6K phosphorylation was upregulated by dexamethasone, we then checked the phosphorylation of their substrate GSK-3β Serine 9 residue. We observed that GSK-3β Serine 9 phosphorylation was initially reduced and then increased over time (Fig. 2i). Furthermore, cells treated with the Aurora kinase inhibitor Tozasertib in the presence of dexamethasone displayed a reduction of β-catenin accumulation (Fig. 2j), suggesting that Aurora kinase activity may be required for β-catenin accumulation in dexamethasone-treated cells. SUP-B15 cells display constitutive activation of ERK (Fig. 2e) and AKT (Fig. 2h), which probably maintains initial GSK-3β Serine 9 phosphorylation. However, later, this might be mediated by Aurora kinase, p38, and S6K. Several other kinases such as protein kinase C (PKC) family proteins, protein kinase A (PKA), ribosomal protein S6 kinase (RSK), etc. can also phosphorylate GSK-3β at Serine 9 residue^30^. We have not tested those kinases in this context and, therefore, we cannot exclude the possibility of their involvement. Collectively, these data suggest that β-catenin protein levels are stabilized by dexamethasone-mediated regulation of multiple signaling pathways.

### Prediction of dexamethasone sensitivity in ALL

**Fig. 3:**
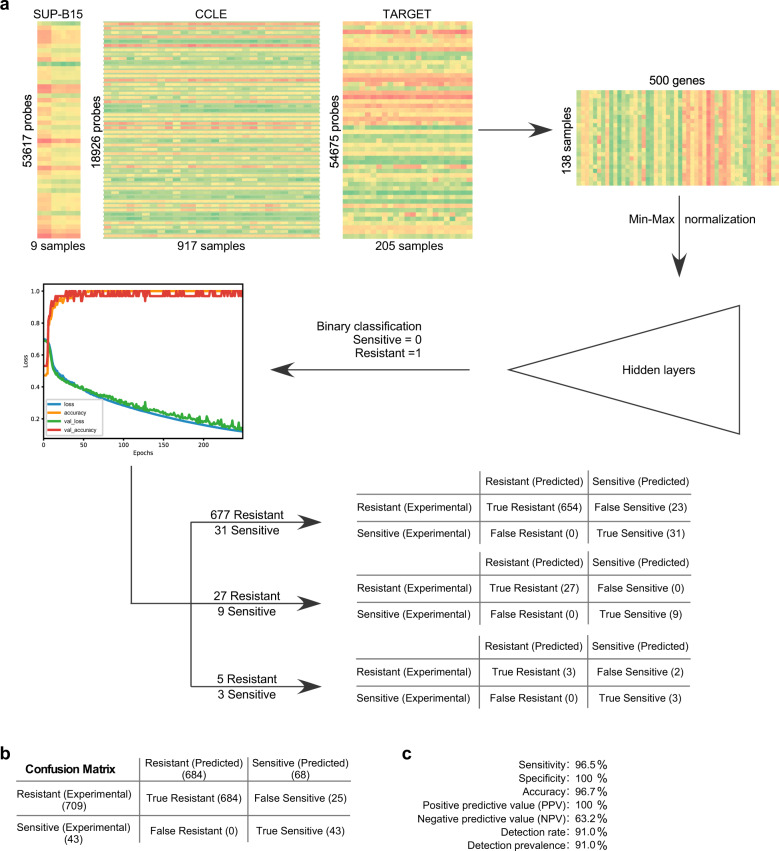
Dexamethasone sensitivity prediction model. **a** Deregulated gene signatures from dexamethasone- and prednisolone-treated SUP-B15 cells were combined with genes displaying the highest level of variation in CCLE and TARGET (ALL) datasets and 500 genes were selected. The 500 genes from 138 cell lines of hematological malignancies were used to build a deep learning model. The model was tested using three sets of samples. **b** Confusion matrix for all three groups of samples. **c** The performance of the model was calculated using three test sample groups.

Because we observed that dexamethasone treatment mediates the deregulation of certain survival signaling pathways, we next aimed to verify the findings in ALL patient materials. The TARGET ALL dataset provides more than 200 annotated samples with gene expression data but is missing dexamethasone sensitivity information. Therefore, we aimed to predict dexamethasone sensitivity in those samples. Various methodologies have been proposed to predict monotherapy response using different modalities including single nucleotide polymorphisms (SNPs), copy number variations (CNVs), mutations, DNA methylation, protein expression, and RNA expression (Reviewed in ref. ^11^). Both the Cancer Cell Line Encyclopedia (CCLE)^31^ and the Genomics of Drug Sensitivity in Cancer (GDSC)^32^ provide drug sensitivity scores and various genetic features for >1000 cell lines. The GDSC dataset lacks drug sensitivity scores for dexamethasone; therefore, we used the CCLE dataset to predict dexamethasone sensitivity. The CCLE dataset contains dexamethasone sensitivity scores for 708 cell lines from different cancers, where 138 cell lines are from hematological malignancies. The use of RNA expression remains the most popular modality for drug sensitivity prediction^9,10^, and therefore, we used RNA expression data to serve the purpose. The CCLE RNA expression dataset contains >18,000 genes for each sample. The use of >18,000 genes with drug sensitivity data for 138 cell lines can perhaps be used to build a prediction model but the model will suffer from poor generalization performance when tested on new data. A high-dimensional dataset with too many features can lead to overfitting such that the model captures both the real and random effects^11^. Low complexity models such as logistic regression, principal component regression, partial least square regression, or support vector machine can handle higher dimensional data but, due to the linear nature of the algorithm, predictions introduce significant modeling bias^11,33,34^. Deep learning models provide superior prediction accuracy over linear models where the number of samples is comparable to the number of features^11^. To increase the ratio between sample and feature, we attempted to reduce the number of genes. We used a combined gene signature (Supplementary Fig. 4) of 500 genes to develop a deep learning model. Dexamethasone sensitivity data for 138 cell lines relating to hematological malignancies were collected from the PharmacoDB database^35^ and gene expression data for those cell lines were collected from the CCLE database. Cell lines with IC_50_ < 700 nM were considered sensitive and cell lines with IC_50_ > 1000 nM were marked as resistant. We used the Keras^36^ sequential model to build the drug sensitivity prediction model. The model was tested using 708 CCLE cell lines data and two small ArrayExpress datasets: E-MTAB-7781 and E-MTAB-9250 (Fig. 3a). When combining all three datasets, the model predicted 684 samples as resistant (out of 709) and 43 samples as sensitive (out of 43; Fig. 3b). The model displayed more than 95% prediction accuracy, while the negative predictive value was 63% (Fig. 3c). This is because, to test the model, we used a larger number of resistant samples as compared to sensitive samples. Overall although the model performed well with these datasets, it has not been thoroughly tested with a large dataset, and therefore, we cannot exclude the possibility that the model might perform poorly with a large unknown dataset. Furthermore, we observed that in a small set of RNAseq data where three samples were experimentally defined as sensitive to dexamethasone and five samples as resistant, the model predicted two samples from the resistant group as to be sensitive (Fig. 3a). To understand why the model failed to detect two samples as resistant, we analyzed the gene expression data (FPKM) from those samples. In this dataset, three sensitive cell lines (697, NALM6, and RS4;11) were treated with dexamethasone to generate dexamethasone-resistant cell lines^5^. The resistant cell line TANOUE was used as a control and was also treated with dexamethasone for the same period. Comparisons between dexamethasone-treated cells and naïve cell lines demonstrated an extremely high correlation of gene expression for 697 (*R*^2^ = 0.9767), NALM6 (*R*^2^ = 0.9581), and TANOUE (*R*^2^ = 0.9929), while RS4;11 displayed a comparatively lower correlation (*R*^2^ = 0.5977) (Supplementary Fig. 5a). This suggests that the model expects some differences in gene expression between sensitive and resistant cell lines. This is also evident by the fact that TANOUE and other sensitive cell lines displayed a moderate correlation (Supplementary Fig. 5b). However, the model could efficiently detect sensitive cells even though there was a moderate correlation between them (Supplementary Fig. 5c).

### Dexamethasone sensitivity prediction in ALL patients using the deep learning model

**Fig. 4:**
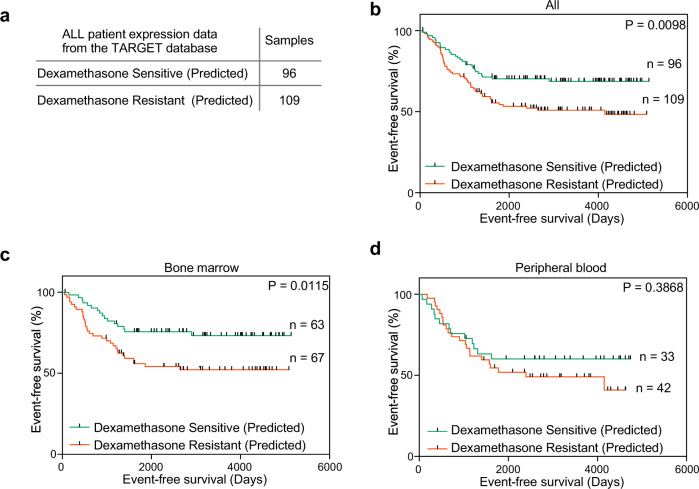
Dexamethasone sensitivity prediction in ALL patient samples. The TARGET dataset for ALL was used to predict dexamethasone sensitivity. **a** In total 205 patient samples were used to predict dexamethasone sensitivity by the deep learning model. **b** Event-free survival between dexamethasone-sensitive and -resistant groups using 205 ALL patient samples was determined by GraphPad Prism. **c** Event-free survival for ALL patient samples collected from bone marrow. **d** Event-free survival for ALL patient samples collected from peripheral blood.

We used the ALL patient dataset from the TARGET database to predict dexamethasone sensitivity. The gene signature (500 genes) from 205 ALL patients was used to predict dexamethasone sensitivity. The model predicted 96 patient samples as dexamethasone sensitive and 109 samples as dexamethasone-resistant (Fig. 4a). Because dexamethasone sensitivity can predict patient survival^37–39^, we then compared event-free survival between the dexamethasone-sensitive and -resistant groups. We observed that the dexamethasone resistant group had significantly (*p* = 0.0098) reduced event-free survival (Fig. 4b). The TARGET ALL dataset contains samples that have been classified as those obtained from bone marrow and peripheral blood. Thus, we further determined the difference in event-free survival by dividing the patients into bone marrow and peripheral blood groups. Interestingly, a significant survival difference was observed in the bone marrow group (Fig. 4c), but not in the peripheral blood group (Fig. 4d). However, this observation must be interpreted carefully, as the peripheral blood group contains a smaller number of patients.

### The dexamethasone-resistant ALL patient group displays similar pathway enrichment as the glucocorticoid-treated cells

**Fig. 5:**
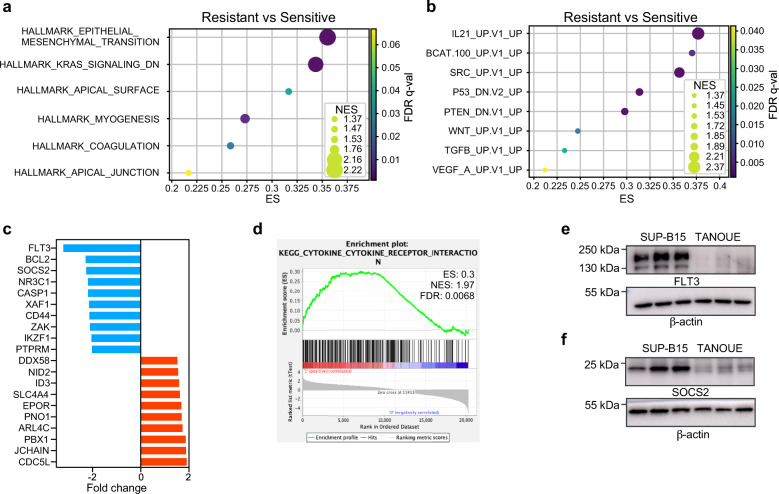
Pathway enrichment in dexamethasone-resistant ALL patient samples. Pathway enrichment in dexamethasone-resistant ALL patient samples was analyzed using GSEA. **a** Hallmarks and **b** Oncogenic signatures gene sets were used for pathway enrichment analysis. **c** Upregulated and downregulated genes in dexamethasone-resistant ALL patient samples were determined by SAM. The bar graph shows selected top-listed genes. All the upregulated and downregulated genes are included in supplementary table T2. **d** The enrichment of KEGG cytokine and cytokine receptor interaction pathway in dexamethasone-resistant ALL patient samples was determined by GSEA. **e**, **f** SUP-B15 and TANOUE cells were lysed. Lysates were analyzed by SDS-PAGE and western blotting using specific antibodies as labeled. Blots shown in each panel were from the same experiment and processed similarly.

Next, we compared gene signatures between the predicted dexamethasone-sensitive and -resistant patient groups. We used ALL samples from bone marrow to identify pathway enrichment. Epithelial–mesenchymal transition (EMT) was found to be highly enriched in samples from dexamethasone-resistant patients (Fig. 5a), which was similar to that of dexamethasone-treated SUP-B15 cells (Fig. 1d). Several other interesting pathways that were enriched included PTEN-DN, β-catenin, TGF-β, and WNT upregulation signatures (Fig. 5b). Similar pathways were found to be enriched in dexamethasone-resistant ALL cell lines (Supplementary Fig. 6a, b). Further, we observed the downregulation of several genes including *FLT3, BCL2, SOCS2*, etc. in samples from dexamethasone-resistant ALL patients (Fig. 5c). Additionally, enrichment of the KEGG Cytokine-Cytokine Receptor Interaction gene signature was observed in samples from dexamethasone-resistant ALL patients (Fig. 5d), which is in line with the observation that SOCS2 expression is downregulated as SOCS family members act as negative regulators of cytokine receptor signaling^40^. To verify the observation that several genes were downregulated in samples from dexamethasone-resistant ALL patients, we compared the expression of FLT3 and SOCS2 in dexamethasone-sensitive SUP-B15 and -resistant TANOUE cells. Data are in line with the findings from patient samples, in that FLT3 (Fig. 5e) and SOCS2 (Fig. 5f) were downregulated in TANOUE cells. Although we observed that FLT3 expression was downregulated in predicted dexamethasone-resistant ALL patient samples, in vitro development of dexamethasone resistance in cell lines using long-term culture retained FLT3 expression in resistant cells and even selected for oncogenic FLT3 mutations^5^. Therefore, it is likely that the loss of FLT3 expression occurs in a portion of ALL patients and might not be a common feature for all dexamethasone-resistant ALL samples.

### Several kinase inhibitors display synergy with dexamethasone in dexamethasone-resistant cells

**Fig. 6:**
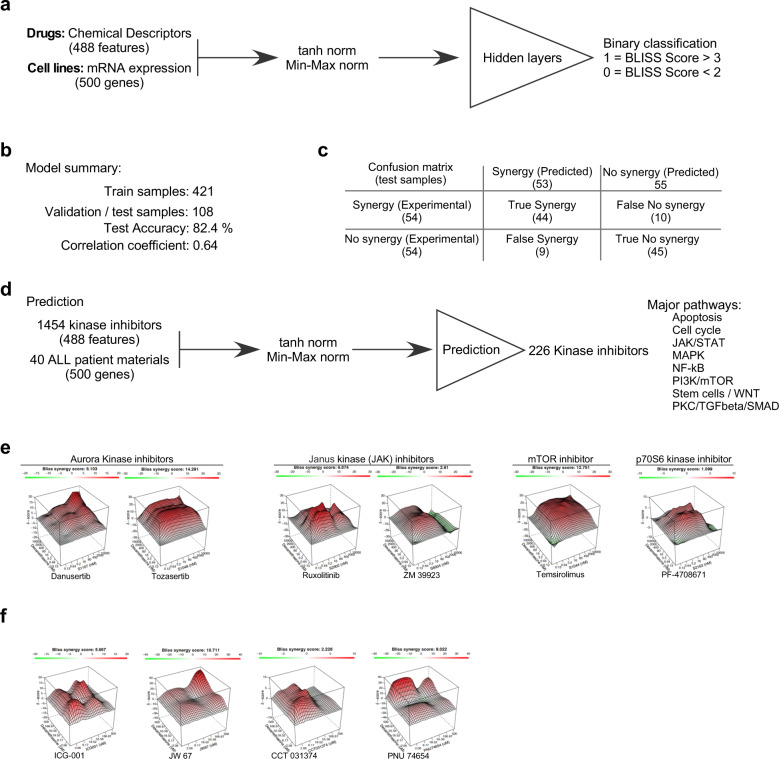
The synergy between dexamethasone and kinase inhibitors. **a** Using the same 500-gene signature and drug synergy data from the DrugComb database, a deep learning binary classification model was developed to predict synergy between dexamethasone and other inhibitors. **b** Model performance was tested using 108 test samples. **c** Confusion matrix showing the model’s performance using 108 test samples. **d** In silico synergy prediction between dexamethasone and 1454 kinase inhibitors using the deep learning model. **e** In vitro synergy measurement between dexamethasone and kinase inhibitors in TANOUE cells using Cell Titer Glo after 48 h of incubation with drug combinations. The figure shows representative kinase inhibitors. **f** In vitro synergy measurement between dexamethasone and β-catenin inhibitors in TANOUE cells using Cell Titer Glo after 48 h of incubation with drug combinations.

Next, we sought to identify drugs that can overcome dexamethasone resistance. We attempted to develop a method for predicting synergy between dexamethasone and other drugs. For this, we used drug synergy data from the DrugComb database. We used 488 chemical descriptors to describe each drug and the 500-gene signature to describe each cell line. Data were normalized before being introduced to the deep learning model. Because we had a limited number of cell lines and drug combinations, we used binary classification such that, for drug combinations, a BLISS score >3 was considered synergy (defined as 1), and a BLISS score <2 was considered no synergy (defined as 0; Fig. 6a). Therefore, the model was trained only for the absence or presence of synergy. We were able to build a model with 82.4% test accuracy (correlation coefficient = 0.64; Fig. 6b). The deep learning model achieved a moderate level of prediction accuracy (Fig. 6c). Further, to predict synergy, we used 1454 kinase inhibitors and 40 ALL patient samples from the TARGET dataset. The model predicted synergy between dexamethasone and 226 inhibitors that inhibit kinases involved in regulating apoptosis, the cell cycle, JAK/STAT, MAPK, NFκ-B, PI3K/mTOR, Stem cells/WNT, and PKC/TGFβ/SMAD pathways (Fig. 6d). Because we used a relatively small amount of data to build and test the model, it may display poor prediction performance. To verify the synergy between dexamethasone and these kinase inhibitors, we used 38 kinase inhibitors from those groups in combination with dexamethasone to analyze synergy in the dexamethasone-resistant TANOUE cell line. We observed that several, but not all, kinase inhibitors displayed synergy with dexamethasone in TANOUE cells (Supplementary Fig. 7 and Fig. 6e). Inhibitors displaying higher synergy scores include Aurora kinase, JAK, mTOR, and S6K inhibitors (Fig. 6e). Several of these kinases are known to be involved in β-catenin stabilization^23,25,29,41–45^, therefore, we tested whether β-catenin inhibition synergizes with dexamethasone. We observed that several inhibitors targeting β-catenin activity display synergy with dexamethasone in TANOUE cells (Fig. 6f).

## Discussion

Glucocorticoids form the cornerstone of drugs in the chemotherapeutic regimen cocktail for the treatment of ALL and serve as one of the strongest predictors of relapse in ALL^46^. However, the molecular mechanisms that contribute to glucocorticoid resistance and relapse in ALL are quite divergent, and also remain poorly understood^46,47^. In this study, we used transcriptomic and peptide substrate-based kinase profiling, which was supplemented with the deep learning method to define deregulated cellular signaling pathways in dexamethasone-resistant ALL. We observed that dexamethasone resistance is mediated through the activation of multiple parallel signaling pathways.

Six hours of dexamethasone treatment induced a gene signature mimicking the loss of PTEN function (Fig. 1d), which suggests that dexamethasone treatment might release a negative feedback loop on PI3K signaling. Additionally, TGF-β pathway enrichment (Fig. 1d) is linked to the PTEN suppression phenotype^48^. Enrichment of those pathways were also present in prednisolone-treated cells (Fig. 1e) and in patient samples predicted to be dexamethasone-resistant (Fig. 5b). Furthermore, peptide substrate-based kinase profiling (Fig. 2b) and western blot using a phospho-specific antibody (Fig. 2g) showed upregulation of S6K activity probably related to the activation of the PI3K/mTOR pathway. The PI3K pathway plays a role in dexamethasone resistance in acute lymphoblastic leukemia and inhibition of AKT or mTOR reverses dexamethasone resistance^49,50^. However, dexamethasone treatment inhibited AKT phosphorylation (Fig. 2h) and we have not seen an enrichment of the PI3K/mTOR gene signature in GSEA raising the possibility that S6K activation may be mediated by an alternative pathway. For example, Aurora kinase activation is linked to the activation of S6K^43^.

Another interesting pathway that was enriched is involved in epithelial-mesenchymal transition (Figs. 1d, e and 5a). In this process, epithelial markers are downregulated while mesenchymal markers get upregulated, a typical feature found in embryogenesis, organ development, and predominantly epithelial cancer^51,52^. Several EMT-related transcription factors are also known to be expressed in hematological cancers and thereby modulate cell proliferation, viability, stemness, and drug resistance^53^. It has been shown that dexamethasone favored EMT and cancer progression in pancreatic cancer^54^. Aurora kinase activity has been linked to EMT in several epithelial cancers^55–57^. Our kinase activity profiling data suggest a strong activation of Aurora kinase activity in dexamethasone-treated cells (Fig. 2b), linking to the enrichment of EMT markers. The role of other cell cycle regulatory kinases such as PLK family proteins in EMT has been poorly studied. Studies suggest a role of PLK1 in EMT while the role of other family members in EMT, such as PLK2 and PLK3, remains relatively unknown^58^. However, a link has been established between PLK2 expression and Cyclin E stabilization where PLK2 induces FBXW7 degradation^59^. FBXW7 binds to cyclin E and directs it for proteolytic degradation^60^. Finally, enrichment of WNT and its canonical effector, the β-catenin pathway (Fig. 5b), also links to EMT^61^.

Enrichment of cytokine and cytokine receptor signaling pathways was observed in patient materials predicted to be dexamethasone-resistant (Fig. 5d). This is probably because *SOCS2* expression was downregulated in dexamethasone-resistant ALL patient samples (Fig. 5c), which was also evident in a dexamethasone-resistant cell line (Fig. 5f). SOCS2 belongs to the suppressor of the cytokine signaling family of proteins and acts as a negative regulator of cytokine signaling^40^. In silico identification of synergy between dexamethasone and kinase inhibitors provides additional support to the activation of distinct signaling pathways (Fig. 6d). Furthermore, the in vitro drug synergy experiment to follow up in silico data is in line with the fact that inhibition of JAK, mTOR, S6K, and cell cycle regulatory kinases including Aurora kinases, displays a synergistic effect with dexamethasone in a dexamethasone-resistant cell line (Fig. 6e).

While combining all the data, we observed that enrichment of the β-catenin upregulation signature is common within all four GSEAs (Figs. 1d, e and 5b, and Supplementary Fig. 6b), suggesting a link between dexamethasone resistance and transcriptional activation of β-catenin target genes. β-catenin activity is regulated by stabilization of its protein levels through inactivation of the destruction complex (AXIN/APC/CK1/GSK-3β) that phosphorylates β-catenin, resulting in ubiquitin-mediated degradation^22^. Aurora kinases play important roles in the inactivation of the destruction complex by inducing phosphorylation-dependent inactivation of GSK-3β. This process can take place via several different mechanisms. GSK-3β is a substrate of Aurora kinase^23^. It can also directly or indirectly activate JAK2^41^, p38^42^, and S6K^43,44^. While inhibition of JAK2 activity suppressed β-catenin accumulation^45^, S6K^29^, and p38^25^ directly phosphorylate GSK-3β and negatively regulate its activity. We also observed that inhibitors targeting Aurora kinases, JAK, mTOR, and S6K display synergy with dexamethasone (Fig. 6e), further suggesting that Aurora kinase activity may be involved in dexamethasone resistance. A link between Aurora kinase activity and dexamethasone sensitivity was described where the pharmacological inhibition of Aurora kinase enhanced dexamethasone-induced expression of cell death genes^62^.

Collectively, our data suggest that stabilization of β-catenin through phosphorylation-dependent inactivation of GSK-3β likely by Aurora kinases and their direct or indirect downstream effectors such as JAK, mTOR, p38, and S6K may contribute to dexamethasone resistance (Supplementary Fig. 8). These kinases are possibly activated during the initial exposure to dexamethasone that may be contributed to dexamethasone-resistant leukemia patients. Finally, drugs targeting these kinases and their possible downstream effector β-catenin can partially restore dexamethasone sensitivity.

## Method

### Gene expression analysis

ALL cell lines SUP-B15, JURKAT, MOLT-4, and CCRF-CEM were treated with 1 µM dexamethasone or 2 µM prednisolone for 6 h before lysis. All cell lines were collected from DSMZ (Braunschweig, Germany). Cells were maintained in the recommended medium by DSMZ and were regularly tested for mycoplasma contamination. Total RNA was extracted using the RNeasy mini kit (Qiagen) and the quality of total RNA was checked by Bioanalyzer. Affymetrix Human Gene 2.0 ST Array was used for expression analysis. Raw data were processed for robust multi-array average (RMA) normalization. Significance Analysis of Microarrays (SAM) was used to identify the deregulated genes^16^, with the SAMR version developed by Michael Seo being used^63^. A maximum false discovery rate (FDR) cut-off of 5% was used to define significantly deregulated genes. Gene Set Enrichment Analysis (GSEA) software^64^ (Broad Institute) with the gene sets database MSigDB v7.0 (Hallmarks and Oncogenic signatures) was used for pathway enrichment analysis. ALL patient data were downloaded from the TARGET database^63^. CCLE cell line data were collected from GSE36133^65^.

### Peptide substrate-based kinase profiling

Kinase activity in dexamethasone-treated SUP-B15 cells was measured using the peptide substrate-based kinase profiling (Pamgene, ‘s-Hertogenbosch, the Netherlands) method. SUP-B15 cells were treated with 1 µM dexamethasone for 6 h before lysis. Tyrosine kinase profiling and serine/threonine kinase profiling were performed separately using standard protocols provided by the manufacturer.

### Western blot

Cells were lysed in 1% Triton X-100 lysis buffer, supplemented with protease/phosphatase inhibitors (Na_3_VO_4_, Trasylol, and PMSF). The protein concentration of the total cell lysates was determined by the bicinchoninic acid (BCA) assay method (ThermoFisher Scientific, USA). The lysates were then mixed with a 1:1 volume of SDS sample loading buffer, and 10 µg proteins from each lysate were separated on SDS-PAGE gels; they were then transferred to polyvinylidene difluoride (PVDF) membranes. The membranes were immunoblotted with different primary antibodies. The anti-phospho-ERK1/2 (1:2000), anti-phospho-S6K (1:1000), anti-phospho-AKT (1:1000), and anti-phospho-p38 (1:2500) antibodies were obtained from Cell Signaling Technologies, USA. The anti-ERK (1:200), anti-AKT (1:1000), and anti-β-actin-HRP (1:2000) antibodies were from Santa Cruz Biotechnology, USA. The anti-β-catenin (1:1000) and anti-p38 (1 µg/ml) antibodies were from BD Biosciences, USA. The anti-FLT3 (1 µg/ml)^66^ and anti-SOCS2 (1 µg/ml)^67^ antibodies were described previously. The anti-phospho-serine 9-GSK-3β (1:1000) antibody was from ThermoFisher Scientific, USA. For immunodetection, the blots were incubated with the respective horseradish peroxidase-conjugated secondary antibodies and developed with the Luminata Forte Western HRP Substrate (Millipore) and Amersham Imager 600 (GE Healthcare, Sweden). Where applicable, all blots were derived from the same experiment and were processed in parallel.

### Deep learning models

To develop the binary classification model, 138 cell lines related to hematological malignancies were selected from the CCLE database^65^. The IC_50_ value for dexamethasone was used to define sensitivity. Cells with IC_50_ values <700 nM were classified as sensitive, while cells with IC_50_ values >1000 nM were classified as resistant. With these criteria, 108 cell lines were classified as resistant (defined as 1) and 30 cell lines were classified as sensitive (defined as 0). Because the number of resistant cell lines was higher than the number of sensitive cell lines, we used linear combinations of gene expression for sensitive cell lines to increase the number of samples in the sensitive cell line group. The sequential model from Keras^36^ in TensorFlow^68^ backend was used to develop the deep learning model. The model was trained using a 500-gene signature after Min-Max normalization. For prediction, a 500-gene signature from unknown samples was collected and normalized using Min-Max normalization. Further, to develop a model that can predict synergy between dexamethasone and other drugs, we retrieved synergy data from the DrugComb database^69^. Considering the amount of data, we used binary classification to build the prediction model. We applied BLISS score >3 as synergy (defined as 1) and BLISS score <2 as no synergy (defined as 0). We used data from 529 combinations (29 cell lines and 23 drugs) following the classification (263 samples with BLISS score >3 and 266 samples with BLISS score <2). PyChem^70^ and DeepChem^71^ were used to define features for drugs (total 488 features) and 500-gene signature for cell lines. Data were normalized twice: first using tanh normalization and then by Min-Max normalization. The sequential model from Keras in the TensorFlow backend was used to develop the model. Due to the limited amount of data available, we used 421 samples as training samples and 108 samples as validation/test samples. Then we applied this model to predict synergy between dexamethasone and 1454 kinase inhibitors (information collected from Selleckchem) for 40 ALL patient data from the TARGET dataset. Similarly, we used 488 features to describe each kinase inhibitor and a 500-gene signature for each patient sample for the prediction.

### In vitro drug synergy

For in vitro synergy analysis, 38 kinase inhibitors and a negative control (dinaciclib, predicted as no synergy) were used. A fivefold dilution (10000-0.128 nM) of the single drugs was used. We used a 1:1 combination of 8 doses for synergy and then used Decrease^72^ to predict the full combination (8 × 8). Synergy was calculated using the SynergyFinder web application^73^. We applied a similar method (6 doses) to measure synergy between dexamethasone and β-catenin. Cells were seeded in a 384-well plate and treated with the drug(s) for 48 h. Cell Titer-Glo (Promega, USA) was used to measure cell viability following the manufacturer’s protocol.

### Cell viability and apoptosis

Cells were seeded in a 96-well plate and treated with the drug or DMSO for 48 h. PrestoBlue (ThermoScientific, USA) was used to measure cell viability following the manufacturer’s protocol. For apoptosis assay, cells were seeded in a 24-well plate and treated with the drug or DMSO for different time points. Apoptotic cells were quantified using the annexin-V/7-AAD kit (BD Biosciences, USA) following the manufacturer’s protocol.

### Reporting summary

Further information on research design is available in the Nature Research Reporting Summary linked to this article.

## Supplementary information

Supplementary figures and tables

Reporting summary

## Data Availability

The data generated and analysed during this study are described in the following data record: 10.6084/m9.figshare.13475916^74^. The gene expression data are openly available in ArrayExpress at https://identifiers.org/arrayexpress:E-MTAB-9250^75^. The following additional data files underlying Figs. 2, 5, and 6, and Supplementary Figs. 1 and 7 are openly available in figshare at 10.6084/m9.figshare.13414706^74^: Uncropped_WB_Figure_2d_j.pdf, Uncropped_WB_Figure_5e_f.pdf, Synergy_data.xlsx, Upstream Kinase Score_PTK_Figure_2a.png, Upstream Kinase Score_STK_Figure_2b.png, SUP-B15_EC50.pzf.

## Code availability

Python 3.7 was used to analyze data by using Tensorflow (version 2.1.0), Rdkit (version 2020.03.2), DeepChem (version 2.1.1), Scikit-learn (0.21.3) and Matplotlib (version 3.1.1). Jupyter Notebook codes are available upon request.
