# Supplementary material for: The Aurora kinase/β-catenin axis contributes to dexamethasone resistance in leukemia
Source: NPJ Precis Oncol. 2021 Feb 17;5:13. doi: 10.1038/s41698-021-00148-5 (PMC7889633; doi:10.1038/s41698-021-00148-5)
Supplement: Supplementary file 1 — Supplementary figures and tables [file 41698_2021_148_MOESM1_ESM.pdf]

**The Aurora kinase/β-catenin axis contributes to dexamethasone resistance in leukemia**

Kinjal Shah, Mehreen Ahmed and Julhash U. Kazi

**Supplementary Figures and Tables**

Supplementary Figures: (Pages: 2 – 9)

**Supplementary Figure 1.** Cell viability and apoptosis upon dexamethasone and prednisolone treatment (Page: 2).
**Supplementary Figure 2.** Heatmap displaying clusters of upregulated/downregulated genes in dexamethasone and prednisolone-treated SUP-B15 cells (Page: 3).
**Supplementary Figure 3.** Inhibition of cell cycle regulatory kinases inhibits the viability of ALL cells (Page: 4).
**Supplementary Figure 4.** A flowchart describing the procedure of selecting 500 genes to predict dexamethasone sensitivity (Page: 5).
**Supplementary Figure 5.** Correlation between dexamethasone-sensitive and -resistant cell lines using a 500-gene signature (Page: 6).
**Supplementary Figure 6.** Pathway enrichment in dexamethasone-resistant ALL cell lines (Page: 7).
**Supplementary Figure 7.** Drug synergy (Page: 8).
**Supplementary Figure 8.** The combined analysis identifies Aurora kinase and downstream effector kinases stabilizing β-catenin expression. (Page: 9).

Supplementary Tables: (Pages: 10 – 77)

**Supplementary Table 1a.** Genes upregulated in dexamethasone-treated SUP-B15 cells (Pages: 10 – 17).
**Supplementary Table 1b.** Genes downregulated in dexamethasone-treated SUP-B15 cells (Pages: 18 – 31).
**Supplementary Table 1c.** Genes upregulated in prednisolone-treated SUP-B15 cells (Pages: 32 – 37).
**Supplementary Table 1d.** Genes downregulated in prednisolone-treated SUP-B15 cells (Pages: 38 – 47).
**Supplementary Table 2a.** Upregulated genes in predicted dexamethasone-resistant ALL patient samples (Pages: 48 – 49).
**Supplementary Table 2b.** Downregulated genes in predicted dexamethasone-resistant ALL patient samples (Pages: 50 – 77).

**Uncropped western blots for figures 2d-j and 5e-f (Page: 78 – 79).**

Uncropped western blots for figures 2d-j (Page: 78)
Uncropped western blots for figures 5e-f (Page: 79)

**Supplementary Figure 1. Cell viability and apoptosis upon dexamethasone and prednisolone treatment.** SUP-B15 cell line was treated with ten different concentrations of dexamethasone (0.5 nM to 10 µM) or prednisolone (1.0 nM to 20 µM) for 48h. Cell viability was measured using the PrestoBlue cell viability assay. (a) Absolute EC50 values were calculated using a 4-parameter Hill equation incorporated in GraphPad. (b) Cell viability data from 1.1 µM dexamethasone and 2.2 µM prednisolone-treated cells (as determined in A) were used to make the comparisons. (c) Cells were treated with 1 µM dexamethasone of 2µM prednisolone for different time points. Apoptosis was measured using the Annexin-V/7-AAD apoptosis kit. One-way ANOVA with Bonferroni's multiple comparison test (GraphPad Prism) was used to determine P values. ns, $p > 0.05$; ***, $p < 0.001$.

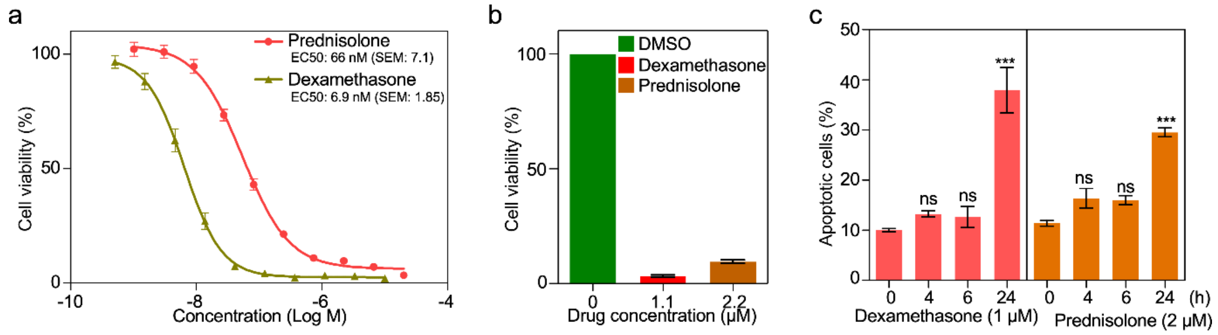

**Supplementary Figure 2. Heatmap displaying clusters of upregulated/downregulated genes in dexamethasone and prednisolone-treated SUP-B15 cells.** The heatmap was generated by the heatmap.2 function of the Gplots library in R. Heatmap displaying clusters of upregulated (a) and downregulated (b) genes in dexamethasone- and prednisolone-treated cells.

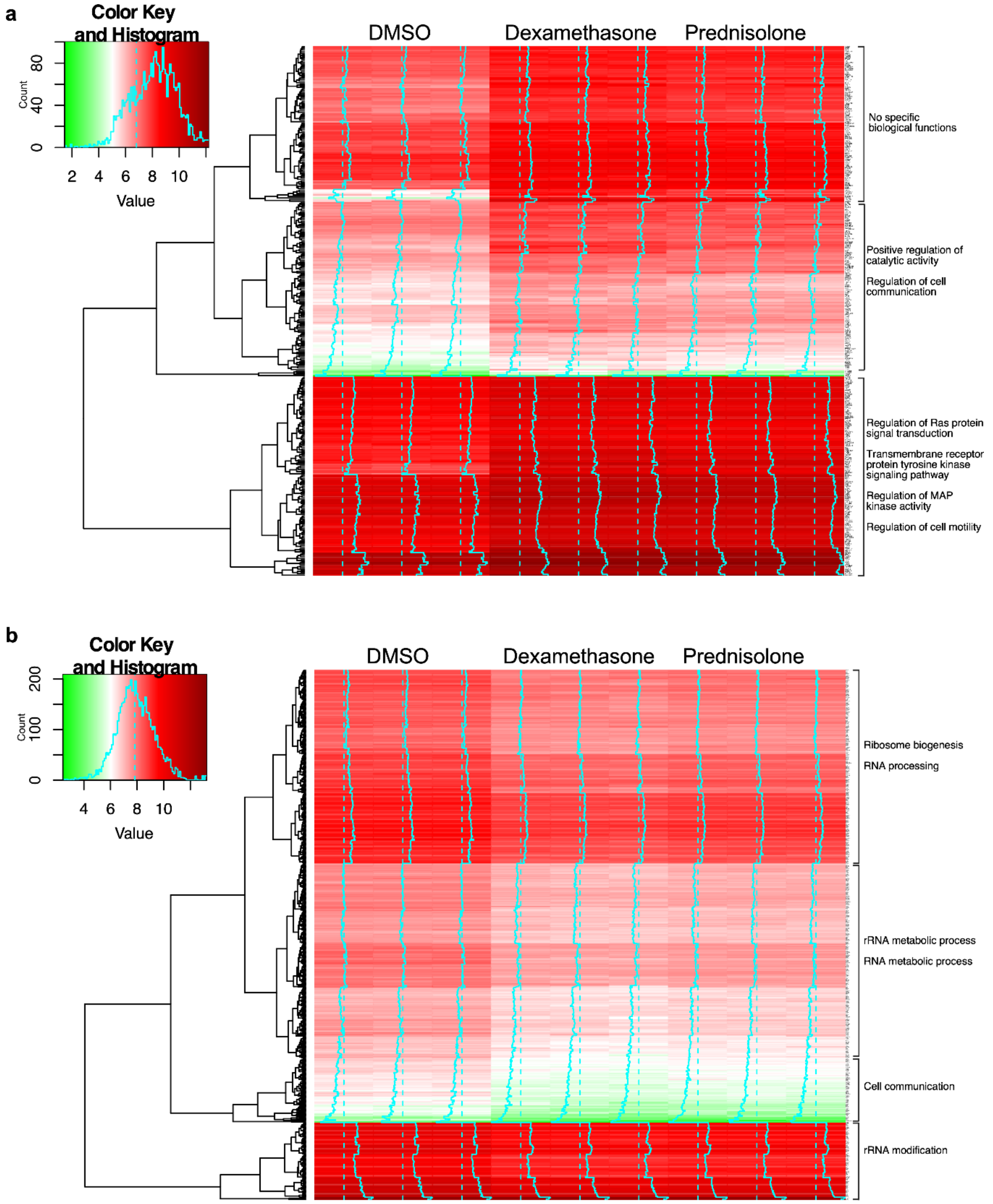

**Supplementary Figure 3. Inhibition of cell cycle regulatory kinases inhibits the viability of ALL cells.** NALM-6, 697, TANOUE and JURKAT cell lines were treated with 100 nM concentration of each kinase inhibitors. Cell viability was measured after 48 h incubation by adding PrestoBlue.

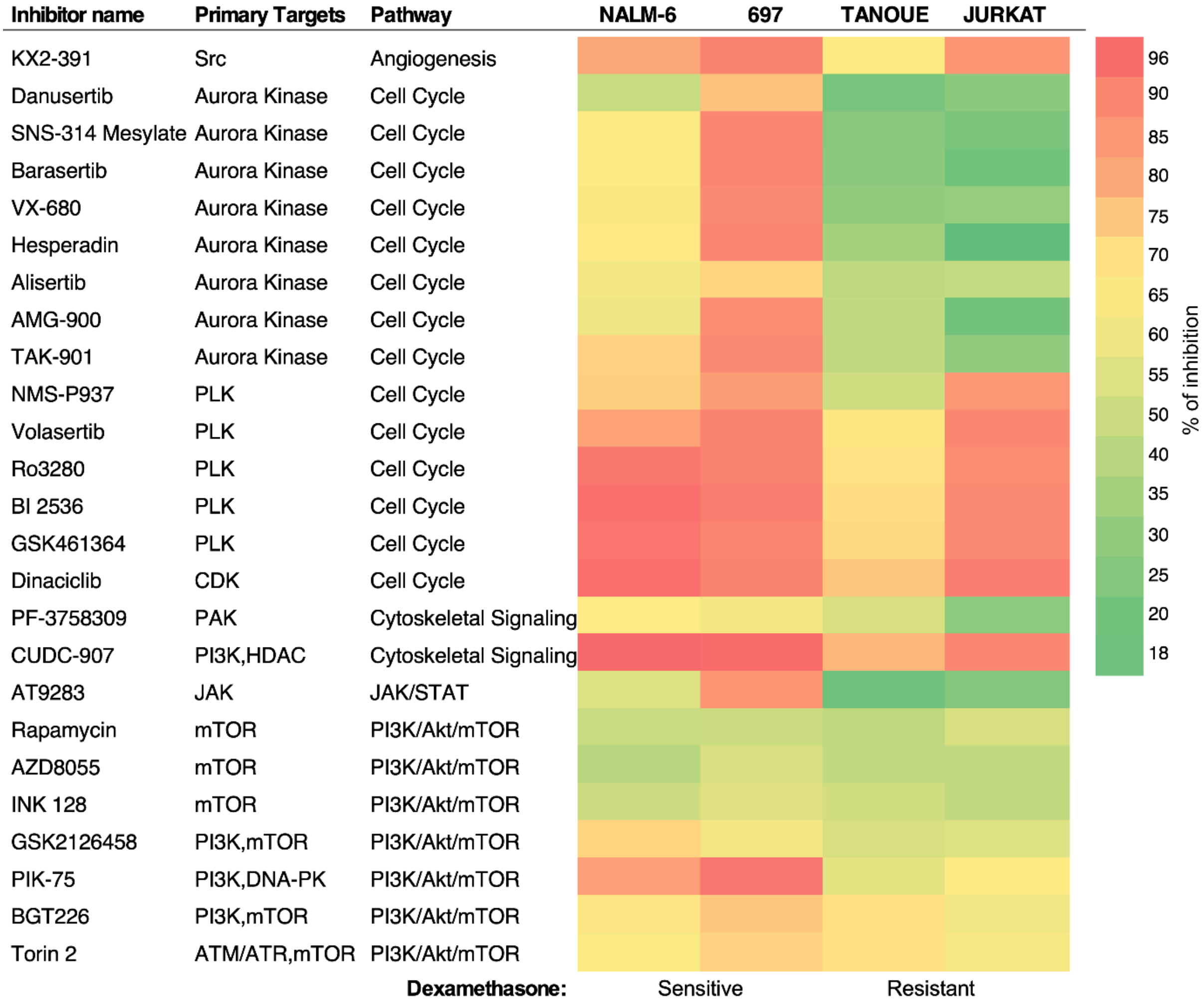

**Supplementary Figure 4. A flowchart describing the procedure of selecting 500 genes to predict dexamethasone sensitivity.** Deregulated genes from both dexamethasone and prednisolone treatment groups (in SUP-B15 cells) were combined (956 genes). This gene signature was combined with the deregulated genes from the CCLE (917 cell lines) and TARGET ALL (205 patient samples) datasets. Each probe from CCLE and TARGET datasets was first analyzed for median absolute deviation (MAD). Probes with MAD values more than mean MAD values were further considered for analysis. These three datasets were then combined and overlapping genes were selected that allowed us to construct a 500-gene signature to be used.

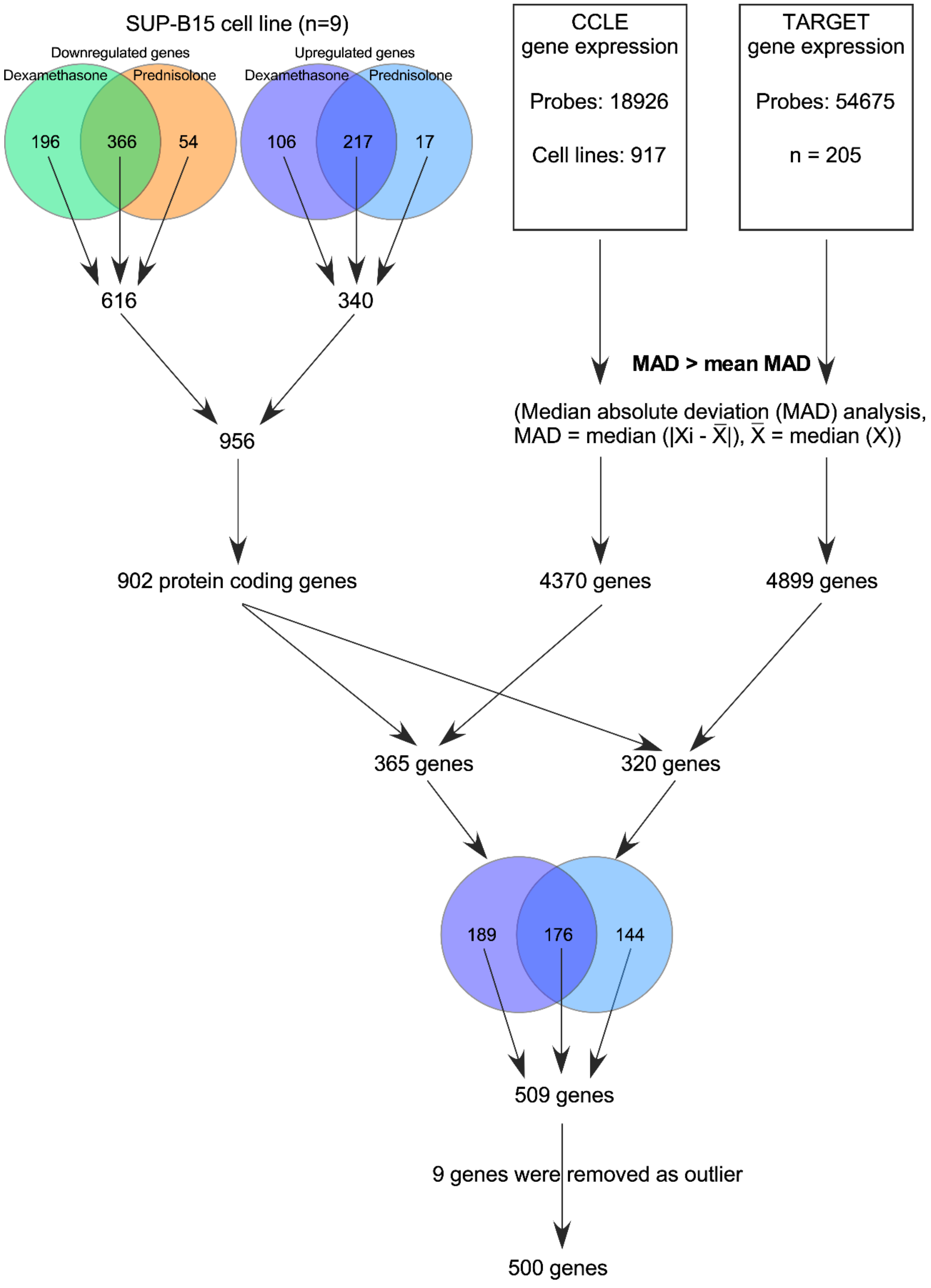

**Supplementary Figure 5. Correlation between dexamethasone-sensitive and -resistant cell lines using a 500-gene signature**. (a-c) FPKMs of 500 genes were plotted as described. The correlation was calculated by GraphPad Prism.

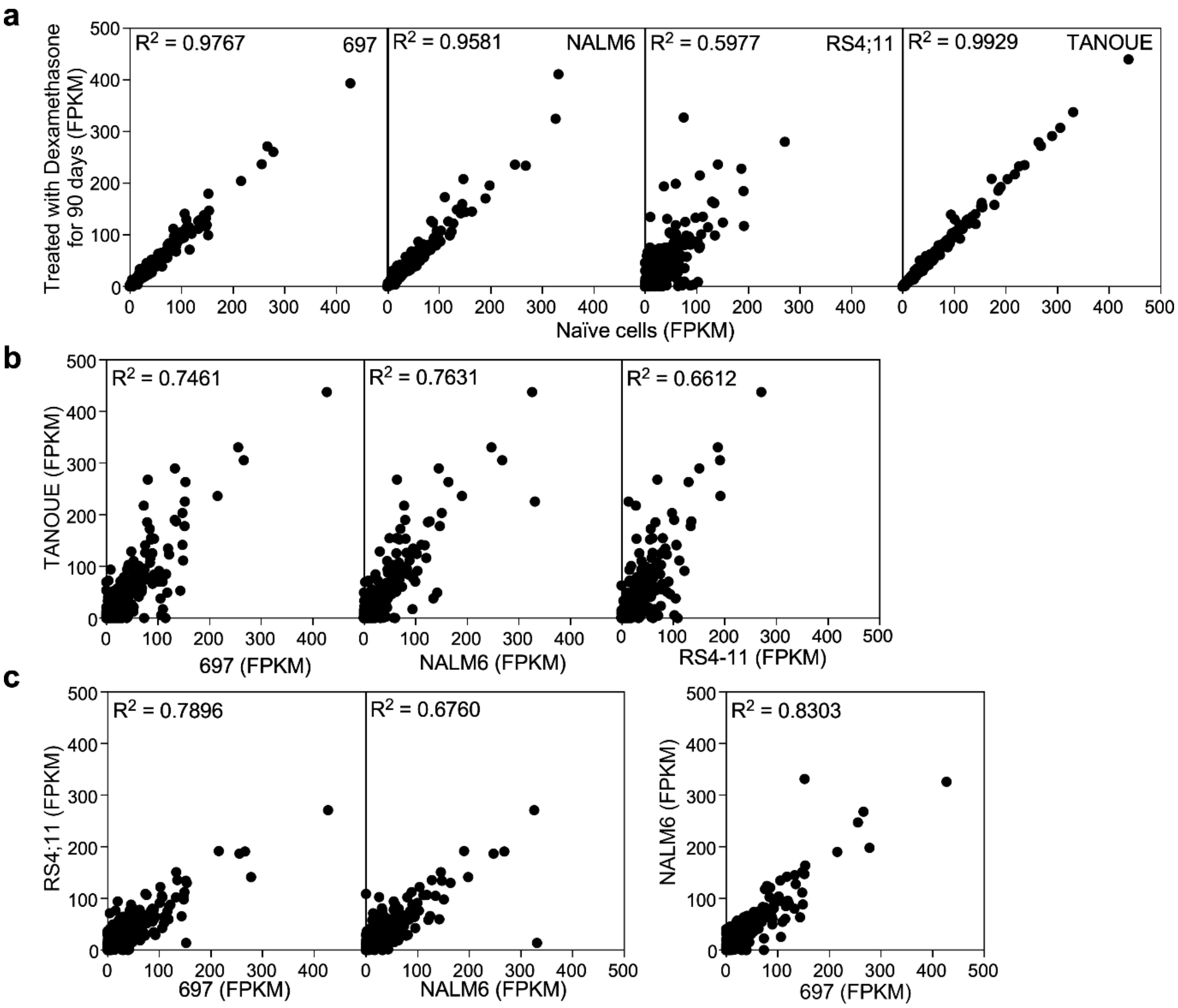

**Supplementary Figure 6. Pathway enrichment in dexamethasone-resistant ALL cell lines.** Pathway enrichment in dexamethasone-resistant ALL cell lines was analyzed using GSEA. (a) Hallmarks and (b) Oncogenic signatures gene sets were used for pathway enrichment analysis.

a

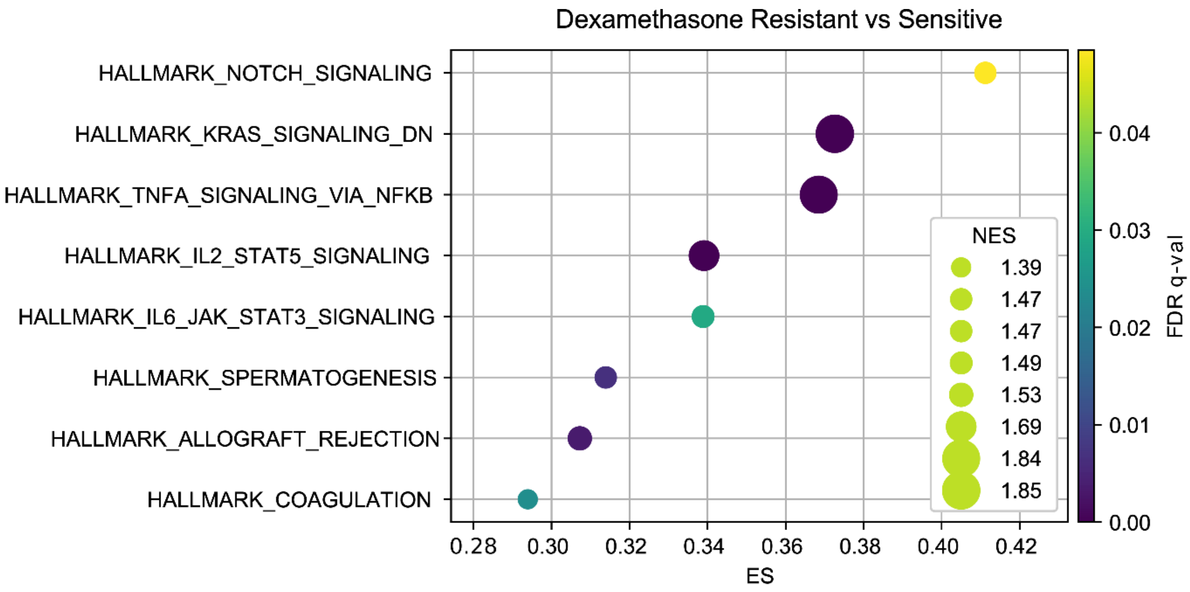


b

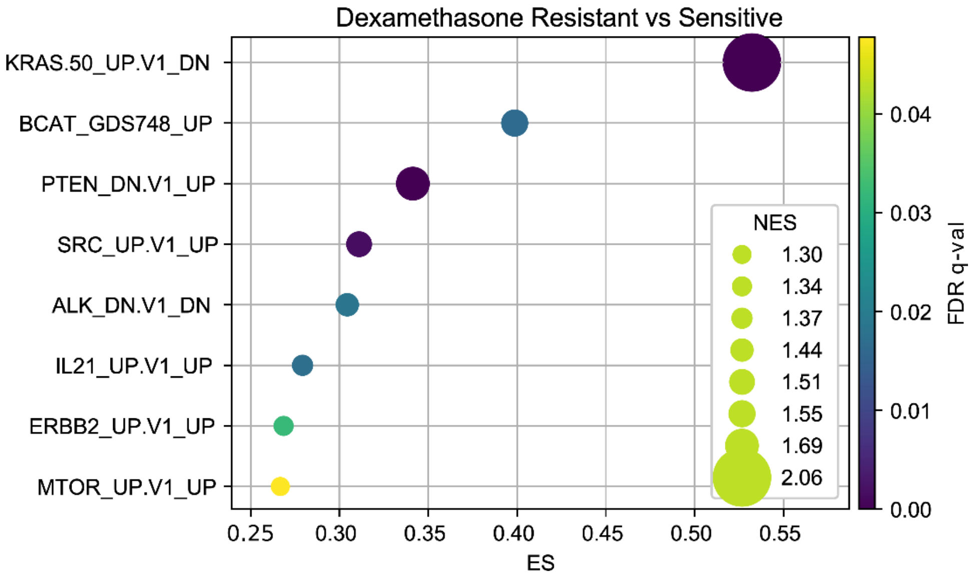

**Supplementary Figure 7. Drug synergy.** *In vitro* synergy measurement between dexamethasone and 38 kinase inhibitors (6 included in figure 6e) in TANOUE cells using Cell Titer Glo after 48h of incubation with drug combinations.

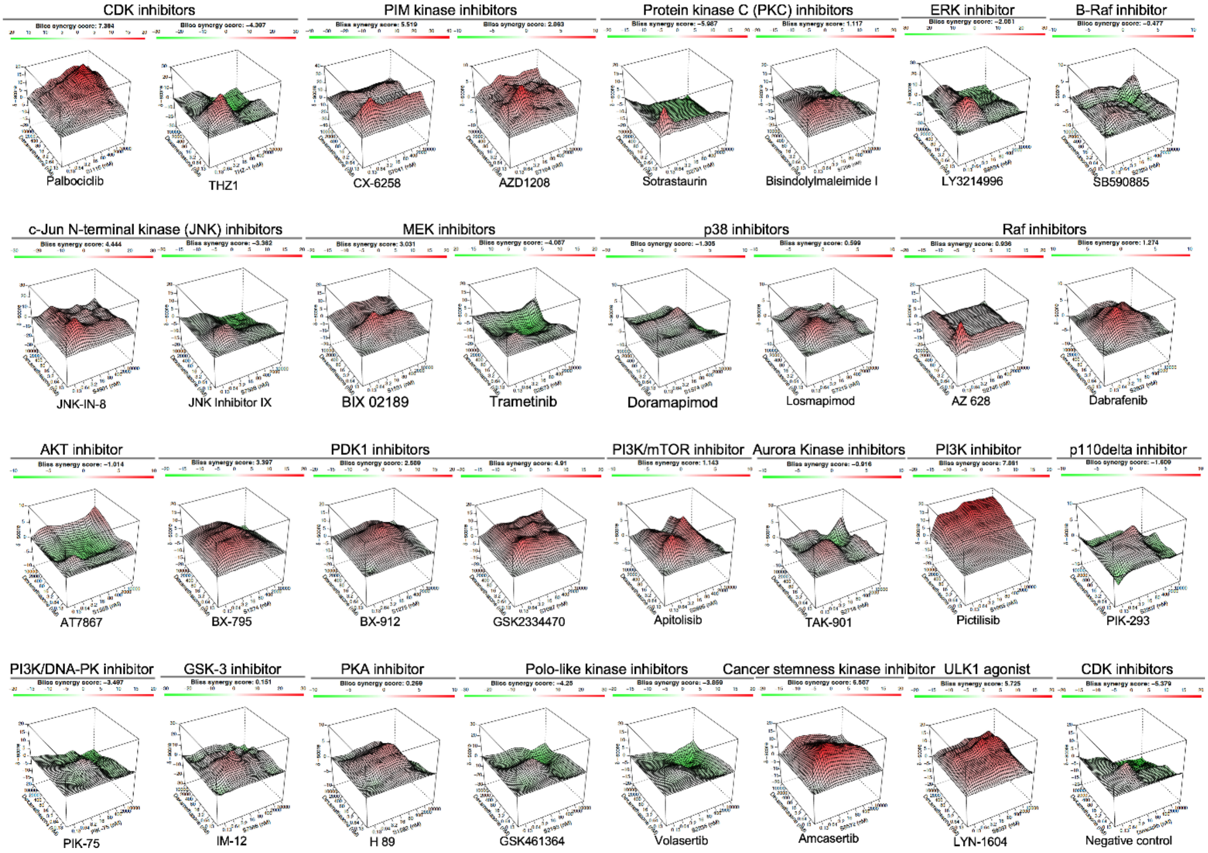

**Supplementary Figure 8. The combined analysis identifies Aurora kinase and downstream effector kinases stabilizing β-catenin expression.** Dexamethasone initiates Aurora kinase activation, which, in turn, results in the phosphorylation-dependent inhibition of GSK-3β. Aurora kinases can also directly or indirectly activate several other kinases such as JAK2, p38, and S6K, which inactivate GSK-3β through direct or indirect phosphorylation. GSK-3β inactivation stabilizes β-catenin protein, resulting in its accumulation.

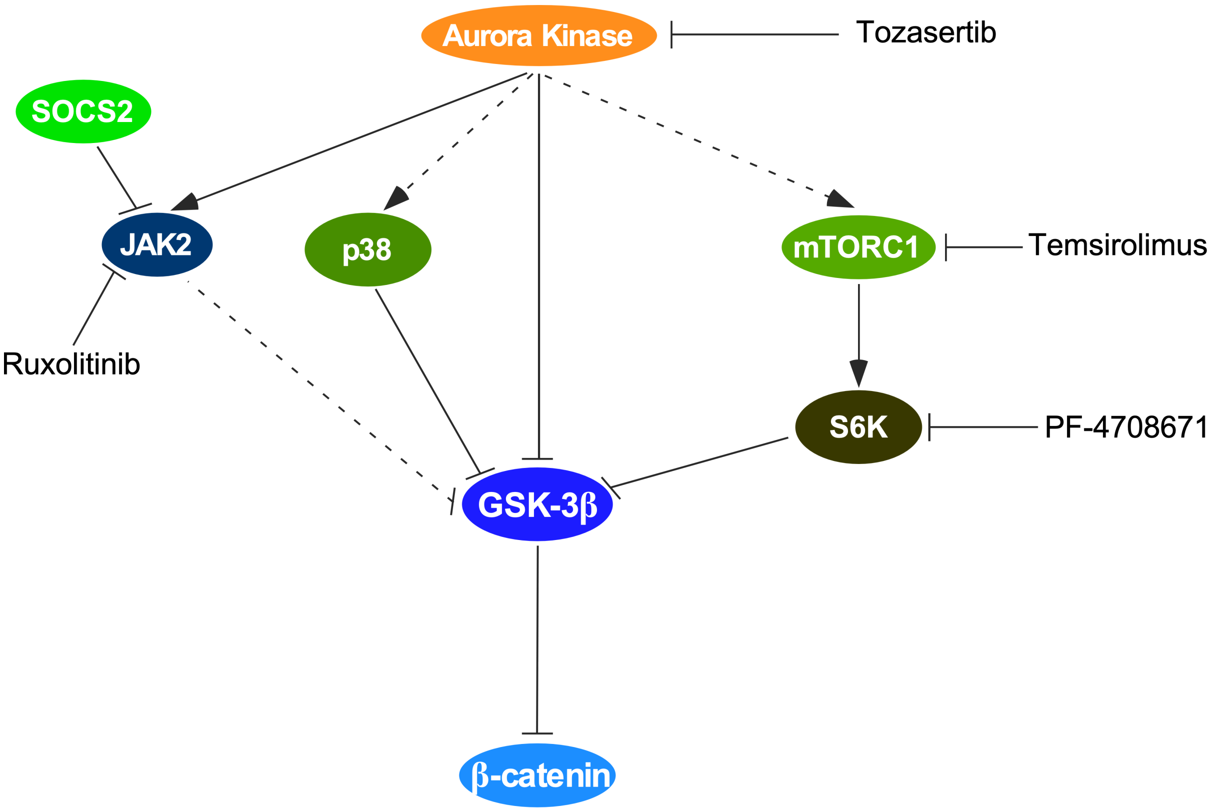

**Supplementary Table 1**

**Supplementary Table 1a. Genes upregulated in dexamethasone-treated SUP-B15 cells**

| Symbol | Fold Change | q-value(%) |
|---|---|---|
| OR7A5 | 25.721841 | 0 |
| CALCRL | 17.86492559 | 0 |
| PLEKHG1 | 8.76036714 | 0 |
| LILRA2 | 8.466832412 | 0 |
| LDB3 | 8.374126239 | 0 |
| SPRY1 | 7.663757971 | 0 |
| P2RY14 | 7.547840833 | 0 |
| OR7C1 | 6.945590398 | 0 |
| DDIT4 | 5.75876054 | 0 |
| DKK1 | 5.700733924 | 0 |
| TMEM236 | 5.611454103 | 0 |
| TP53INP1 | 5.208854 | 0 |
| TMEM217 | 4.915269998 | 0 |
| SYNE2 | 4.789330514 | 0 |
| CHRNA1 | 4.729522934 | 0 |
| LONRF1 | 4.660649434 | 0 |
| EPAS1 | 4.423582818 | 0 |
| EPHA4 | 4.348976762 | 0 |
| NGFR | 4.077117872 | 0 |
| GP9 | 3.977488245 | 0 |
| RANBP3L | 3.967801905 | 0 |
| OLAH | 3.955096787 | 0 |
| NFIL3 | 3.931494038 | 0 |
| NFKBIZ | 3.918577418 | 0 |
| SMIM3 | 3.890084967 | 0 |
| MYO10 | 3.685205318 | 0 |
| DPEP1 | 3.650509076 | 0 |
| DENND3 | 3.552610031 | 0 |
| ELL2 | 3.501233631 | 0 |
| TMEM204 | 3.423912896 | 0 |
| PON2 | 3.326580625 | 0 |
| RCAN1 | 3.293878485 | 0 |
| CTHRC1 | 3.282541688 | 0 |
| YBX3 | 3.280678759 | 0 |
| FBXW7 | 3.225276097 | 0 |
| BTG2 | 3.177778717 | 0 |
| EDNRB | 3.164929434 | 0 |
| INPP1 | 3.137682411 | 0 |
| SPTLC3 | 3.100765305 | 0 |

| | | |
|---|---|---|
| KLF7 | 3.100680767 | 0 |
| SLC22A23 | 3.074970706 | 0 |
| RASAL2 | 2.989538244 | 0 |
| TSC22D3 | 2.982797423 | 0 |
| TXNIP | 2.97697822 | 0 |
| GLIS3 | 2.942858822 | 0 |
| SLA | 2.862156051 | 0 |
| VPS37B | 2.834971699 | 0 |
| KLF9 | 2.794326628 | 0 |
| LDLRAD4 | 2.751616986 | 0 |
| KLF2 | 2.744151248 | 0 |
| BAALC | 2.716450328 | 0 |
| GADD45A | 2.709113805 | 0 |
| ITGA6 | 2.693446981 | 0 |
| SLC44A1 | 2.684034719 | 0 |
| SPRY4 | 2.682204059 | 0 |
| EPC1 | 2.65006914 | 0 |
| FAM43A | 2.640106266 | 0 |
| RASD1 | 2.615924676 | 0 |
| SMAP2 | 2.566171635 | 0 |
| CYTH3 | 2.532563893 | 0 |
| DUSP1 | 2.46741776 | 0 |
| TLR5 | 2.450862136 | 0 |
| SHISA2 | 2.450855341 | 0 |
| MERTK | 2.42265001 | 0 |
| FKBP5 | 2.384232068 | 0 |
| CPM | 2.365575707 | 0 |
| LGALSL | 2.352560786 | 0 |
| SAP30L | 2.343137039 | 0 |
| RNASET2 | 2.317489282 | 0 |
| ZC3H12D | 2.316156386 | 0 |
| DDR1 | 2.309944731 | 0 |
| ITGA10 | 2.309816644 | 0 |
| RAPGEF5 | 2.308403356 | 0 |
| IGFBP7 | 2.295550229 | 0 |
| SLC25A14 | 2.274424167 | 0 |
| SPTA1 | 2.250694638 | 0 |
| TLE1 | 2.246741795 | 0 |
| ITGA11 | 2.215911607 | 0 |
| TMSB4X | 2.194326696 | 0 |
| ABCC4 | 2.189684478 | 0 |
| CD200 | 2.164334771 | 0 |
| TNFRSF21 | 2.124989923 | 0 |

| | | |
|---|---|---|
| CHKA | 2.110493423 | 0 |
| UBASH3B | 2.108903381 | 0 |
| GSN | 2.081279422 | 0 |
| STYK1 | 2.797580542 | 0.412942551 |
| PLA2G10 | 2.755113334 | 0.412942551 |
| TFPI | 2.617174888 | 0.412942551 |
| TGFB1I1 | 2.599023479 | 0.412942551 |
| KCNK3 | 2.490894641 | 0.412942551 |
| FGD2 | 2.449616094 | 0.412942551 |
| MPV17L | 2.402940068 | 0.412942551 |
| ATP4B | 2.388436741 | 0.412942551 |
| ISG20 | 2.367505866 | 0.412942551 |
| ATMIN | 2.361201233 | 0.412942551 |
| OPN3 | 2.342081587 | 0.412942551 |
| BCL2L11 | 2.301880065 | 0.412942551 |
| TNFSF4 | 2.298270061 | 0.412942551 |
| CMTM2 | 2.285624385 | 0.412942551 |
| IRGM | 2.259479812 | 0.412942551 |
| FZD8 | 2.256561868 | 0.412942551 |
| NFKBIA | 2.255353641 | 0.412942551 |
| LMO2 | 2.232206248 | 0.412942551 |
| FNBP1L | 2.190487527 | 0.412942551 |
| MRC1 | 2.154700474 | 0.412942551 |
| BMP3 | 2.148222857 | 0.412942551 |
| ZIC2 | 2.143286432 | 0.412942551 |
| BTBD3 | 2.139791656 | 0.412942551 |
| HS3ST3B1 | 2.120835415 | 0.412942551 |
| CD69 | 2.116529113 | 0.412942551 |
| RECK | 2.116090506 | 0.412942551 |
| CD96 | 2.110629963 | 0.412942551 |
| TSC22D4 | 2.096585018 | 0.412942551 |
| F2RL3 | 2.075464133 | 0.412942551 |
| VAV3 | 2.062280157 | 0.412942551 |
| SGK1 | 2.05784169 | 0.412942551 |
| FZD4 | 2.054932018 | 0.412942551 |
| PLEKHF2 | 2.050436881 | 0.412942551 |
| AHDC1 | 2.030311843 | 0.412942551 |
| DUSP6 | 2.02077029 | 0.412942551 |
| CD53 | 2.006071926 | 0.412942551 |
| VWA2 | 2.003734462 | 0.412942551 |
| PREX1 | 1.994692004 | 0.412942551 |
| IRF8 | 1.976224253 | 0.412942551 |
| ASB13 | 1.962865519 | 0.412942551 |

| | | |
|---|---|---|
| F2R | 1.955028989 | 0.412942551 |
| AGPS | 1.946708224 | 0.412942551 |
| ARHGAP42 | 1.945867757 | 0.412942551 |
| RASA3 | 1.911278481 | 0.412942551 |
| MED13L | 1.900604478 | 0.412942551 |
| SLC9A9 | 1.88886335 | 0.412942551 |
| OR52N1 | 2.841740859 | 0.687025899 |
| GRAMD1C | 2.246139192 | 0.687025899 |
| CLYBL | 2.192232784 | 0.687025899 |
| NFATC1 | 2.181305743 | 0.687025899 |
| SOCS1 | 2.102132101 | 0.687025899 |
| IRAK3 | 2.089531248 | 0.687025899 |
| BIK | 2.036197262 | 0.687025899 |
| SULF2 | 2.002842072 | 0.687025899 |
| SMAD3 | 1.993870439 | 0.687025899 |
| IFNLR1 | 1.993822989 | 0.687025899 |
| ANKRD33B | 1.983821196 | 0.687025899 |
| PAQR4 | 1.915127354 | 0.687025899 |
| LINGO3 | 1.905499637 | 0.687025899 |
| TBC1D22B | 1.853463879 | 0.687025899 |
| FAM168A | 1.836949119 | 0.687025899 |
| INSR | 1.826159781 | 0.687025899 |
| SMOX | 1.794279947 | 0.687025899 |
| BMP2 | 2.267798827 | 0.880881965 |
| KCNA5 | 2.236571686 | 0.880881965 |
| LILRA1 | 2.11100842 | 0.880881965 |
| PTHLH | 2.05242049 | 0.880881965 |
| MUC4 | 2.040949285 | 0.880881965 |
| PLCH1 | 1.988484523 | 0.880881965 |
| CEACAM21 | 1.978176746 | 0.880881965 |
| RGL1 | 1.952793412 | 0.880881965 |
| RASSF4 | 1.952173123 | 0.880881965 |
| PIK3IP1 | 1.946822473 | 0.880881965 |
| SLC27A3 | 1.897668948 | 0.880881965 |
| METTL7A | 1.889162321 | 0.880881965 |
| TIPARP | 1.85605998 | 0.880881965 |
| TRAK2 | 1.839250521 | 0.880881965 |
| CCDC107 | 1.830386098 | 0.880881965 |
| CCDC186 | 1.81608786 | 0.880881965 |
| FZD6 | 1.809670869 | 0.880881965 |
| TAP1 | 1.728060028 | 0.880881965 |
| LRRFIP1 | 1.712890982 | 0.880881965 |
| IGF2BP2 | 2.264466715 | 1.227140599 |

| | | |
|---|---|---|
| STAG3 | 2.259733543 | 1.227140599 |
| F3 | 2.16127652 | 1.227140599 |
| MYLK | 1.832649213 | 1.227140599 |
| COCH | 1.828916657 | 1.227140599 |
| HECW1 | 1.813937817 | 1.227140599 |
| LAIR1 | 1.800710594 | 1.227140599 |
| SYNE3 | 1.790254467 | 1.227140599 |
| SLC16A2 | 1.770242086 | 1.227140599 |
| TTYH2 | 1.767631648 | 1.227140599 |
| AAK1 | 1.764817498 | 1.227140599 |
| KLF6 | 1.764126888 | 1.227140599 |
| CDK19 | 1.750458267 | 1.227140599 |
| SSH2 | 1.731717182 | 1.227140599 |
| TAPT1 | 1.723866835 | 1.227140599 |
| LIMD1 | 1.721545924 | 1.227140599 |
| IQSEC1 | 1.715891107 | 1.227140599 |
| PTPRE | 1.715114231 | 1.227140599 |
| WASF2 | 1.695232714 | 1.227140599 |
| MLXIP | 1.694785863 | 1.227140599 |
| CDON | 1.657921846 | 1.227140599 |
| QPCT | 2.063089392 | 1.620630273 |
| ARHGEF28 | 1.828502586 | 1.620630273 |
| MYRIP | 1.815102474 | 1.620630273 |
| DGKG | 1.772202342 | 1.620630273 |
| PIGV | 1.769127059 | 1.620630273 |
| SERTAD1 | 1.759390833 | 1.620630273 |
| FAM214B | 1.748316038 | 1.620630273 |
| CPQ | 1.74804501 | 1.620630273 |
| IL6ST | 1.74799291 | 1.620630273 |
| GCSAM | 1.747061829 | 1.620630273 |
| WDR91 | 1.737523712 | 1.620630273 |
| NCOA7 | 1.733577499 | 1.620630273 |
| GTF2IRD2B | 1.724308205 | 1.620630273 |
| OGFRL1 | 1.714379693 | 1.620630273 |
| SIK1 | 1.713793557 | 1.620630273 |
| CDH5 | 1.710248147 | 1.620630273 |
| SLC35E2B | 1.699649187 | 1.620630273 |
| UBALD2 | 1.698916955 | 1.620630273 |
| CYGB | 1.696724895 | 1.620630273 |
| CD72 | 1.686970018 | 1.620630273 |
| DCTN4 | 1.683726722 | 1.620630273 |
| NR3C1 | 1.682039206 | 1.620630273 |
| MGAT4A | 1.671902829 | 1.620630273 |

| | | |
|---|---|---|
| DISC1 | 1.647462165 | 1.620630273 |
| TMCC3 | 1.639202398 | 1.620630273 |
| PAG1 | 1.637441464 | 1.620630273 |
| SORT1 | 1.621974345 | 1.620630273 |
| DPYSL2 | 1.608967294 | 1.620630273 |
| APOLD1 | 1.604402738 | 1.620630273 |
| SERINC5 | 1.577527331 | 1.620630273 |
| TDRD1 | 2.029296023 | 1.943317594 |
| OR52N5 | 2.008232076 | 1.943317594 |
| GRB14 | 1.8751084 | 1.943317594 |
| SPAG4 | 1.789397617 | 1.943317594 |
| MDGA1 | 1.779568747 | 1.943317594 |
| SLC48A1 | 1.74333642 | 1.943317594 |
| DEPTOR | 1.732282232 | 1.943317594 |
| TBC1D10A | 1.727740245 | 1.943317594 |
| FBXO32 | 1.718319525 | 1.943317594 |
| FRMD3 | 1.698822357 | 1.943317594 |
| BEST3 | 1.688257929 | 1.943317594 |
| SMARCA2 | 1.673823027 | 1.943317594 |
| CAPN3 | 1.667911029 | 1.943317594 |
| PRDM8 | 1.66386648 | 1.943317594 |
| GLUL | 1.657568318 | 1.943317594 |
| KLHL42 | 1.64985816 | 1.943317594 |
| NT5DC2 | 1.630424158 | 1.943317594 |
| CTTNBP2NL | 1.622539575 | 1.943317594 |
| NR3C2 | 1.599889945 | 1.943317594 |
| AKNA | 1.598235494 | 1.943317594 |
| ADCY8 | 1.594572102 | 1.943317594 |
| ADPRM | 1.58559868 | 1.943317594 |
| ST8SIA4 | 1.584817446 | 1.943317594 |
| ETS2 | 1.546628461 | 1.943317594 |
| ATP11B | 1.532048533 | 1.943317594 |
| CPNE8 | 2.156587621 | 2.990461474 |
| SDC2 | 2.131278313 | 2.990461474 |
| THBS1 | 1.933084005 | 2.990461474 |
| NDRG1 | 1.80667875 | 2.990461474 |
| STK32B | 1.794081795 | 2.990461474 |
| MED12L | 1.756629577 | 2.990461474 |
| GSAP | 1.732930345 | 2.990461474 |
| USP12 | 1.714963257 | 2.990461474 |
| SERINC2 | 1.683005627 | 2.990461474 |
| TMEM65 | 1.665369915 | 2.990461474 |
| MAF | 1.642087116 | 2.990461474 |

| | | |
|---|---|---|
| PLXND1 | 1.633283239 | 2.990461474 |
| PRX | 1.63143631 | 2.990461474 |
| NEURL1B | 1.62759791 | 2.990461474 |
| CD58 | 1.626527258 | 2.990461474 |
| CHST15 | 1.616425108 | 2.990461474 |
| ERICH1 | 1.614147033 | 2.990461474 |
| WIPI1 | 1.606853424 | 2.990461474 |
| ADD3 | 1.59639242 | 2.990461474 |
| PER1 | 1.593480097 | 2.990461474 |
| CXCR4 | 1.570840464 | 2.990461474 |
| TRPM8 | 1.567519076 | 2.990461474 |
| DGKA | 1.560579298 | 2.990461474 |
| TRAM2 | 1.559943021 | 2.990461474 |
| THRA | 1.552279124 | 2.990461474 |
| CYFIP1 | 1.54986118 | 2.990461474 |
| PRR15 | 1.546000729 | 2.990461474 |
| PDE7A | 1.542257149 | 2.990461474 |
| RAB43 | 1.541576339 | 2.990461474 |
| NRP1 | 1.532460619 | 2.990461474 |
| NCK2 | 1.51587802 | 2.990461474 |
| SLC9A7 | 1.503346255 | 2.990461474 |
| TBL1Y | 1.945579141 | 4.206079513 |
| TMEM100 | 1.883097457 | 4.206079513 |
| NMRK1 | 1.838396555 | 4.206079513 |
| IFNW1 | 1.816668267 | 4.206079513 |
| CA6 | 1.741116413 | 4.206079513 |
| IFNA21 | 1.733549061 | 4.206079513 |
| ST6GALNAC3 | 1.676868627 | 4.206079513 |
| ZMAT1 | 1.670878311 | 4.206079513 |
| IL1RAP | 1.64878696 | 4.206079513 |
| ZBTB16 | 1.635891426 | 4.206079513 |
| IRS2 | 1.632816123 | 4.206079513 |
| TUBB4A | 1.626039533 | 4.206079513 |
| NT5E | 1.61626527 | 4.206079513 |
| PICK1 | 1.612020399 | 4.206079513 |
| MAP3K6 | 1.606424674 | 4.206079513 |
| LY96 | 1.59728306 | 4.206079513 |
| JUN | 1.585425039 | 4.206079513 |
| KLF10 | 1.576833866 | 4.206079513 |
| SP100 | 1.570149576 | 4.206079513 |
| ARMC12 | 1.557617862 | 4.206079513 |
| PTPRG | 1.550523795 | 4.206079513 |
| HIP1R | 1.544943773 | 4.206079513 |

| | | |
|---|---|---|
| WASL | 1.539849123 | 4.206079513 |
| KALRN | 1.539106432 | 4.206079513 |
| PSTPIP2 | 1.535739657 | 4.206079513 |
| MFSD6 | 1.530616298 | 4.206079513 |
| FAM171A1 | 1.52653641 | 4.206079513 |
| ABCB1 | 1.526356188 | 4.206079513 |
| MAP3K5 | 1.520612266 | 4.206079513 |
| YPEL3 | 1.520272562 | 4.206079513 |
| GPR3 | 1.519415033 | 4.206079513 |
| ENC1 | 1.518501851 | 4.206079513 |
| RAP1GAP2 | 1.515155992 | 4.206079513 |
| F2RL2 | 1.511753235 | 4.206079513 |
| PARP15 | 1.508883409 | 4.206079513 |
| EMILIN2 | 1.504882312 | 4.206079513 |
| RAPGEF3 | 1.502718036 | 4.206079513 |
| HIVEP3 | 1.502350742 | 4.206079513 |
| IFITM1 | 1.498049885 | 4.206079513 |
| SLC44A2 | 1.495284495 | 4.206079513 |
| KLHL6 | 1.488178244 | 4.206079513 |
| CHML | 1.480131477 | 4.206079513 |
| C20orf194 | 1.475305092 | 4.206079513 |
| ITPR1 | 1.4701408 | 4.206079513 |
| CDS2 | 1.469191716 | 4.206079513 |
| STRADB | 1.468107556 | 4.206079513 |
| RHOBTB3 | 1.460951211 | 4.206079513 |
| CAMK1D | 1.447439118 | 4.206079513 |

**Supplementary Table 1b. Genes downregulated in dexamethasone-treated SUP-B15 cells.**

| Symbol | Fold Change | q-value(%) |
|---|---|---|
| PUS7 | -3.176114669 | 0.412942551 |
| OSR2 | -2.901447183 | 0.412942551 |
| PTGER4 | -2.520584523 | 0.412942551 |
| POLR3G | -2.305806315 | 0.412942551 |
| GCSH | -3.213458112 | 0.687025899 |
| TLR10 | -2.681216406 | 0.687025899 |
| NOP16 | -2.433672999 | 0.687025899 |
| UGT3A2 | -2.4029595 | 0.687025899 |
| WDR3 | -2.35457063 | 0.687025899 |
| SLC8A1 | -2.331580111 | 0.687025899 |
| TAF4B | -2.329694852 | 0.687025899 |
| LYAR | -2.319418253 | 0.687025899 |
| MYC | -2.308297221 | 0.687025899 |
| PCDH18 | -2.296099243 | 0.687025899 |
| RPL22L1 | -2.281352426 | 0.687025899 |
| CHRNA5 | -2.265380935 | 0.687025899 |
| RPL12 | -2.259192702 | 0.687025899 |
| CD55 | -2.237297332 | 0.687025899 |
| HK2 | -2.224952186 | 0.687025899 |
| METTL1 | -2.220897792 | 0.687025899 |
| SIX3 | -2.211773012 | 0.687025899 |
| SLC25A19 | -2.209103991 | 0.687025899 |
| GBP6 | -2.183087062 | 0.687025899 |
| WDR12 | -2.173597784 | 0.687025899 |
| NOL6 | -2.133071512 | 0.687025899 |
| MARS2 | -2.132605825 | 0.687025899 |
| FAM216A | -2.108209637 | 0.687025899 |
| PNO1 | -2.102025737 | 0.687025899 |
| PLD6 | -2.101120641 | 0.687025899 |
| S1PR1 | -2.099787015 | 0.687025899 |
| RRS1 | -2.080926968 | 0.687025899 |
| MYBBP1A | -2.064823787 | 0.687025899 |
| DHX33 | -2.058486043 | 0.687025899 |
| CTSC | -2.047984316 | 0.687025899 |
| TFAP4 | -2.03332665 | 0.687025899 |
| SLC38A5 | -1.984536825 | 0.687025899 |
| MRTO4 | -1.974780996 | 0.687025899 |
| POLR1B | -1.952051344 | 0.687025899 |
| DCTPP1 | -1.947312379 | 0.687025899 |
| SLC39A14 | -1.872037066 | 0.687025899 |

| | | |
|---|---|---|
| IL1B | -2.851961817 | 0.880881965 |
| MAP2K6 | -2.511529116 | 0.880881965 |
| SAMHD1 | -2.072470165 | 0.880881965 |
| CLUH | -2.026717956 | 0.880881965 |
| HBEGF | -2.011017078 | 0.880881965 |
| CD244 | -1.988198774 | 0.880881965 |
| SFXN4 | -1.968565433 | 0.880881965 |
| PPARGC1B | -1.942256402 | 0.880881965 |
| LIMA1 | -1.931050643 | 0.880881965 |
| PDE3B | -1.919164443 | 0.880881965 |
| PPAT | -1.917586955 | 0.880881965 |
| RPS2 | -1.912453937 | 0.880881965 |
| NOC3L | -1.905421712 | 0.880881965 |
| DPH2 | -1.886442335 | 0.880881965 |
| GPATCH4 | -1.883792856 | 0.880881965 |
| FJX1 | -1.872886319 | 0.880881965 |
| PAK1IP1 | -1.87127726 | 0.880881965 |
| RABEPK | -1.861635551 | 0.880881965 |
| IL7R | -1.857252541 | 0.880881965 |
| MUC19 | -1.854004827 | 0.880881965 |
| RRP12 | -1.839718458 | 0.880881965 |
| CAMKK2 | -1.835935023 | 0.880881965 |
| ACACA | -1.822120874 | 0.880881965 |
| DDX21 | -1.820737996 | 0.880881965 |
| NR1D1 | -1.798035701 | 0.880881965 |
| OAS2 | -1.797749489 | 0.880881965 |
| POLR1A | -1.790247435 | 0.880881965 |
| XPO5 | -1.784905466 | 0.880881965 |
| SCARB1 | -1.78280552 | 0.880881965 |
| NIFK | -1.778589204 | 0.880881965 |
| DKC1 | -1.77783734 | 0.880881965 |
| TIMM8A | -1.774238965 | 0.880881965 |
| NAA25 | -1.763441846 | 0.880881965 |
| DHX37 | -1.749477771 | 0.880881965 |
| MLKL | -1.749064711 | 0.880881965 |
| COA7 | -1.74331628 | 0.880881965 |
| LGR5 | -1.733181008 | 0.880881965 |
| RPS18 | -2.051515423 | 1.227140599 |
| FDXACB1 | -1.987806968 | 1.227140599 |
| ALDH1B1 | -1.94971646 | 1.227140599 |
| PWP2 | -1.909802344 | 1.227140599 |
| FKBP14 | -1.908144375 | 1.227140599 |
| IFIT1 | -1.902375886 | 1.227140599 |

| | | |
|---|---|---|
| RPS27A | -1.896886906 | 1.227140599 |
| ITGB7 | -1.890335095 | 1.227140599 |
| MRM1 | -1.884019199 | 1.227140599 |
| GPR174 | -1.878508095 | 1.227140599 |
| PPRC1 | -1.856331457 | 1.227140599 |
| TAF1D | -1.847938401 | 1.227140599 |
| HSPA9 | -1.847800923 | 1.227140599 |
| NLN | -1.841819905 | 1.227140599 |
| RCL1 | -1.820324935 | 1.227140599 |
| RPF2 | -1.813958772 | 1.227140599 |
| KCNMB4 | -1.804775431 | 1.227140599 |
| KLHL14 | -1.795197618 | 1.227140599 |
| MCTP1 | -1.79243069 | 1.227140599 |
| AMPD2 | -1.789492297 | 1.227140599 |
| SLC27A2 | -1.785908294 | 1.227140599 |
| TTLL12 | -1.781592018 | 1.227140599 |
| ESF1 | -1.770026958 | 1.227140599 |
| WDR43 | -1.766852574 | 1.227140599 |
| PIK3C2B | -1.766747662 | 1.227140599 |
| NT5DC3 | -1.764377172 | 1.227140599 |
| RPP40 | -1.761237743 | 1.227140599 |
| ROBO1 | -1.749988369 | 1.227140599 |
| ZNF749 | -1.745826269 | 1.227140599 |
| POLR1C | -1.742299528 | 1.227140599 |
| BYSL | -1.721897581 | 1.227140599 |
| GNL3 | -1.717770936 | 1.227140599 |
| NOP2 | -1.712747327 | 1.227140599 |
| TSEN2 | -1.708972284 | 1.227140599 |
| EEF2K | -1.703202139 | 1.227140599 |
| NIP7 | -1.694243222 | 1.227140599 |
| RPS28 | -1.689654959 | 1.227140599 |
| TSR1 | -1.680090949 | 1.227140599 |
| PPIF | -1.673899602 | 1.227140599 |
| UTP20 | -1.67337834 | 1.227140599 |
| NLE1 | -1.665263333 | 1.227140599 |
| ABHD17C | -1.662238043 | 1.227140599 |
| PDCD11 | -1.658133692 | 1.227140599 |
| RPS25 | -1.647500611 | 1.227140599 |
| PFDN2 | -1.646896244 | 1.227140599 |
| TMEM120B | -1.639946405 | 1.227140599 |
| EARS2 | -1.636290612 | 1.227140599 |
| GTPBP4 | -1.635001546 | 1.227140599 |
| HEATR3 | -1.610856894 | 1.227140599 |

| | | |
|---|---|---|
| PTPN22 | -2.155413501 | 1.620630273 |
| FKBP7 | -2.002659755 | 1.620630273 |
| FZD3 | -1.896184045 | 1.620630273 |
| FRMPD1 | -1.858633522 | 1.620630273 |
| NDUFAF4 | -1.856007234 | 1.620630273 |
| RPL32 | -1.83858091 | 1.620630273 |
| GNPNAT1 | -1.8170255 | 1.620630273 |
| RGS13 | -1.816795033 | 1.620630273 |
| LGI2 | -1.791836084 | 1.620630273 |
| TRAF3 | -1.774623117 | 1.620630273 |
| MYOCD | -1.767757851 | 1.620630273 |
| E2F5 | -1.767608777 | 1.620630273 |
| CCNJ | -1.764834216 | 1.620630273 |
| TRIB2 | -1.761167752 | 1.620630273 |
| ELOVL6 | -1.761059109 | 1.620630273 |
| FCHO1 | -1.749175848 | 1.620630273 |
| CLEC2D | -1.74416396 | 1.620630273 |
| NAF1 | -1.735797519 | 1.620630273 |
| TBC1D30 | -1.729499184 | 1.620630273 |
| NOG | -1.724795915 | 1.620630273 |
| URB2 | -1.723356691 | 1.620630273 |
| NLRP3 | -1.722402518 | 1.620630273 |
| CCDC86 | -1.720954952 | 1.620630273 |
| KCNJ2 | -1.717877306 | 1.620630273 |
| YME1L1 | -1.715023882 | 1.620630273 |
| KLF3 | -1.714106798 | 1.620630273 |
| ARMC10 | -1.713363587 | 1.620630273 |
| CD48 | -1.713313708 | 1.620630273 |
| PDP2 | -1.711899095 | 1.620630273 |
| NUFIP1 | -1.710700655 | 1.620630273 |
| SORD | -1.707386487 | 1.620630273 |
| IPO4 | -1.707283529 | 1.620630273 |
| HMGA2 | -1.707270906 | 1.620630273 |
| FGD6 | -1.704943976 | 1.620630273 |
| KBTBD6 | -1.704231513 | 1.620630273 |
| POLR3H | -1.702152936 | 1.620630273 |
| PDGFRB | -1.702135238 | 1.620630273 |
| SLC18B1 | -1.698517795 | 1.620630273 |
| PM20D2 | -1.696319979 | 1.620630273 |
| ATP2B1 | -1.695150462 | 1.620630273 |
| PDSS1 | -1.694026762 | 1.620630273 |
| ABCE1 | -1.688836893 | 1.620630273 |
| PRICKLE2 | -1.685845898 | 1.620630273 |

| | | |
|---|---|---|
| TRIM25 | -1.682983073 | 1.620630273 |
| RPL41 | -1.682455873 | 1.620630273 |
| ZNRF3 | -1.678396607 | 1.620630273 |
| GPR155 | -1.676173706 | 1.620630273 |
| FAM162A | -1.675895663 | 1.620630273 |
| LPCAT4 | -1.675519333 | 1.620630273 |
| SLC16A1 | -1.67285067 | 1.620630273 |
| SLC25A33 | -1.672017947 | 1.620630273 |
| PTRH2 | -1.671832525 | 1.620630273 |
| SLC7A6 | -1.67124549 | 1.620630273 |
| RRP15 | -1.66879183 | 1.620630273 |
| RIOK1 | -1.668334218 | 1.620630273 |
| CBX6 | -1.660752401 | 1.620630273 |
| FAM117B | -1.660050734 | 1.620630273 |
| DDX31 | -1.657679003 | 1.620630273 |
| PRMT5 | -1.657591297 | 1.620630273 |
| NKRF | -1.654551655 | 1.620630273 |
| HTRA3 | -1.653666143 | 1.620630273 |
| TOMM40 | -1.652690604 | 1.620630273 |
| NOLC1 | -1.649047551 | 1.620630273 |
| SLC9B2 | -1.645475258 | 1.620630273 |
| UTP18 | -1.642315152 | 1.620630273 |
| MRPS25 | -1.64039623 | 1.620630273 |
| UBQLN4 | -1.634945638 | 1.620630273 |
| RITA1 | -1.633124751 | 1.620630273 |
| RPL13A | -1.631421232 | 1.620630273 |
| EXOSC5 | -1.631371854 | 1.620630273 |
| YRDC | -1.625901659 | 1.620630273 |
| ZNF202 | -1.623321026 | 1.620630273 |
| PFKM | -1.619577681 | 1.620630273 |
| SCD | -1.617957075 | 1.620630273 |
| RPS3A | -1.613884127 | 1.620630273 |
| SCFD2 | -1.610085536 | 1.620630273 |
| PNPT1 | -1.6098802 | 1.620630273 |
| MAK16 | -1.609429818 | 1.620630273 |
| AIMP2 | -1.609392632 | 1.620630273 |
| FXN | -1.603888295 | 1.620630273 |
| TRIAP1 | -1.600761085 | 1.620630273 |
| RRP1B | -1.593497033 | 1.620630273 |
| ITPRIPL1 | -1.592905484 | 1.620630273 |
| POLR1E | -1.591290611 | 1.620630273 |
| SLC25A37 | -1.588648551 | 1.620630273 |
| ALDH18A1 | -1.588284107 | 1.620630273 |

| | | |
|---|---|---|
| AHSA1 | -1.587982118 | 1.620630273 |
| ODC1 | -1.587287358 | 1.620630273 |
| UTP15 | -1.584947442 | 1.620630273 |
| WDR36 | -1.581799416 | 1.620630273 |
| DCUN1D5 | -1.580880883 | 1.620630273 |
| GEMIN5 | -1.576768653 | 1.620630273 |
| UTP3 | -1.574296921 | 1.620630273 |
| COA4 | -1.569623993 | 1.620630273 |
| NDUFAF2 | -1.567525233 | 1.620630273 |
| SACS | -1.566780415 | 1.620630273 |
| AP1AR | -1.563121946 | 1.620630273 |
| L3MBTL3 | -1.562509903 | 1.620630273 |
| SLC25A12 | -1.558728867 | 1.620630273 |
| TIMM17A | -1.539341507 | 1.620630273 |
| EIF3B | -1.52039375 | 1.620630273 |
| OR4N2 | -1.934382816 | 1.943317594 |
| GBP2 | -1.844475552 | 1.943317594 |
| PDE4DIP | -1.808901687 | 1.943317594 |
| ABCA9 | -1.783161863 | 1.943317594 |
| PDPN | -1.777269339 | 1.943317594 |
| HTR7 | -1.726948428 | 1.943317594 |
| NR1D2 | -1.716125031 | 1.943317594 |
| PIP5K1B | -1.711743262 | 1.943317594 |
| SLFN12L | -1.702638313 | 1.943317594 |
| PACSIN3 | -1.699741082 | 1.943317594 |
| RABGGTB | -1.685099756 | 1.943317594 |
| EXTL2 | -1.684826461 | 1.943317594 |
| UTP14A | -1.682671243 | 1.943317594 |
| PLXDC2 | -1.678189539 | 1.943317594 |
| NARS2 | -1.669220642 | 1.943317594 |
| TRIB1 | -1.666766104 | 1.943317594 |
| CCDC141 | -1.654547832 | 1.943317594 |
| RPS26 | -1.654441561 | 1.943317594 |
| SLC29A1 | -1.652977018 | 1.943317594 |
| ADAT2 | -1.649443469 | 1.943317594 |
| BOP1 | -1.635417141 | 1.943317594 |
| SRFBP1 | -1.631734875 | 1.943317594 |
| EIF2B3 | -1.630366146 | 1.943317594 |
| FAM118A | -1.630360872 | 1.943317594 |
| BZW2 | -1.629885555 | 1.943317594 |
| WDR4 | -1.629658114 | 1.943317594 |
| WDR46 | -1.619493113 | 1.943317594 |
| REXO2 | -1.618977572 | 1.943317594 |

| | | |
|---|---|---|
| DBP | -1.615507371 | 1.943317594 |
| LTV1 | -1.612523665 | 1.943317594 |
| PAWR | -1.612179073 | 1.943317594 |
| ATAD3A | -1.610549869 | 1.943317594 |
| LYSMD2 | -1.606384217 | 1.943317594 |
| ZNF286A | -1.602727698 | 1.943317594 |
| CISD1 | -1.601318552 | 1.943317594 |
| DIMT1 | -1.60057321 | 1.943317594 |
| GLDC | -1.599908797 | 1.943317594 |
| POLR3E | -1.591103848 | 1.943317594 |
| UBE2G2 | -1.590454758 | 1.943317594 |
| DHODH | -1.59002377 | 1.943317594 |
| RANGRF | -1.589752672 | 1.943317594 |
| BLNK | -1.588479715 | 1.943317594 |
| ZNF485 | -1.588420259 | 1.943317594 |
| C12orf45 | -1.587995693 | 1.943317594 |
| MAMLD1 | -1.585830231 | 1.943317594 |
| DUSP14 | -1.576388722 | 1.943317594 |
| NXT1 | -1.5758352 | 1.943317594 |
| FARSA | -1.566354396 | 1.943317594 |
| POLRMT | -1.565742173 | 1.943317594 |
| PFAS | -1.559417972 | 1.943317594 |
| TIMM44 | -1.558999357 | 1.943317594 |
| ARMC6 | -1.558004428 | 1.943317594 |
| SLC25A17 | -1.557923795 | 1.943317594 |
| CLEC2B | -1.557499104 | 1.943317594 |
| PMM2 | -1.554095327 | 1.943317594 |
| FAM98A | -1.551954577 | 1.943317594 |
| THUMPD2 | -1.551014672 | 1.943317594 |
| AMMECR1 | -1.550457163 | 1.943317594 |
| NOP56 | -1.549076437 | 1.943317594 |
| RRN3 | -1.548940436 | 1.943317594 |
| TRMT10C | -1.547866449 | 1.943317594 |
| DDX10 | -1.547681564 | 1.943317594 |
| PRDX4 | -1.547660108 | 1.943317594 |
| IFRD2 | -1.547010512 | 1.943317594 |
| ZNF57 | -1.544605771 | 1.943317594 |
| WDR77 | -1.544445897 | 1.943317594 |
| ELL3 | -1.542988169 | 1.943317594 |
| SNTB1 | -1.542768577 | 1.943317594 |
| TNPO2 | -1.541263288 | 1.943317594 |
| CYP51A1 | -1.539956217 | 1.943317594 |
| GCH1 | -1.539209206 | 1.943317594 |

| | | |
|---|---|---|
| TEX2 | -1.539101098 | 1.943317594 |
| PNP | -1.538100742 | 1.943317594 |
| CAD | -1.533863746 | 1.943317594 |
| MLC1 | -1.532186944 | 1.943317594 |
| MDN1 | -1.528838131 | 1.943317594 |
| MPHOSPH10 | -1.528244809 | 1.943317594 |
| LIAS | -1.526205256 | 1.943317594 |
| TOMM5 | -1.525715534 | 1.943317594 |
| FTSJ1 | -1.520125392 | 1.943317594 |
| DDX55 | -1.517092796 | 1.943317594 |
| NOP14 | -1.515382858 | 1.943317594 |
| KNOP1 | -1.514242221 | 1.943317594 |
| GNL2 | -1.511670107 | 1.943317594 |
| OGFOD1 | -1.51102235 | 1.943317594 |
| FASN | -1.510889341 | 1.943317594 |
| SRM | -1.49436579 | 1.943317594 |
| AMER1 | -1.492408001 | 1.943317594 |
| RPL9 | -1.492252496 | 1.943317594 |
| CD22 | -1.490476195 | 1.943317594 |
| ABHD4 | -1.484385349 | 1.943317594 |
| ISOC2 | -1.484147693 | 1.943317594 |
| TFRC | -1.475649749 | 1.943317594 |
| HSPE1-MOB4 | -1.474705968 | 1.943317594 |
| CCT3 | -1.474548559 | 1.943317594 |
| HSPH1 | -1.47031982 | 1.943317594 |
| SEH1L | -1.465723866 | 1.943317594 |
| AIF1 | -2.084059788 | 2.990461474 |
| TLR6 | -1.682680963 | 2.990461474 |
| L3HYPDH | -1.681890366 | 2.990461474 |
| EFNA4 | -1.671905919 | 2.990461474 |
| SMYD5 | -1.667633586 | 2.990461474 |
| PIGW | -1.647837523 | 2.990461474 |
| TTC27 | -1.624030057 | 2.990461474 |
| GPR18 | -1.620731756 | 2.990461474 |
| LOC102723899 | -1.61719091 | 2.990461474 |
| PDE3A | -1.61389233 | 2.990461474 |
| DESI1 | -1.6087989 | 2.990461474 |
| MEX3A | -1.605039349 | 2.990461474 |
| MYLIP | -1.602052397 | 2.990461474 |
| GPR65 | -1.599349973 | 2.990461474 |
| PPP2R3A | -1.598454487 | 2.990461474 |
| SLC10A5 | -1.594914036 | 2.990461474 |
| SRPRB | -1.592541167 | 2.990461474 |

| | | |
|---|---|---|
| NKIRAS1 | -1.590310715 | 2.990461474 |
| PLAUR | -1.590133251 | 2.990461474 |
| GBP3 | -1.571416557 | 2.990461474 |
| SLC35F2 | -1.56295835 | 2.990461474 |
| DGKE | -1.561841804 | 2.990461474 |
| WDR89 | -1.561292669 | 2.990461474 |
| BRIX1 | -1.560886173 | 2.990461474 |
| ALDH3A2 | -1.559747683 | 2.990461474 |
| ZNF814 | -1.559587323 | 2.990461474 |
| EXOSC7 | -1.559294754 | 2.990461474 |
| MRPL15 | -1.556980634 | 2.990461474 |
| GEMIN4 | -1.556924156 | 2.990461474 |
| RPIA | -1.555684315 | 2.990461474 |
| CD34 | -1.552629209 | 2.990461474 |
| C20orf96 | -1.551987208 | 2.990461474 |
| TMEM201 | -1.549227483 | 2.990461474 |
| ACTR5 | -1.547733057 | 2.990461474 |
| GABBR2 | -1.546775695 | 2.990461474 |
| DISP1 | -1.542799945 | 2.990461474 |
| SURF2 | -1.542384366 | 2.990461474 |
| STAMBPL1 | -1.541890165 | 2.990461474 |
| SLC25A15 | -1.541886246 | 2.990461474 |
| GSTM2 | -1.538485664 | 2.990461474 |
| FASTKD2 | -1.536753389 | 2.990461474 |
| PES1 | -1.536082107 | 2.990461474 |
| WDR74 | -1.534709927 | 2.990461474 |
| MRPL17 | -1.534048399 | 2.990461474 |
| SLC17A9 | -1.53325324 | 2.990461474 |
| IFIT5 | -1.533047785 | 2.990461474 |
| NANS | -1.532429107 | 2.990461474 |
| SPRYD7 | -1.532230488 | 2.990461474 |
| NME1 | -1.52957092 | 2.990461474 |
| POLR3D | -1.529444406 | 2.990461474 |
| RPL37 | -1.527106134 | 2.990461474 |
| ADARB1 | -1.52623523 | 2.990461474 |
| MRPL46 | -1.51879203 | 2.990461474 |
| AKR1A1 | -1.516025829 | 2.990461474 |
| BAG2 | -1.515127636 | 2.990461474 |
| ARHGEF3 | -1.514703412 | 2.990461474 |
| ATIC | -1.512744494 | 2.990461474 |
| SELL | -1.510113863 | 2.990461474 |
| EIF2AK3 | -1.51007653 | 2.990461474 |
| RRP1 | -1.507708299 | 2.990461474 |

| | | |
|---|---|---|
| DHCR7 | -1.505847493 | 2.990461474 |
| TTC39C | -1.503815941 | 2.990461474 |
| BAG1 | -1.499111475 | 2.990461474 |
| SLIRP | -1.498623175 | 2.990461474 |
| TRUB2 | -1.494622349 | 2.990461474 |
| ZNF330 | -1.49192223 | 2.990461474 |
| MTHFD1L | -1.491491063 | 2.990461474 |
| CHCHD4 | -1.49115063 | 2.990461474 |
| THNSL1 | -1.489772118 | 2.990461474 |
| EXOSC2 | -1.488753603 | 2.990461474 |
| DLAT | -1.487507903 | 2.990461474 |
| DNAJA1 | -1.484084598 | 2.990461474 |
| AFMID | -1.483235144 | 2.990461474 |
| TFB2M | -1.481456912 | 2.990461474 |
| TNFAIP8 | -1.481451093 | 2.990461474 |
| GRWD1 | -1.481192346 | 2.990461474 |
| EIF1AX | -1.48012498 | 2.990461474 |
| PINX1 | -1.478900167 | 2.990461474 |
| NOL11 | -1.477676026 | 2.990461474 |
| ZMYND19 | -1.477402236 | 2.990461474 |
| GPN3 | -1.475047418 | 2.990461474 |
| SLC25A4 | -1.472929794 | 2.990461474 |
| GART | -1.472314965 | 2.990461474 |
| WT1 | -1.471736438 | 2.990461474 |
| IDH3A | -1.467764999 | 2.990461474 |
| MRPS12 | -1.464651064 | 2.990461474 |
| ISOC1 | -1.464344838 | 2.990461474 |
| RPL21 | -1.463948362 | 2.990461474 |
| PRMT3 | -1.463517503 | 2.990461474 |
| ZC3H8 | -1.461844645 | 2.990461474 |
| RUVBL1 | -1.460748695 | 2.990461474 |
| PPIL1 | -1.458701815 | 2.990461474 |
| METTL16 | -1.458622614 | 2.990461474 |
| TANGO6 | -1.458617559 | 2.990461474 |
| LYRM4 | -1.457881036 | 2.990461474 |
| AMD1 | -1.457295386 | 2.990461474 |
| EXOSC4 | -1.456687757 | 2.990461474 |
| HEATR1 | -1.454485923 | 2.990461474 |
| CMSS1 | -1.454132433 | 2.990461474 |
| NOL8 | -1.453988307 | 2.990461474 |
| AKAP1 | -1.453446532 | 2.990461474 |
| RFTN1 | -1.452643143 | 2.990461474 |
| IKZF2 | -1.451698982 | 2.990461474 |

| | | |
|---|---|---|
| SUPV3L1 | -1.45121942 | 2.990461474 |
| ZPR1 | -1.450990091 | 2.990461474 |
| DDX18 | -1.447085335 | 2.990461474 |
| LDHA | -1.446922182 | 2.990461474 |
| POLR3B | -1.442819007 | 2.990461474 |
| NOL10 | -1.44261467 | 2.990461474 |
| DUSP7 | -1.440300324 | 2.990461474 |
| C12orf66 | -1.440249743 | 2.990461474 |
| MILR1 | -1.437914273 | 2.990461474 |
| FTSJ3 | -1.434851733 | 2.990461474 |
| HAUS7 | -1.433819416 | 2.990461474 |
| NCL | -1.433757798 | 2.990461474 |
| MRPL3 | -1.432369132 | 2.990461474 |
| NUDCD1 | -1.432318829 | 2.990461474 |
| NCLN | -1.430685599 | 2.990461474 |
| TJP2 | -1.426068221 | 2.990461474 |
| REP15 | -1.869280837 | 4.206079513 |
| ZDHHC11 | -1.852756132 | 4.206079513 |
| NELL2 | -1.710927151 | 4.206079513 |
| NAMPT | -1.643960916 | 4.206079513 |
| FASTKD3 | -1.610766828 | 4.206079513 |
| CALML4 | -1.580926176 | 4.206079513 |
| TMEM192 | -1.58015418 | 4.206079513 |
| APOO | -1.559247198 | 4.206079513 |
| TEX12 | -1.554581587 | 4.206079513 |
| SLC4A4 | -1.551159814 | 4.206079513 |
| TAF1A | -1.550946226 | 4.206079513 |
| PUS1 | -1.549480215 | 4.206079513 |
| PROM1 | -1.546289376 | 4.206079513 |
| TMEM117 | -1.545959652 | 4.206079513 |
| B4GALT5 | -1.538034288 | 4.206079513 |
| UCHL3 | -1.533949867 | 4.206079513 |
| FAIM | -1.523896583 | 4.206079513 |
| UBE3D | -1.51523161 | 4.206079513 |
| C1orf21 | -1.515040822 | 4.206079513 |
| HNRNPH2 | -1.512428213 | 4.206079513 |
| CCDC58 | -1.512283899 | 4.206079513 |
| C15orf61 | -1.511880731 | 4.206079513 |
| E2F6 | -1.50729869 | 4.206079513 |
| DNAJC2 | -1.506031557 | 4.206079513 |
| NELL1 | -1.503809687 | 4.206079513 |
| CITED4 | -1.502022752 | 4.206079513 |
| RAB38 | -1.499589193 | 4.206079513 |

| | | |
|---|---|---|
| RPS17 | -1.498251689 | 4.206079513 |
| GDF11 | -1.49583357 | 4.206079513 |
| WDR75 | -1.493818979 | 4.206079513 |
| SLC12A9 | -1.489208752 | 4.206079513 |
| POLR2H | -1.488463661 | 4.206079513 |
| SPATA5L1 | -1.488373559 | 4.206079513 |
| B3GALNT2 | -1.48706633 | 4.206079513 |
| PLCG2 | -1.485570769 | 4.206079513 |
| FKBP11 | -1.484923217 | 4.206079513 |
| RPLP0 | -1.483376686 | 4.206079513 |
| MMACHC | -1.479768336 | 4.206079513 |
| ASPH | -1.479322908 | 4.206079513 |
| TOB1 | -1.478923403 | 4.206079513 |
| GRPEL1 | -1.474565594 | 4.206079513 |
| FAM210A | -1.474426937 | 4.206079513 |
| POP1 | -1.474328488 | 4.206079513 |
| ZNF331 | -1.47358948 | 4.206079513 |
| METTL8 | -1.473031893 | 4.206079513 |
| GAR1 | -1.470916482 | 4.206079513 |
| RSC1A1 | -1.468889292 | 4.206079513 |
| NDST1 | -1.467948478 | 4.206079513 |
| NGRN | -1.467690733 | 4.206079513 |
| EIF4EBP1 | -1.466630046 | 4.206079513 |
| PPTC7 | -1.465405565 | 4.206079513 |
| DNPEP | -1.465118477 | 4.206079513 |
| SNRPF | -1.462143254 | 4.206079513 |
| DFFB | -1.461121685 | 4.206079513 |
| PER2 | -1.461101429 | 4.206079513 |
| KLLN | -1.459807023 | 4.206079513 |
| PUS7L | -1.454906728 | 4.206079513 |
| RAB9A | -1.453727638 | 4.206079513 |
| INTS5 | -1.453199391 | 4.206079513 |
| EMC1 | -1.451814704 | 4.206079513 |
| FUT11 | -1.451806318 | 4.206079513 |
| MRPS17 | -1.450288582 | 4.206079513 |
| MIEF1 | -1.44741437 | 4.206079513 |
| TBC1D8 | -1.444990158 | 4.206079513 |
| ZNF143 | -1.443227099 | 4.206079513 |
| MRPL12 | -1.440518312 | 4.206079513 |
| FARSB | -1.439681819 | 4.206079513 |
| SEC11C | -1.439673503 | 4.206079513 |
| RBM28 | -1.438889695 | 4.206079513 |
| CCT2 | -1.438783979 | 4.206079513 |

| | | |
|---|---|---|
| MBLAC2 | -1.437899655 | 4.206079513 |
| HILPDA | -1.435373973 | 4.206079513 |
| TBL2 | -1.43502745 | 4.206079513 |
| FLAD1 | -1.434711174 | 4.206079513 |
| CRIM1 | -1.43461372 | 4.206079513 |
| CYTIP | -1.43150165 | 4.206079513 |
| EIF3J | -1.430832705 | 4.206079513 |
| TRMT61B | -1.430249328 | 4.206079513 |
| TRAPPC2L | -1.429999524 | 4.206079513 |
| CENPV | -1.429503019 | 4.206079513 |
| NAT10 | -1.427174094 | 4.206079513 |
| CARM1 | -1.426710545 | 4.206079513 |
| HIVEP2 | -1.426328873 | 4.206079513 |
| EPHA7 | -1.42597992 | 4.206079513 |
| MRPL4 | -1.424935549 | 4.206079513 |
| IMPDH1 | -1.42388964 | 4.206079513 |
| TRIT1 | -1.42225222 | 4.206079513 |
| BCL2 | -1.420544802 | 4.206079513 |
| RDH10 | -1.419547706 | 4.206079513 |
| RPL7A | -1.418551964 | 4.206079513 |
| ISG20L2 | -1.418226213 | 4.206079513 |
| POFUT1 | -1.417805534 | 4.206079513 |
| TMC8 | -1.415330517 | 4.206079513 |
| TMEM177 | -1.414880622 | 4.206079513 |
| ABCF2 | -1.414457014 | 4.206079513 |
| PTCH1 | -1.414188403 | 4.206079513 |
| NAA15 | -1.414112273 | 4.206079513 |
| GNL3L | -1.411938907 | 4.206079513 |
| PTPRK | -1.410952415 | 4.206079513 |
| CEBPZ | -1.409437007 | 4.206079513 |
| NFS1 | -1.406678531 | 4.206079513 |
| PRPS1 | -1.406375328 | 4.206079513 |
| PRMT1 | -1.406256404 | 4.206079513 |
| LRRC59 | -1.404685348 | 4.206079513 |
| B4GALT3 | -1.403565443 | 4.206079513 |
| ALG3 | -1.401040506 | 4.206079513 |
| DLG3 | -1.400173558 | 4.206079513 |
| SLC16A7 | -1.398072329 | 4.206079513 |
| PWP1 | -1.397623399 | 4.206079513 |
| ICAM2 | -1.395104638 | 4.206079513 |
| TRMT5 | -1.391971494 | 4.206079513 |
| MRPS28 | -1.390782353 | 4.206079513 |
| MRPS23 | -1.389478008 | 4.206079513 |

| | | |
|---|---|---|
| ZBTB24 | -1.386602244 | 4.206079513 |
| TPMT | -1.38659071 | 4.206079513 |
| ETF1 | -1.386317141 | 4.206079513 |
| TMEM147 | -1.384812194 | 4.206079513 |
| ZBTB11 | -1.384440771 | 4.206079513 |
| QSOX2 | -1.384317625 | 4.206079513 |

**Supplementary Table 1c. Genes upregulated in prednisolone-treated SUP-B15 cells.**

| Symbol | Fold Change | q-value(%) |
|---|---|---|
| OR7A5 | 22.5803248 | 0 |
| CALCRL | 13.76587745 | 0 |
| PLEKHG1 | 6.627158082 | 0 |
| SPRY1 | 5.646814869 | 0 |
| LILRA2 | 5.451680446 | 0 |
| DKK1 | 5.276774505 | 0 |
| OR7C1 | 5.22681948 | 0 |
| TMEM217 | 5.190412983 | 0 |
| DDIT4 | 5.109710896 | 0 |
| TP53INP1 | 4.993169909 | 0 |
| LDB3 | 4.91255423 | 0 |
| LONRF1 | 4.060003581 | 0 |
| P2RY14 | 4.013382518 | 0 |
| TMEM236 | 3.700222436 | 0 |
| SMIM3 | 3.63078793 | 0 |
| SYNE2 | 3.612863295 | 0 |
| MYO10 | 3.474943048 | 0 |
| EPAS1 | 3.467938546 | 0 |
| NFIL3 | 3.410880001 | 0 |
| NFKBIZ | 3.343396213 | 0 |
| YBX3 | 3.293492656 | 0 |
| DENND3 | 3.247079408 | 0 |
| EPHA4 | 3.242578872 | 0 |
| DPEP1 | 3.217954662 | 0 |
| NGFR | 3.161808519 | 0 |
| GP9 | 3.150634356 | 0 |
| RCAN1 | 3.106540078 | 0 |
| CHRNA1 | 3.096284417 | 0 |
| FAM43A | 3.063581716 | 0 |
| CTHRC1 | 2.824396564 | 0 |
| STAG3 | 2.768467655 | 0 |
| ELL2 | 2.708685072 | 0 |
| TMEM204 | 2.696857576 | 0 |
| BTG2 | 2.683188976 | 0 |
| TNFSF4 | 2.634472889 | 0 |
| FBXW7 | 2.624017485 | 0 |
| VPS37B | 2.551603802 | 0 |
| LDLRAD4 | 2.54799009 | 0 |
| SMAP2 | 2.530082333 | 0 |
| RASD1 | 2.43394123 | 0 |

| | | |
|---|---|---|
| RASAL2 | 2.361196868 | 0 |
| TXNIP | 2.355104376 | 0 |
| GADD45A | 2.342350547 | 0 |
| SLC44A1 | 2.341760174 | 0 |
| CYTH3 | 2.316462509 | 0 |
| TSC22D3 | 2.272022833 | 0 |
| KLF7 | 2.266720747 | 0 |
| SPTLC3 | 2.261312953 | 0 |
| KLF2 | 2.247357541 | 0 |
| DUSP1 | 2.235184622 | 0 |
| SAP30L | 2.227724252 | 0 |
| FKBP5 | 2.226219223 | 0 |
| ZC3H12D | 2.222539922 | 0 |
| PON2 | 2.200006318 | 0 |
| ITGA6 | 2.198150755 | 0 |
| RAPGEF5 | 2.094878153 | 0 |
| SMAD3 | 1.922719535 | 0 |
| KLF9 | 2.475494367 | 0.48155165 |
| SLC22A23 | 2.412055885 | 0.48155165 |
| BAALC | 2.3347256 | 0.48155165 |
| TGFB1I1 | 2.264186818 | 0.48155165 |
| BMP3 | 2.226475907 | 0.48155165 |
| EPC1 | 2.209137678 | 0.48155165 |
| GLIS3 | 2.142074019 | 0.48155165 |
| BEST3 | 2.138798149 | 0.48155165 |
| TLR5 | 2.135099543 | 0.48155165 |
| CPM | 2.104515303 | 0.48155165 |
| ATMIN | 2.089601735 | 0.48155165 |
| DDR1 | 2.075127527 | 0.48155165 |
| LMO2 | 2.059820089 | 0.48155165 |
| TLE1 | 2.041551083 | 0.48155165 |
| TRAK2 | 1.982043098 | 0.48155165 |
| UBASH3B | 1.972073059 | 0.48155165 |
| PAQR4 | 1.956125139 | 0.48155165 |
| MRC1 | 1.954960331 | 0.48155165 |
| BTBD3 | 1.931624499 | 0.48155165 |
| VAV3 | 1.92411763 | 0.48155165 |
| CD200 | 1.923457119 | 0.48155165 |
| RECK | 1.90799801 | 0.48155165 |
| F2RL3 | 1.855602035 | 0.48155165 |
| FAM168A | 1.839470662 | 0.48155165 |
| OLAH | 2.348253783 | 0.902909344 |
| STYK1 | 2.216796493 | 0.902909344 |

| | | |
|---|---|---|
| ISG20 | 2.192207965 | 0.902909344 |
| PIK3IP1 | 2.040740866 | 0.902909344 |
| RNASET2 | 2.024086109 | 0.902909344 |
| PLCH1 | 2.014436972 | 0.902909344 |
| DUSP6 | 1.969876704 | 0.902909344 |
| RGL1 | 1.959296857 | 0.902909344 |
| NFKBIA | 1.949268284 | 0.902909344 |
| MUC4 | 1.938451985 | 0.902909344 |
| GSN | 1.930569288 | 0.902909344 |
| SLC25A14 | 1.924918904 | 0.902909344 |
| TMSB4X | 1.91810939 | 0.902909344 |
| ANKRD33B | 1.91302934 | 0.902909344 |
| HS3ST3B1 | 1.902854168 | 0.902909344 |
| CD96 | 1.898833402 | 0.902909344 |
| INPP1 | 1.898479824 | 0.902909344 |
| TSC22D4 | 1.879265192 | 0.902909344 |
| CD53 | 1.877318369 | 0.902909344 |
| TNFRSF21 | 1.860865779 | 0.902909344 |
| CMTM2 | 1.851192597 | 0.902909344 |
| CYGB | 1.800269216 | 0.902909344 |
| IRF8 | 1.795604977 | 0.902909344 |
| TAP1 | 1.777300137 | 0.902909344 |
| MED13L | 1.763907613 | 0.902909344 |
| ABCC4 | 1.745681464 | 0.902909344 |
| BMP2 | 2.407575432 | 1.207124083 |
| EDNRB | 2.202968195 | 1.207124083 |
| NFATC1 | 1.985487576 | 1.207124083 |
| SHISA2 | 1.982076987 | 1.207124083 |
| FGD2 | 1.951442562 | 1.207124083 |
| PTHLH | 1.933280535 | 1.207124083 |
| CEACAM21 | 1.914534954 | 1.207124083 |
| IGFBP7 | 1.911559358 | 1.207124083 |
| SULF2 | 1.910407846 | 1.207124083 |
| IRAK3 | 1.909710564 | 1.207124083 |
| ASB13 | 1.90015881 | 1.207124083 |
| CD69 | 1.887556287 | 1.207124083 |
| ATP4B | 1.877593822 | 1.207124083 |
| METTL7A | 1.869252764 | 1.207124083 |
| ZIC2 | 1.856280418 | 1.207124083 |
| HECW1 | 1.844009387 | 1.207124083 |
| GCSAM | 1.830916926 | 1.207124083 |
| SLC9A9 | 1.824767091 | 1.207124083 |
| INSR | 1.77689652 | 1.207124083 |

| | | |
|---|---|---|
| FNBP1L | 1.769430791 | 1.207124083 |
| AHDC1 | 1.768981549 | 1.207124083 |
| WDR91 | 1.761442849 | 1.207124083 |
| AGPS | 1.760160109 | 1.207124083 |
| NR3C1 | 1.754498442 | 1.207124083 |
| SYNE3 | 1.736964578 | 1.207124083 |
| ARHGAP42 | 1.734698176 | 1.207124083 |
| TAPT1 | 1.732031298 | 1.207124083 |
| UBALD2 | 1.726698267 | 1.207124083 |
| VWA2 | 1.982194685 | 1.471407821 |
| MPV17L | 1.944892389 | 1.471407821 |
| OPN3 | 1.924405285 | 1.471407821 |
| BIK | 1.919445149 | 1.471407821 |
| KCNA5 | 1.903185256 | 1.471407821 |
| GRAMD1C | 1.876341812 | 1.471407821 |
| SOCS1 | 1.870002674 | 1.471407821 |
| RANBP3L | 1.806464621 | 1.471407821 |
| PREX1 | 1.792429447 | 1.471407821 |
| MYLK | 1.787651691 | 1.471407821 |
| ARHGEF28 | 1.784881547 | 1.471407821 |
| SLC16A2 | 1.760245515 | 1.471407821 |
| RASA3 | 1.745181397 | 1.471407821 |
| CAPN3 | 1.742508467 | 1.471407821 |
| F2R | 1.735582166 | 1.471407821 |
| TBC1D22B | 1.720784379 | 1.471407821 |
| LINGO3 | 1.704522921 | 1.471407821 |
| GTF2IRD2B | 1.697186765 | 1.471407821 |
| FZD6 | 1.687796539 | 1.471407821 |
| LIMD1 | 1.635342326 | 1.471407821 |
| KCNK3 | 1.917099655 | 1.818282724 |
| ITGA10 | 1.897051266 | 1.818282724 |
| LGALSL | 1.797612008 | 1.818282724 |
| SPRY4 | 1.793192454 | 1.818282724 |
| CLYBL | 1.762502536 | 1.818282724 |
| IFNLR1 | 1.729720177 | 1.818282724 |
| TTYH2 | 1.72759255 | 1.818282724 |
| MERTK | 1.722375059 | 1.818282724 |
| BCL2L11 | 1.710451662 | 1.818282724 |
| CCDC107 | 1.678288417 | 1.818282724 |
| PID1 | 1.662140495 | 1.818282724 |
| CHKA | 1.659228983 | 1.818282724 |
| PTPRE | 1.654776453 | 1.818282724 |
| FZD4 | 1.653325747 | 1.818282724 |

| | | |
|---|---|---|
| LY96 | 1.653000316 | 1.818282724 |
| TFPI | 1.640757848 | 1.818282724 |
| GLUL | 1.634060425 | 1.818282724 |
| LAIR1 | 1.63403966 | 1.818282724 |
| PRDM8 | 1.630256908 | 1.818282724 |
| QPCT | 1.615875449 | 1.818282724 |
| CDK19 | 1.612102341 | 1.818282724 |
| RAP1GAP2 | 1.608331726 | 1.818282724 |
| SMOX | 1.600438974 | 1.818282724 |
| PAG1 | 1.589056036 | 1.818282724 |
| SPTA1 | 1.839305341 | 2.70580083 |
| SH2D4B | 1.837090459 | 2.70580083 |
| STK32B | 1.78085987 | 2.70580083 |
| PIGV | 1.675611859 | 2.70580083 |
| SLC27A3 | 1.66935679 | 2.70580083 |
| WIPI1 | 1.650739344 | 2.70580083 |
| JUN | 1.647865698 | 2.70580083 |
| PRX | 1.636663047 | 2.70580083 |
| KLF6 | 1.620170151 | 2.70580083 |
| FUCA1 | 1.609858998 | 2.70580083 |
| KLHL24 | 1.608628293 | 2.70580083 |
| TCL1A | 1.605240729 | 2.70580083 |
| KLHL42 | 1.596888224 | 2.70580083 |
| SIK1 | 1.594626998 | 2.70580083 |
| PSTPIP2 | 1.584975273 | 2.70580083 |
| AAK1 | 1.573776132 | 2.70580083 |
| CDS2 | 1.564061238 | 2.70580083 |
| SERTAD1 | 1.563223795 | 2.70580083 |
| ST8SIA4 | 1.556896817 | 2.70580083 |
| TIPARP | 1.552473167 | 2.70580083 |
| TRPM8 | 1.540448374 | 2.70580083 |
| ADCY8 | 1.53822975 | 2.70580083 |
| NT5E | 1.536970704 | 2.70580083 |
| MLXIP | 1.533754596 | 2.70580083 |
| LRRFIP1 | 1.528429844 | 2.70580083 |
| SERINC5 | 1.527294208 | 2.70580083 |
| CCDC186 | 1.52704827 | 2.70580083 |
| SSH2 | 1.492768036 | 2.70580083 |
| PLA2G10 | 1.831451716 | 3.588485856 |
| NOS2 | 1.764962666 | 3.588485856 |
| LILRA1 | 1.74748249 | 3.588485856 |
| TUBB4A | 1.730968735 | 3.588485856 |
| NMRK1 | 1.705282786 | 3.588485856 |

| | | |
|---|---|---|
| DACT1 | 1.697824101 | 3.588485856 |
| GRB14 | 1.646615449 | 3.588485856 |
| PRKAG2 | 1.634031732 | 3.588485856 |
| ANKRD16 | 1.61051303 | 3.588485856 |
| PTP4A3 | 1.585902781 | 3.588485856 |
| SLC9C1 | 1.58187105 | 3.588485856 |
| TMEM140 | 1.576624028 | 3.588485856 |
| MDGA1 | 1.569898188 | 3.588485856 |
| CXCR4 | 1.569744764 | 3.588485856 |
| YPEL3 | 1.56762737 | 3.588485856 |
| FAM214B | 1.546182556 | 3.588485856 |
| WASF2 | 1.528577818 | 3.588485856 |
| DCTN4 | 1.525839272 | 3.588485856 |
| VPS26B | 1.510729117 | 3.588485856 |
| HMCES | 1.498272113 | 3.588485856 |
| CYFIP1 | 1.490458976 | 3.588485856 |
| AKNA | 1.486128982 | 3.588485856 |
| ARID5A | 1.480587411 | 3.588485856 |
| SLC9A7 | 1.465948072 | 3.588485856 |
| NRIP1 | 1.463290964 | 3.588485856 |
| DGKA | 1.457822427 | 3.588485856 |
| ZFP36L2 | 1.448320273 | 3.588485856 |

**Supplementary Table 1d. Genes downregulated in prednisolone-treated SUP-B15 cells.**

| Symbol | Fold Change | q-value(%) |
|---|---|---|
| GCSH | -5.312625436 | 0.66053545 |
| PUS7 | -2.600069764 | 0.66053545 |
| TLR10 | -2.598744861 | 0.66053545 |
| POLR3G | -2.430507636 | 0.66053545 |
| RGS13 | -2.28601415 | 0.66053545 |
| S1PR1 | -2.281929679 | 0.66053545 |
| NOP16 | -2.274210297 | 0.66053545 |
| PTGER4 | -2.193657055 | 0.66053545 |
| CHRNA5 | -2.148673586 | 0.66053545 |
| PLD6 | -2.133207048 | 0.66053545 |
| MYC | -2.090407202 | 0.66053545 |
| UGT3A2 | -2.089693952 | 0.66053545 |
| LYAR | -2.081314526 | 0.66053545 |
| WDR3 | -2.019523126 | 0.66053545 |
| PNO1 | -1.952533092 | 0.66053545 |
| CD55 | -1.949515106 | 0.66053545 |
| SFXN4 | -1.916491589 | 0.66053545 |
| SLC25A19 | -1.907125344 | 0.66053545 |
| SIX3 | -2.284846639 | 0.902909344 |
| RPL22L1 | -2.112512191 | 0.902909344 |
| MAP2K6 | -2.046432857 | 0.902909344 |
| TAF4B | -1.959096777 | 0.902909344 |
| PPAT | -1.870816425 | 0.902909344 |
| RPF2 | -1.857812623 | 0.902909344 |
| MRTO4 | -1.852947065 | 0.902909344 |
| RRP12 | -1.843225184 | 0.902909344 |
| GPATCH4 | -1.831279077 | 0.902909344 |
| ESF1 | -1.809410397 | 0.902909344 |
| IL7R | -1.771424528 | 0.902909344 |
| GBP6 | -2.111291332 | 1.207124083 |
| OSR2 | -1.996193166 | 1.207124083 |
| HSPA9 | -1.969074004 | 1.207124083 |
| METTL1 | -1.959769527 | 1.207124083 |
| GPR65 | -1.94894494 | 1.207124083 |
| RRS1 | -1.931080536 | 1.207124083 |
| CLUH | -1.860612985 | 1.207124083 |
| TFAP4 | -1.854245584 | 1.207124083 |
| HBEGF | -1.843743547 | 1.207124083 |
| RABEPK | -1.812556961 | 1.207124083 |
| FAM216A | -1.793100478 | 1.207124083 |

| | | |
|---|---|---|
| MYBBP1A | -1.7564019 | 1.207124083 |
| TAF1D | -1.753982881 | 1.207124083 |
| PAK1IP1 | -1.749410268 | 1.207124083 |
| NOL6 | -1.727434091 | 1.207124083 |
| DPH2 | -1.725492656 | 1.207124083 |
| WDR12 | -1.725031851 | 1.207124083 |
| DHX33 | -1.72110526 | 1.207124083 |
| DCTPP1 | -1.71880435 | 1.207124083 |
| CTSC | -1.714515959 | 1.207124083 |
| NLN | -1.705833298 | 1.207124083 |
| DDX21 | -1.698416156 | 1.207124083 |
| NAF1 | -1.669249568 | 1.207124083 |
| SLC8A1 | -2.240721997 | 1.471407821 |
| SAMHD1 | -1.945245173 | 1.471407821 |
| PCDH18 | -1.924849968 | 1.471407821 |
| RPL12 | -1.911206502 | 1.471407821 |
| MEX3A | -1.865068562 | 1.471407821 |
| IFIT1 | -1.83369327 | 1.471407821 |
| PPARGC1B | -1.805637976 | 1.471407821 |
| HK2 | -1.794502169 | 1.471407821 |
| MARS2 | -1.785794411 | 1.471407821 |
| CD244 | -1.78113722 | 1.471407821 |
| TLR6 | -1.778553863 | 1.471407821 |
| PDGFRB | -1.752628632 | 1.471407821 |
| SLC38A5 | -1.746388256 | 1.471407821 |
| RABGGTB | -1.729695399 | 1.471407821 |
| KLHL14 | -1.728550796 | 1.471407821 |
| FRG2 | -1.725347545 | 1.471407821 |
| RRP15 | -1.712804313 | 1.471407821 |
| MLKL | -1.712515445 | 1.471407821 |
| POLR1B | -1.7111944 | 1.471407821 |
| FKBP7 | -1.708601949 | 1.471407821 |
| RCL1 | -1.702347621 | 1.471407821 |
| NOC3L | -1.701393287 | 1.471407821 |
| PIGW | -1.700743608 | 1.471407821 |
| GNPNAT1 | -1.699939419 | 1.471407821 |
| RPP40 | -1.699076723 | 1.471407821 |
| PPRC1 | -1.694494945 | 1.471407821 |
| WDR43 | -1.684558659 | 1.471407821 |
| SLC9B2 | -1.676769833 | 1.471407821 |
| OAS2 | -1.663513222 | 1.471407821 |
| NOLC1 | -1.662982131 | 1.471407821 |
| PNPT1 | -1.657286469 | 1.471407821 |

| | | |
|---|---|---|
| DKC1 | -1.655267061 | 1.471407821 |
| NR1D1 | -1.653501094 | 1.471407821 |
| DIMT1 | -1.652218702 | 1.471407821 |
| SLC16A1 | -1.650301935 | 1.471407821 |
| MAK16 | -1.646192444 | 1.471407821 |
| UTP20 | -1.642388768 | 1.471407821 |
| TIMM8A | -1.642280243 | 1.471407821 |
| C3orf33 | -1.6387972 | 1.471407821 |
| CBX6 | -1.637335535 | 1.471407821 |
| NIFK | -1.630238828 | 1.471407821 |
| CCNJ | -1.62979593 | 1.471407821 |
| NUFIP1 | -1.624944 | 1.471407821 |
| TSR1 | -1.619939949 | 1.471407821 |
| NIP7 | -1.619029568 | 1.471407821 |
| XPO5 | -1.615791821 | 1.471407821 |
| KLF3 | -1.615576053 | 1.471407821 |
| POLR1C | -1.615410699 | 1.471407821 |
| TTLL12 | -1.612379859 | 1.471407821 |
| PDSS1 | -1.610087024 | 1.471407821 |
| ROBO1 | -1.603254363 | 1.471407821 |
| ODC1 | -1.599565422 | 1.471407821 |
| ATP2B1 | -1.598373977 | 1.471407821 |
| FJX1 | -1.595246092 | 1.471407821 |
| RIOK1 | -1.592077611 | 1.471407821 |
| GCH1 | -1.58830319 | 1.471407821 |
| SLC27A2 | -1.585866139 | 1.471407821 |
| NAA25 | -1.583995606 | 1.471407821 |
| GNL2 | -1.582289226 | 1.471407821 |
| CYSLTR1 | -1.909463929 | 1.818282724 |
| PTPN22 | -1.845296954 | 1.818282724 |
| REP15 | -1.801848857 | 1.818282724 |
| IL1B | -1.775948812 | 1.818282724 |
| ABCA9 | -1.774135664 | 1.818282724 |
| SLC10A5 | -1.740517516 | 1.818282724 |
| PODXL | -1.738889993 | 1.818282724 |
| GBP2 | -1.732744573 | 1.818282724 |
| SLFN12L | -1.732368686 | 1.818282724 |
| NOG | -1.720749392 | 1.818282724 |
| FDXACB1 | -1.705553488 | 1.818282724 |
| TBC1D30 | -1.698799591 | 1.818282724 |
| LGI2 | -1.693847118 | 1.818282724 |
| TRAF3 | -1.685104818 | 1.818282724 |
| CCDC86 | -1.682846592 | 1.818282724 |

| | | |
|---|---|---|
| SLC29A1 | -1.669924256 | 1.818282724 |
| KCNJ2 | -1.653959987 | 1.818282724 |
| ZNF57 | -1.650497553 | 1.818282724 |
| AP1AR | -1.637788807 | 1.818282724 |
| C12orf45 | -1.637031028 | 1.818282724 |
| LYSMD2 | -1.634463696 | 1.818282724 |
| AMPD2 | -1.632544895 | 1.818282724 |
| YME1L1 | -1.629366705 | 1.818282724 |
| CAMKK2 | -1.628296394 | 1.818282724 |
| FKBP14 | -1.628058643 | 1.818282724 |
| DDX31 | -1.626121437 | 1.818282724 |
| SLC25A33 | -1.623100501 | 1.818282724 |
| PWP2 | -1.616175274 | 1.818282724 |
| TMEM192 | -1.605220701 | 1.818282724 |
| KCNMB4 | -1.604621463 | 1.818282724 |
| CCDC58 | -1.598671663 | 1.818282724 |
| HTRA3 | -1.596509348 | 1.818282724 |
| PIK3C2B | -1.594591628 | 1.818282724 |
| RPS2 | -1.591215977 | 1.818282724 |
| PM20D2 | -1.590212979 | 1.818282724 |
| RPS27A | -1.584705768 | 1.818282724 |
| NLE1 | -1.581340813 | 1.818282724 |
| SLIRP | -1.574810969 | 1.818282724 |
| BYSL | -1.571848312 | 1.818282724 |
| ACACA | -1.570979477 | 1.818282724 |
| TOMM40 | -1.568197574 | 1.818282724 |
| NOP2 | -1.567608535 | 1.818282724 |
| TTC27 | -1.566868385 | 1.818282724 |
| LTV1 | -1.565598921 | 1.818282724 |
| SLC18B1 | -1.560664395 | 1.818282724 |
| DCUN1D5 | -1.556734952 | 1.818282724 |
| GTPBP4 | -1.552724276 | 1.818282724 |
| EXOSC5 | -1.552022709 | 1.818282724 |
| ABCE1 | -1.550832993 | 1.818282724 |
| POLR1A | -1.550275192 | 1.818282724 |
| SLC7A6 | -1.549972909 | 1.818282724 |
| UTP15 | -1.548517478 | 1.818282724 |
| TOMM5 | -1.547372636 | 1.818282724 |
| TRIAP1 | -1.545825353 | 1.818282724 |
| SCARB1 | -1.545480731 | 1.818282724 |
| AIMP2 | -1.544918072 | 1.818282724 |
| GNL3 | -1.541201327 | 1.818282724 |
| COA7 | -1.539433271 | 1.818282724 |

| | | |
|---|---|---|
| PDCD11 | -1.538851126 | 1.818282724 |
| GEMIN5 | -1.533974677 | 1.818282724 |
| TSEN2 | -1.53358805 | 1.818282724 |
| NDUFAF2 | -1.530402356 | 1.818282724 |
| PFDN2 | -1.529518616 | 1.818282724 |
| CCDC138 | -1.526252509 | 1.818282724 |
| FAM117B | -1.52440932 | 1.818282724 |
| WDR36 | -1.522905758 | 1.818282724 |
| FXN | -1.522409708 | 1.818282724 |
| YRDC | -1.51779225 | 1.818282724 |
| SRM | -1.506144302 | 1.818282724 |
| POLRMT | -1.504897959 | 1.818282724 |
| HOMER1 | -1.499777342 | 1.818282724 |
| KLRK1 | -1.840426753 | 2.70580083 |
| ANXA1 | -1.824766248 | 2.70580083 |
| GPR174 | -1.727649231 | 2.70580083 |
| MMP14 | -1.715991016 | 2.70580083 |
| TLR1 | -1.670947802 | 2.70580083 |
| ITGB7 | -1.655033019 | 2.70580083 |
| FZD3 | -1.648291418 | 2.70580083 |
| ALDH1B1 | -1.620794294 | 2.70580083 |
| RPS26 | -1.618276355 | 2.70580083 |
| PACSIN3 | -1.605957447 | 2.70580083 |
| SLC4A4 | -1.60529896 | 2.70580083 |
| SLC39A14 | -1.603796024 | 2.70580083 |
| PES1 | -1.593715009 | 2.70580083 |
| GABBR2 | -1.592556621 | 2.70580083 |
| IL21R | -1.591106421 | 2.70580083 |
| LOC102723899 | -1.583909603 | 2.70580083 |
| WDR89 | -1.576397099 | 2.70580083 |
| TRIB2 | -1.568283087 | 2.70580083 |
| NDUFAF4 | -1.567450627 | 2.70580083 |
| MIEF1 | -1.561558553 | 2.70580083 |
| NT5DC3 | -1.559310246 | 2.70580083 |
| SCFD2 | -1.559269174 | 2.70580083 |
| TXLNG | -1.552858097 | 2.70580083 |
| SLC25A37 | -1.548874587 | 2.70580083 |
| TAF13 | -1.548380453 | 2.70580083 |
| MAST3 | -1.547886476 | 2.70580083 |
| UBE3D | -1.543971726 | 2.70580083 |
| ELOVL6 | -1.543155736 | 2.70580083 |
| ARMC10 | -1.542591073 | 2.70580083 |
| ZNF749 | -1.54127326 | 2.70580083 |

| | | |
|---|---|---|
| ZNRF3 | -1.540993027 | 2.70580083 |
| MYOCD | -1.540815726 | 2.70580083 |
| MRM1 | -1.540801486 | 2.70580083 |
| HMGA2 | -1.540004607 | 2.70580083 |
| AMER1 | -1.538950683 | 2.70580083 |
| RPL21 | -1.538388269 | 2.70580083 |
| FASTKD3 | -1.536675277 | 2.70580083 |
| IFIT5 | -1.536513739 | 2.70580083 |
| CALML4 | -1.531637265 | 2.70580083 |
| ALG1 | -1.530681371 | 2.70580083 |
| SRFBP1 | -1.52836628 | 2.70580083 |
| IPO4 | -1.526090656 | 2.70580083 |
| BRIX1 | -1.52213607 | 2.70580083 |
| PDE3B | -1.520115909 | 2.70580083 |
| GRPEL1 | -1.519346578 | 2.70580083 |
| GAR1 | -1.518321876 | 2.70580083 |
| PFKM | -1.517414259 | 2.70580083 |
| FAM162A | -1.516591631 | 2.70580083 |
| NAMPT | -1.514663516 | 2.70580083 |
| BZW2 | -1.512069725 | 2.70580083 |
| THUMPD2 | -1.507968194 | 2.70580083 |
| LIMA1 | -1.507742439 | 2.70580083 |
| PDCD2L | -1.50714024 | 2.70580083 |
| SRPRB | -1.506709201 | 2.70580083 |
| ADAT2 | -1.50432644 | 2.70580083 |
| RPL32 | -1.498329925 | 2.70580083 |
| URB2 | -1.497143318 | 2.70580083 |
| RRP1B | -1.497112877 | 2.70580083 |
| PUS7L | -1.497059955 | 2.70580083 |
| PINX1 | -1.496982476 | 2.70580083 |
| SACS | -1.495902348 | 2.70580083 |
| SLC35F2 | -1.49285047 | 2.70580083 |
| MAMLD1 | -1.492113899 | 2.70580083 |
| UTP3 | -1.489771085 | 2.70580083 |
| EIF1AX | -1.489245225 | 2.70580083 |
| EIF3J | -1.485163056 | 2.70580083 |
| TMEM38B | -1.483707803 | 2.70580083 |
| UTP18 | -1.481180369 | 2.70580083 |
| PDP2 | -1.48025015 | 2.70580083 |
| HEATR3 | -1.480194061 | 2.70580083 |
| PMM2 | -1.479394687 | 2.70580083 |
| ISOC1 | -1.478628883 | 2.70580083 |
| CD22 | -1.477942355 | 2.70580083 |

| | | |
|---|---|---|
| ZNHIT6 | -1.477364688 | 2.70580083 |
| IDH3A | -1.475779997 | 2.70580083 |
| PTRH2 | -1.474802057 | 2.70580083 |
| NARS2 | -1.474355058 | 2.70580083 |
| RPL37 | -1.471647689 | 2.70580083 |
| NKRF | -1.471261814 | 2.70580083 |
| PTPN2 | -1.470949788 | 2.70580083 |
| MDN1 | -1.470154387 | 2.70580083 |
| L3MBTL3 | -1.47004977 | 2.70580083 |
| DNAJC2 | -1.470020221 | 2.70580083 |
| PPIF | -1.466115402 | 2.70580083 |
| RPS3A | -1.465306365 | 2.70580083 |
| ALDH18A1 | -1.463907774 | 2.70580083 |
| GDF11 | -1.463896274 | 2.70580083 |
| GPN3 | -1.462351708 | 2.70580083 |
| LGR5 | -1.460281663 | 2.70580083 |
| CAD | -1.457231413 | 2.70580083 |
| PPIL1 | -1.45480891 | 2.70580083 |
| SNAPC1 | -1.454361251 | 2.70580083 |
| SLC17A9 | -1.452345468 | 2.70580083 |
| WDR75 | -1.451082958 | 2.70580083 |
| SCD | -1.446303172 | 2.70580083 |
| ZC3H8 | -1.445584224 | 2.70580083 |
| FGD6 | -1.442597005 | 2.70580083 |
| FTSJ1 | -1.442389367 | 2.70580083 |
| EEF2K | -1.44041181 | 2.70580083 |
| CLEC2B | -1.43928537 | 2.70580083 |
| LDHA | -1.438686913 | 2.70580083 |
| RPS25 | -1.438341251 | 2.70580083 |
| TEX2 | -1.433887993 | 2.70580083 |
| SEH1L | -1.428617798 | 2.70580083 |
| HEATR1 | -1.421764647 | 2.70580083 |
| AIF1 | -2.146795348 | 3.588485856 |
| ZDHHC11 | -1.796080899 | 3.588485856 |
| RPS18 | -1.753328919 | 3.588485856 |
| PDE3A | -1.625967777 | 3.588485856 |
| TRIB1 | -1.60952576 | 3.588485856 |
| PLXDC2 | -1.596085201 | 3.588485856 |
| EVI5 | -1.587003526 | 3.588485856 |
| NKIRAS1 | -1.572633327 | 3.588485856 |
| TMEM117 | -1.565564557 | 3.588485856 |
| PLCG2 | -1.555572533 | 3.588485856 |
| DHODH | -1.544203263 | 3.588485856 |

| | | |
|---|---|---|
| CLEC2D | -1.537991646 | 3.588485856 |
| SLC25A15 | -1.529545828 | 3.588485856 |
| ASXL2 | -1.526852819 | 3.588485856 |
| POLR3K | -1.518420105 | 3.588485856 |
| NR1D2 | -1.517606749 | 3.588485856 |
| SLC19A2 | -1.517604645 | 3.588485856 |
| GPR155 | -1.504793303 | 3.588485856 |
| LRRC69 | -1.501233095 | 3.588485856 |
| PRICKLE2 | -1.49739032 | 3.588485856 |
| IFIT2 | -1.495464503 | 3.588485856 |
| PDPN | -1.493681963 | 3.588485856 |
| E2F5 | -1.492198366 | 3.588485856 |
| RPS28 | -1.492138032 | 3.588485856 |
| EXOSC7 | -1.487873287 | 3.588485856 |
| RFTN1 | -1.482634513 | 3.588485856 |
| C15orf61 | -1.482534146 | 3.588485856 |
| SNTB1 | -1.480208083 | 3.588485856 |
| MYLIP | -1.479255918 | 3.588485856 |
| ITPRIPL1 | -1.478970217 | 3.588485856 |
| COQ3 | -1.472298637 | 3.588485856 |
| TMEM177 | -1.472172098 | 3.588485856 |
| UBQLN4 | -1.46939099 | 3.588485856 |
| UBE2G2 | -1.467787721 | 3.588485856 |
| DDX10 | -1.466547365 | 3.588485856 |
| MCTP1 | -1.466031735 | 3.588485856 |
| FCHO1 | -1.465916911 | 3.588485856 |
| POLR3D | -1.462797771 | 3.588485856 |
| NEXN | -1.462760594 | 3.588485856 |
| EARS2 | -1.461790943 | 3.588485856 |
| PFAS | -1.461663618 | 3.588485856 |
| POLR3B | -1.457400779 | 3.588485856 |
| SELL | -1.455820011 | 3.588485856 |
| SLC16A7 | -1.454434507 | 3.588485856 |
| PRMT5 | -1.453053007 | 3.588485856 |
| UTP14A | -1.451169125 | 3.588485856 |
| FAIM | -1.450436363 | 3.588485856 |
| HSPH1 | -1.447844503 | 3.588485856 |
| CMSS1 | -1.447507008 | 3.588485856 |
| MTHFD1L | -1.444386993 | 3.588485856 |
| NADK2 | -1.443621298 | 3.588485856 |
| ARMC6 | -1.443231434 | 3.588485856 |
| GEMIN2 | -1.442780337 | 3.588485856 |
| RRP1 | -1.442421361 | 3.588485856 |

| | | |
|---|---|---|
| IKBIP | -1.441510821 | 3.588485856 |
| DTD2 | -1.440815227 | 3.588485856 |
| PRDX4 | -1.439991538 | 3.588485856 |
| NOP56 | -1.438869083 | 3.588485856 |
| GPR135 | -1.438639047 | 3.588485856 |
| CD48 | -1.438330616 | 3.588485856 |
| ZNF593 | -1.436650698 | 3.588485856 |
| STAMBPL1 | -1.436647379 | 3.588485856 |
| DHX37 | -1.436485403 | 3.588485856 |
| UCHL3 | -1.436159184 | 3.588485856 |
| FKBP11 | -1.435515591 | 3.588485856 |
| EIF2B3 | -1.433849894 | 3.588485856 |
| FASTKD2 | -1.433465318 | 3.588485856 |
| ZNF485 | -1.430642957 | 3.588485856 |
| TIMM9 | -1.429141072 | 3.588485856 |
| FAM98A | -1.427461333 | 3.588485856 |
| GLDC | -1.427260161 | 3.588485856 |
| CYTIP | -1.42518842 | 3.588485856 |
| SNRPD1 | -1.424975057 | 3.588485856 |
| REXO2 | -1.424240056 | 3.588485856 |
| POLR3H | -1.424004462 | 3.588485856 |
| PRMT3 | -1.423697195 | 3.588485856 |
| OGFOD1 | -1.421995599 | 3.588485856 |
| FARSA | -1.421810309 | 3.588485856 |
| TRIM25 | -1.42002402 | 3.588485856 |
| TAF1A | -1.418487398 | 3.588485856 |
| METTL16 | -1.417345355 | 3.588485856 |
| DNAJA1 | -1.416211431 | 3.588485856 |
| BRMS1L | -1.415525756 | 3.588485856 |
| ERCC8 | -1.414932928 | 3.588485856 |
| LIAS | -1.413919516 | 3.588485856 |
| ARL14EP | -1.412261909 | 3.588485856 |
| AMMECR1 | -1.41159902 | 3.588485856 |
| TFRC | -1.410363783 | 3.588485856 |
| PSAT1 | -1.409561736 | 3.588485856 |
| MRPL3 | -1.40928103 | 3.588485856 |
| HSPE1-MOB4 | -1.407247742 | 3.588485856 |
| IMPDH1 | -1.407136873 | 3.588485856 |
| AGPAT5 | -1.406987326 | 3.588485856 |
| WDR74 | -1.406303843 | 3.588485856 |
| PTER | -1.404440333 | 3.588485856 |
| CHCHD4 | -1.404415996 | 3.588485856 |
| TRUB1 | -1.403947512 | 3.588485856 |

| | | |
|---|---|---|
| POP1 | -1.40362479 | 3.588485856 |
| CBWD1 | -1.40355993 | 3.588485856 |
| OTUD6B | -1.403137767 | 3.588485856 |
| EBPL | -1.402851857 | 3.588485856 |
| ISOC2 | -1.402166171 | 3.588485856 |
| DDX55 | -1.401944917 | 3.588485856 |
| DLAT | -1.400746933 | 3.588485856 |
| FARSB | -1.399828416 | 3.588485856 |
| TNPO2 | -1.397993836 | 3.588485856 |
| SPOPL | -1.397512964 | 3.588485856 |
| NME1 | -1.39733377 | 3.588485856 |
| NOL10 | -1.396792451 | 3.588485856 |
| ENOPH1 | -1.395860724 | 3.588485856 |
| TRMT6 | -1.395805253 | 3.588485856 |
| SUPV3L1 | -1.395416373 | 3.588485856 |
| QSOX2 | -1.392025848 | 3.588485856 |
| TRIT1 | -1.391944479 | 3.588485856 |
| ZBTB24 | -1.390405474 | 3.588485856 |
| GART | -1.390255457 | 3.588485856 |
| RPL37A | -1.389858168 | 3.588485856 |
| NOP14 | -1.389678028 | 3.588485856 |
| RSC1A1 | -1.389637572 | 3.588485856 |
| COA4 | -1.388887103 | 3.588485856 |
| MRPL4 | -1.388328206 | 3.588485856 |
| AHSA1 | -1.386105754 | 3.588485856 |
| RNMT | -1.382642011 | 3.588485856 |
| ZNF330 | -1.380273343 | 3.588485856 |
| TFB2M | -1.380160134 | 3.588485856 |
| STIP1 | -1.379417333 | 3.588485856 |
| AMD1 | -1.379096426 | 3.588485856 |
| NOL11 | -1.378726855 | 3.588485856 |
| TNFAIP8 | -1.378656775 | 3.588485856 |
| NOP58 | -1.372471952 | 3.588485856 |
| NSUN2 | -1.371393573 | 3.588485856 |
| ETF1 | -1.370610204 | 3.588485856 |
| NCLN | -1.369389004 | 3.588485856 |

**Supplementary Table 2**

**Supplementary Table 2a. Upregulated genes in predicted dexamethasone-resistant ALL patient samples.**

| Gene ID | Fold Change | q-value(%) |
|---|---|---|
| CDC5L | 1.908343434 | 0 |
| ZNF595 | 1.886096977 | 0 |
| TCTN2 | 1.848335137 | 0 |
| LOC105373316 | 1.815109422 | 0 |
| SH3GL3 | 1.759268232 | 0 |
| FRG1BP | 1.716146765 | 0 |
| TRIM4 | 1.707238752 | 0 |
| LOC101928669 | 1.706305019 | 0 |
| PNO1 | 1.698017594 | 0 |
| OPHN1 | 1.694889268 | 0 |
| LOC441666 | 1.688837148 | 0 |
| EPOR | 1.688388634 | 0 |
| MEFV | 1.675073925 | 0 |
| LOC100505609 | 1.666746873 | 0 |
| ANKRD20A5P | 1.663465274 | 0 |
| CASP2 | 1.654110728 | 0 |
| SCD5 | 1.652008868 | 0 |
| RASEF | 1.649228156 | 0 |
| FKSG52 | 1.642902835 | 0 |
| SLC25A16 | 1.630771403 | 0 |
| ANKRD20A1 | 1.629848837 | 0 |
| SLC4A4 | 1.624951056 | 0 |
| COLCA1 | 1.621579714 | 0 |
| DNAH3 | 1.620091278 | 0 |
| LINC00408 | 1.618077557 | 0 |
| TMEM241 | 1.600932391 | 0 |
| TMEFF2 | 1.597763334 | 0 |
| MCM3AP | 1.594145108 | 0 |
| ZNF528 | 1.575978890 | 0 |
| ZNF445 | 1.559334495 | 0 |
| RECK | 1.552273425 | 0 |
| LOC101928288 | 1.523762697 | 0 |
| ZNF747 | 1.522292994 | 0 |
| DDX58 | 1.519879834 | 0 |
| DDX60L | 1.506936514 | 0 |
| LOC105371220 | 1.511930869 | 0.022862369 |
| PBX1 | 1.821879410 | 0.042478845 |
| ARL4C | 1.595735274 | 0.042478845 |
| HIP1R | 1.523309341 | 0.042478845 |
| CMTM7 | 1.514422261 | 0.042478845 |

| | | |
|---|---|---|
| MARCKS | 1.625460794 | 0.173850264 |
| XIST | 2.640412771 | 0.244260609 |
| SEMA6A | 1.531761069 | 0.244260609 |
| JCHAIN | 1.882073199 | 0.303598806 |
| CMTM8 | 1.589000713 | 0.303598806 |
| ID3 | 1.588781672 | 0.303598806 |
| NID2 | 1.549380310 | 0.396061751 |
| ADARB1 | 1.503822423 | 0.396061751 |
| MTCL1 | 1.504084753 | 0.428147117 |

**Supplementary Table 2b. Downregulated genes in predicted dexamethasone-resistant ALL patient samples.**

| Gene ID | Fold Change | q-value(%) |
|---|---|---|
| NR3C1 | -1.997719279 | 0 |
| DUSP28 | -1.635969944 | 0 |
| MSH6 | -2.684594433 | 0 |
| IPO7 | -2.118141557 | 0 |
| WIPF1 | -2.267097449 | 0 |
| KPNB1 | -1.797594179 | 0 |
| ARL5A | -1.906965185 | 0 |
| PPIP5K2 | -1.852203159 | 0 |
| TMEM18 | -1.521248745 | 0 |
| BCL2 | -2.286265421 | 0 |
| EVI2A | -2.180478944 | 0 |
| CMC1 | -1.764996590 | 0 |
| FEZ2 | -1.972316324 | 0 |
| RPL37 | -1.615830927 | 0 |
| NCBP3 | -1.651488344 | 0 |
| SNAP23 | -1.928256001 | 0 |
| C11orf54 | -1.862120559 | 0 |
| AKAP11 | -1.737482260 | 0 |
| MKRN2 | -1.526548752 | 0 |
| PFAS | -1.830265473 | 0 |
| NFATC3 | -1.766044577 | 0 |
| MAP4K5 | -1.676022489 | 0 |
| HNRNPU | -1.657601283 | 0 |
| ITGA4 | -2.782119127 | 0 |
| TIMM9 | -1.539754159 | 0 |
| DDX10 | -1.593756426 | 0 |
| FOPNL | -1.763827053 | 0 |
| ATP8A1 | -1.977734526 | 0 |
| TMEM65 | -1.966683328 | 0 |
| TOP1MT | -1.848355574 | 0 |
| SORL1 | -2.724646415 | 0 |
| DFFA | -1.568384836 | 0 |
| URI1 | -1.650235959 | 0 |
| GMPR2 | -1.666075575 | 0 |
| CUL4B | -1.719986466 | 0 |
| AGTPBP1 | -1.711867054 | 0 |
| TMX1 | -1.990084626 | 0 |
| RASA4 | -1.744497489 | 0 |
| HTATIP2 | -2.031289104 | 0 |
| STARD3NL | -1.747599865 | 0 |

| | | |
|---|---|---|
| GALNT1 | -1.806148926 | 0 |
| LETMD1 | -1.543100887 | 0 |
| MED30 | -1.860415263 | 0 |
| PDCD2 | -1.600116985 | 0 |
| ABCE1 | -1.588301788 | 0 |
| KPNA4 | -1.758653794 | 0 |
| PCBP1 | -1.597808968 | 0 |
| XPOT | -1.672916814 | 0 |
| PA2G4 | -1.656985114 | 0 |
| MRPL33 | -1.715155053 | 0 |
| IKBKB | -1.720216615 | 0 |
| RALGAPA1 | -1.780449797 | 0 |
| FTSJ1 | -1.760113838 | 0 |
| PPP1R21 | -1.857075857 | 0 |
| MRPL45 | -1.514156292 | 0 |
| EMC4 | -1.733623729 | 0 |
| MRPS18B | -1.558482808 | 0 |
| SMAD2 | -1.923857709 | 0 |
| COMMD9 | -1.521114833 | 0 |
| NTPCR | -1.750871470 | 0 |
| DYM | -1.675077694 | 0 |
| BTF3L4 | -1.607394195 | 0 |
| DOCK8 | -2.066664879 | 0 |
| WDR45 | -1.677623986 | 0 |
| HNRNPUL2 | -1.685064032 | 0 |
| UBASH3B | -2.807525163 | 0 |
| RPL31 | -2.002432696 | 0 |
| TTC37 | -1.874687344 | 0 |
| MRPL3 | -1.998535899 | 0 |
| PRKDC | -1.953997304 | 0 |
| METTL7A | -2.516160495 | 0 |
| TMEM69 | -1.608954131 | 0 |
| INPP4A | -1.707830670 | 0 |
| MIS12 | -1.613336695 | 0 |
| STT3B | -1.975180868 | 0 |
| SUB1 | -1.887618555 | 0 |
| EBLN3 | -1.984264025 | 0 |
| UBA2 | -1.718406996 | 0 |
| NUDT5 | -1.787660715 | 0 |
| EXOSC8 | -1.761959665 | 0 |
| MTR | -1.665903673 | 0 |
| ABCB7 | -1.504155999 | 0 |
| GFM2 | -1.679253888 | 0 |
| INO80C | -1.639299338 | 0 |

| | | |
|---|---|---|
| EBAG9 | -1.767571235 | 0 |
| RPL7L1 | -1.593480753 | 0 |
| IKZF1 | -2.046521334 | 0 |
| SLC25A32 | -1.758857213 | 0 |
| CRBN | -1.854036911 | 0 |
| FLT3 | -3.209042243 | 0 |
| RNASEH2B | -1.628440839 | 0 |
| ZNF280D | -1.606967842 | 0 |
| SMIM20 | -1.693126467 | 0 |
| RNF5 | -1.526615377 | 0 |
| LINC00493 | -1.751203943 | 0 |
| BRE | -1.561152226 | 0 |
| UPF3A | -1.812677818 | 0 |
| CAPN3 | -2.191019542 | 0 |
| CRNDE | -2.272950541 | 0 |
| DHX33 | -1.613766881 | 0 |
| SERINC3 | -1.560432851 | 0 |
| SERBP1 | -1.879757352 | 0 |
| CEP63 | -1.589677987 | 0 |
| SATB1 | -1.698566567 | 0 |
| JAGN1 | -1.534317343 | 0 |
| GNAQ | -1.886000442 | 0 |
| FAM122B | -1.942590794 | 0 |
| MRPL57 | -1.841840127 | 0 |
| NADK2 | -1.576785005 | 0 |
| EHBP1 | -1.839973650 | 0 |
| MAVS | -1.536871744 | 0 |
| ICE2 | -1.667781195 | 0 |
| ANAPC16 | -2.220343933 | 0 |
| TMX2 | -1.638718731 | 0 |
| ADAT2 | -1.535893062 | 0 |
| OXR1 | -1.578075167 | 0 |
| SMC1A | -1.850331037 | 0 |
| SLK | -1.534511888 | 0 |
| FBXO38 | -1.561082759 | 0 |
| PGM2 | -1.742511947 | 0 |
| IFT80 | -1.879602618 | 0 |
| HERC2 | -1.634568815 | 0 |
| ZNF277 | -1.697220243 | 0 |
| MFSD1 | -1.849692106 | 0 |
| UHRF1 | -1.920147434 | 0 |
| TRMT1 | -1.617369200 | 0 |
| CASP1 | -2.179583295 | 0 |
| KIZ | -1.598753931 | 0 |

| | | |
|---|---|---|
| FUNDC2 | -1.650962550 | 0 |
| CLNS1A | -1.960512493 | 0 |
| TSPAN31 | -1.782571836 | 0 |
| OSTC | -1.641209122 | 0 |
| DNAJC10 | -1.839414818 | 0 |
| NFU1 | -1.583188562 | 0 |
| TMEM126B | -1.640741807 | 0 |
| TOR1B | -1.704501269 | 0 |
| MDN1 | -1.569559542 | 0 |
| ELAVL1 | -1.802767612 | 0 |
| UQCRC2 | -1.825052308 | 0 |
| CCT6A | -1.639520931 | 0 |
| EHD4 | -1.536542194 | 0 |
| DNAJB6 | -1.530297142 | 0 |
| TPST2 | -1.722718854 | 0 |
| TRAPPC2 | -1.820056867 | 0 |
| FBXW2 | -1.892021836 | 0 |
| ZMYM4 | -1.530276836 | 0 |
| PURA | -1.621007701 | 0 |
| ROCK2 | -1.630989261 | 0 |
| MBD4 | -1.724599877 | 0 |
| SEH1L | -1.583864326 | 0 |
| MED13L | -1.700379206 | 0 |
| LYRM7 | -1.789228662 | 0 |
| MCTS1 | -1.982529263 | 0 |
| UTP14A | -1.624316597 | 0 |
| CRACR2A | -1.733819404 | 0 |
| TCF12 | -1.933858923 | 0 |
| PRMT5 | -1.729174334 | 0 |
| UBE2G2 | -1.522891748 | 0 |
| PRELID3B | -1.546731193 | 0 |
| METTL23 | -1.867072427 | 0 |
| IPO5 | -1.821201266 | 0 |
| C14orf2 | -1.633173204 | 0 |
| MAML3 | -1.809247404 | 0 |
| SMIM10L1 | -1.632120420 | 0 |
| COPS5 | -1.736398389 | 0 |
| ALG13 | -1.688170535 | 0 |
| RTCA | -1.556389443 | 0 |
| EIF3J | -1.818599420 | 0 |
| PAN3 | -1.552792538 | 0 |
| ATL3 | -1.824741965 | 0 |
| CREB3L4 | -1.511170253 | 0 |
| C11orf58 | -1.826793568 | 0 |

| | | |
|---|---|---|
| DTX3L | -1.761699725 | 0 |
| RAB13 | -1.779997455 | 0 |
| CEP57 | -1.643966462 | 0 |
| VPS36 | -1.711071197 | 0 |
| MTIF2 | -1.665685529 | 0 |
| CHCHD3 | -1.562716435 | 0 |
| RABEP1 | -1.663424823 | 0 |
| KATNBL1 | -1.593287261 | 0 |
| KIAA0430 | -1.688659384 | 0 |
| AIMP1 | -1.602466787 | 0 |
| MRPS33 | -1.585436716 | 0 |
| CANX | -1.819536400 | 0 |
| SEC31A | -1.689864584 | 0 |
| IKBKAP | -1.699394725 | 0 |
| NDEL1 | -1.588479459 | 0 |
| ST13 | -1.791308898 | 0 |
| CCT4 | -1.546073628 | 0 |
| C1QBP | -1.901628686 | 0 |
| ZFAS1 | -1.660671574 | 0 |
| UBE2V1 | -1.548247254 | 0 |
| TMOD3 | -1.575207183 | 0 |
| HSD17B11 | -1.752549252 | 0 |
| SYNCRIP | -1.773879589 | 0 |
| PPP3CA | -1.989150397 | 0 |
| RPL35A | -1.517933869 | 0 |
| DERA | -1.830680181 | 0 |
| PPP4R3B | -1.641015883 | 0 |
| POLR1D | -1.674512180 | 0 |
| ESYT2 | -1.927153216 | 0 |
| FKBP5 | -2.384088542 | 0 |
| OGT | -1.736288617 | 0 |
| TATDN1 | -1.628283027 | 0 |
| ME2 | -1.873373112 | 0 |
| DENR | -1.665528494 | 0 |
| TRMT61B | -1.529508137 | 0 |
| PBDC1 | -1.745022718 | 0 |
| NCOA1 | -1.718855479 | 0 |
| EIF2AK4 | -1.746711416 | 0 |
| TMEM135 | -1.669386219 | 0 |
| RPL13 | -1.557362580 | 0 |
| WDSUB1 | -1.731303979 | 0 |
| FAM162A | -1.753957994 | 0 |
| LRPPRC | -1.673651099 | 0 |
| NARS2 | -1.530219875 | 0 |

| | | |
|---|---|---|
| DCP2 | -1.551164294 | 0 |
| COPZ1 | -1.585249440 | 0 |
| VPS13C | -1.607546639 | 0 |
| TMEM126A | -1.700994921 | 0 |
| MEF2A | -1.894349810 | 0 |
| HADH | -1.761381557 | 0 |
| ATF1 | -1.683775864 | 0 |
| SLC22A16 | -1.661909678 | 0 |
| CASP4 | -1.643695971 | 0 |
| CNDP2 | -1.724579474 | 0 |
| ZCCHC7 | -1.777556288 | 0 |
| MRPS27 | -1.600550906 | 0 |
| FAM129C | -1.760219402 | 0 |
| PM20D2 | -1.765743224 | 0 |
| SRP72 | -1.638318226 | 0 |
| TPGS2 | -1.567959299 | 0 |
| SERP1 | -1.635410209 | 0 |
| EPRS | -1.506991677 | 0 |
| AK2 | -1.877153306 | 0 |
| ECHDC1 | -1.933276493 | 0 |
| LY75 | -1.812516015 | 0 |
| SF3A1 | -1.595513719 | 0 |
| WDR61 | -1.874838459 | 0 |
| ELP3 | -1.530951802 | 0 |
| XAF1 | -2.137642188 | 0 |
| ATP5S | -1.553037440 | 0 |
| TRAF3IP2 | -1.672711343 | 0 |
| RHOT1 | -1.577810649 | 0 |
| PCMTD1 | -1.667794069 | 0 |
| PPP2R3C | -1.724589662 | 0 |
| BOLA2 | -1.567986608 | 0 |
| RFX5 | -1.544459291 | 0 |
| ILF3 | -1.689634278 | 0 |
| NIFK | -1.647097163 | 0 |
| TMEM131 | -1.776944841 | 0 |
| MRPL15 | -1.771596999 | 0 |
| MRPL1 | -1.594797611 | 0 |
| NFYA | -1.619084944 | 0 |
| IQGAP2 | -1.836381926 | 0 |
| ZC3H4 | -1.610244173 | 0 |
| ZDHHC6 | -1.507272869 | 0 |
| PPP1R11 | -1.767345146 | 0 |
| DDX18 | -1.523543146 | 0 |
| TAOK3 | -1.696229370 | 0 |

| | | |
|---|---|---|
| ZNF121 | -1.759773798 | 0 |
| RNF24 | -1.772223027 | 0 |
| AKAP1 | -1.615929889 | 0 |
| LOC101927027 | -1.665325347 | 0 |
| ZC3H14 | -1.551575793 | 0 |
| NDUFS4 | -1.598711973 | 0 |
| PAN3-AS1 | -1.711875892 | 0 |
| FAM134A | -1.595177594 | 0 |
| PSMG4 | -1.656107630 | 0 |
| IARS | -1.730364333 | 0 |
| ZAK | -2.096452080 | 0 |
| GPN3 | -1.502808650 | 0 |
| NCOA6 | -1.503055279 | 0 |
| FAM216A | -1.583752770 | 0 |
| NMRAL1 | -1.643090605 | 0 |
| CMSS1 | -1.656558298 | 0 |
| CBL | -1.574527561 | 0 |
| DDHD2 | -1.721193506 | 0 |
| TMED10 | -1.779932903 | 0 |
| HMG20B | -1.657533508 | 0 |
| INTS8 | -1.582938179 | 0 |
| PHKB | -1.739278015 | 0 |
| GYG1 | -2.086381375 | 0 |
| SF3A3 | -1.643025320 | 0 |
| POLR3GL | -1.586354289 | 0 |
| RPA2 | -1.526879668 | 0 |
| LAP3 | -1.949860642 | 0 |
| MBTPS1 | -1.559585231 | 0 |
| TPRKB | -1.765749236 | 0 |
| LONP2 | -1.655086756 | 0 |
| NAA20 | -1.724451005 | 0 |
| ATR | -1.662075222 | 0 |
| ZSWIM7 | -1.554436147 | 0 |
| SNHG16 | -1.669226161 | 0 |
| VDAC3 | -1.590872872 | 0 |
| AHCYL1 | -1.576276710 | 0 |
| TXNL4A | -1.515846690 | 0 |
| INTS3 | -1.600364467 | 0 |
| DHTKD1 | -1.747317772 | 0 |
| METTL5 | -1.906455666 | 0 |
| DPYD | -1.773320168 | 0 |
| ESYT1 | -1.582151045 | 0 |
| CHMP4A | -1.656165646 | 0 |
| DPH3 | -1.691052979 | 0 |

| | | |
|---|---|---|
| INSR | -1.522688118 | 0 |
| LARS | -1.846280541 | 0 |
| PAICS | -1.773634656 | 0 |
| SH3YL1 | -1.819866863 | 0 |
| ATP5L | -1.567454251 | 0 |
| TAX1BP3 | -1.924169451 | 0 |
| SIDT2 | -1.611232080 | 0 |
| GAS5 | -1.825025113 | 0 |
| TIA1 | -2.098237009 | 0 |
| LIMD1 | -1.616762584 | 0 |
| CTSA | -1.605626180 | 0 |
| RNF114 | -1.661498832 | 0 |
| NIPSNAP3A | -1.697324264 | 0 |
| RAB8A | -1.701051438 | 0 |
| DDX46 | -1.529325214 | 0 |
| RBM12 | -1.828208371 | 0 |
| GOLGA7 | -1.713389397 | 0 |
| RPRD1A | -1.528147012 | 0 |
| NOC3L | -1.537039951 | 0 |
| SAP30L | -1.569209090 | 0 |
| MRPS36 | -1.547982254 | 0 |
| RTFDC1 | -1.569242427 | 0 |
| PDS5A | -1.512376140 | 0 |
| PCMTD2 | -1.647562900 | 0 |
| DMXL1 | -1.656059615 | 0 |
| RAB11A | -1.509312030 | 0 |
| SPCS1 | -1.640989878 | 0 |
| IRF2BP2 | -1.758392472 | 0 |
| ATP10D | -1.815000097 | 0 |
| ALG8 | -1.543785233 | 0 |
| BLMH | -1.544001255 | 0 |
| RAP1GDS1 | -1.708593118 | 0 |
| MPV17 | -1.540687877 | 0 |
| MRPL20 | -1.695538109 | 0 |
| EIF2A | -1.515302473 | 0 |
| RPL28 | -1.537734693 | 0 |
| RRP1B | -1.758630566 | 0 |
| RABL2A | -1.542944856 | 0 |
| GCSH | -1.693462657 | 0 |
| UNG | -1.607706524 | 0 |
| PAPOLA | -1.523908510 | 0 |
| RAB7A | -1.650869331 | 0 |
| CDK19 | -1.656163009 | 0 |
| EBNA1BP2 | -1.672397583 | 0 |

| | | |
|---|---|---|
| C8orf33 | -1.547137696 | 0 |
| NOL8 | -1.504495822 | 0 |
| FAM120AOS | -1.526240391 | 0 |
| PPP1R7 | -1.647496157 | 0 |
| STX16 | -1.514449120 | 0 |
| FAM208A | -1.587357793 | 0 |
| ZFAND1 | -1.579213982 | 0 |
| HAUS6 | -1.544841277 | 0 |
| KIAA1143 | -1.548782466 | 0 |
| NSMAF | -1.535608228 | 0 |
| KLF9 | -2.476687685 | 0 |
| FAM120A | -1.556141653 | 0 |
| MSH2 | -1.695726733 | 0 |
| RPA1 | -1.994157147 | 0 |
| STAMBP | -1.579914007 | 0 |
| STX8 | -1.620698705 | 0 |
| GPR89A | -1.500856656 | 0 |
| RPF2 | -1.529037533 | 0 |
| KRIT1 | -1.600736843 | 0 |
| MPHOSPH9 | -1.691173580 | 0 |
| IGF1R | -1.751579437 | 0 |
| EBPL | -1.770185463 | 0 |
| RASA1 | -1.681420017 | 0 |
| SRSF3 | -1.619767398 | 0 |
| ACLY | -1.635410882 | 0 |
| HDDC2 | -1.907391408 | 0 |
| ABCB10 | -1.681948592 | 0 |
| LAMP1 | -1.688982279 | 0 |
| EIF3B | -1.587109620 | 0 |
| ENOPH1 | -1.658881040 | 0 |
| UCHL3 | -1.786834572 | 0 |
| CDK6 | -1.788820528 | 0 |
| ACADM | -1.933727301 | 0 |
| UBE2B | -1.526527699 | 0 |
| HDAC2 | -1.500053483 | 0 |
| PRPSAP2 | -1.690141238 | 0 |
| STRBP | -1.649195799 | 0 |
| CCDC14 | -1.788018557 | 0 |
| NDUFB5 | -1.762076876 | 0 |
| TPP1 | -1.752516346 | 0 |
| XYLT1 | -1.743191179 | 0 |
| NUCB2 | -2.090795642 | 0 |
| NXT2 | -1.703642943 | 0 |
| CCDC22 | -1.526395244 | 0 |

| | | |
|---|---|---|
| INTS6 | -1.674963431 | 0 |
| LYPLA1 | -1.768452002 | 0 |
| VPS54 | -1.551802627 | 0 |
| MBNL3 | -1.713342945 | 0 |
| MLH1 | -1.537428790 | 0 |
| MRPL37 | -1.613077440 | 0 |
| GNG10 | -1.592227643 | 0 |
| PROSC | -1.632061686 | 0 |
| MTO1 | -1.618043835 | 0 |
| SLC39A8 | -2.161725993 | 0 |
| AK6 | -1.531468360 | 0 |
| PANK2 | -1.509791315 | 0 |
| VPS13A | -1.512445828 | 0 |
| SDHB | -1.575087927 | 0 |
| TRMT10C | -1.627442138 | 0 |
| RPL22 | -1.544803190 | 0 |
| SLC30A9 | -1.522367616 | 0 |
| RNFT1 | -1.512590447 | 0 |
| RPS23 | -1.745879156 | 0 |
| PNN | -1.565061692 | 0 |
| PFDN4 | -1.617894234 | 0 |
| NPHP4 | -1.638652659 | 0 |
| BRI3BP | -1.966776343 | 0 |
| MSN | -1.627911587 | 0 |
| SETDB2 | -1.738675911 | 0 |
| CNPY2 | -1.652738233 | 0 |
| GPATCH4 | -1.516980893 | 0 |
| HPRT1 | -1.709959046 | 0 |
| MTHFD1 | -1.770518027 | 0 |
| VBP1 | -1.689049143 | 0 |
| PPIL3 | -1.669239656 | 0 |
| EID1 | -1.706334792 | 0 |
| NDRG3 | -1.525600360 | 0 |
| SPG11 | -1.813175595 | 0 |
| GIMAP2 | -2.036253546 | 0 |
| WDR41 | -1.651558454 | 0 |
| FBXO9 | -1.528120986 | 0 |
| PMS1 | -1.808949477 | 0 |
| ME3 | -1.558189353 | 0 |
| FNDC3A | -1.513150473 | 0 |
| COPS6 | -1.541061605 | 0 |
| TMEM97 | -1.547127670 | 0 |
| NUDCD2 | -1.669501103 | 0 |
| ATP6V1D | -1.642764315 | 0 |

| | | |
|---|---|---|
| NCKAP1L | -1.559898321 | 0 |
| VAMP7 | -1.893392395 | 0 |
| EEF1E1 | -1.550914562 | 0 |
| RUVBL2 | -1.517544054 | 0 |
| PPFIA1 | -1.576918871 | 0 |
| SMC6 | -1.734353947 | 0 |
| HNRNPA0 | -1.884757949 | 0 |
| ZNRF1 | -1.503946942 | 0 |
| CHD9 | -1.544979343 | 0 |
| SLC35A1 | -1.604161735 | 0 |
| CNBP | -1.691095643 | 0 |
| HADHB | -1.506651781 | 0 |
| CAT | -1.924574523 | 0 |
| BST2 | -1.707765395 | 0 |
| PQLC3 | -1.623565158 | 0 |
| BEX4 | -1.897729432 | 0 |
| CD47 | -1.706284010 | 0 |
| NEMP1 | -1.528269099 | 0 |
| ITGB3BP | -1.739202094 | 0 |
| LINC01003 | -1.762892571 | 0 |
| ARF3 | -1.635561244 | 0 |
| H2AFY | -1.927880092 | 0 |
| GALNT7 | -1.607447975 | 0 |
| TMEM167A | -1.610122581 | 0 |
| CWC27 | -1.667086378 | 0 |
| TBC1D5 | -1.543272003 | 0 |
| MCMBP | -1.776799971 | 0 |
| CCDC90B | -1.643314172 | 0 |
| VMA21 | -1.563841377 | 0 |
| HMHB1 | -1.660252568 | 0 |
| LRRC47 | -1.619842140 | 0 |
| FRMD8 | -1.626254303 | 0 |
| UBA3 | -1.642142963 | 0 |
| N4BP2 | -1.569959015 | 0 |
| YWHAB | -1.732243088 | 0 |
| TRIM13 | -1.564594214 | 0 |
| BCAS4 | -1.574060122 | 0 |
| CTBP2 | -1.700397082 | 0 |
| BDP1 | -1.586269660 | 0 |
| MMGT1 | -1.619274739 | 0 |
| FNBP1 | -2.016439720 | 0 |
| UBE4A | -1.594773295 | 0 |
| SAP18 | -1.592753969 | 0 |
| KLHDC10 | -1.621232482 | 0 |

| | | |
|---|---|---|
| ELMO1 | -1.503387695 | 0 |
| YWHAE | -1.542909890 | 0 |
| ACAA1 | -1.529879299 | 0 |
| C11orf73 | -1.648618506 | 0 |
| PHYH | -2.275591046 | 0 |
| WWP1 | -1.571446831 | 0 |
| C16orf54 | -1.677376767 | 0 |
| RPS27L | -1.875980485 | 0 |
| CCDC91 | -1.571829471 | 0 |
| SKIV2L2 | -1.527255199 | 0 |
| SULT1A1 | -1.648150280 | 0 |
| PURB | -1.581162633 | 0 |
| TMEM219 | -1.578042528 | 0 |
| AP5M1 | -1.520016960 | 0 |
| HSBP1 | -1.545918923 | 0 |
| ALG5 | -1.544222017 | 0 |
| HP1BP3 | -1.715367657 | 0 |
| CNIH1 | -1.574811200 | 0 |
| CCNT1 | -1.638443387 | 0 |
| SLC44A1 | -1.925614037 | 0 |
| MRPS30 | -1.601316798 | 0 |
| RPP40 | -1.565821693 | 0 |
| DOCK7 | -1.580568673 | 0 |
| ACER3 | -1.512308816 | 0 |
| PRPF18 | -1.510687200 | 0 |
| STX17 | -1.533677460 | 0 |
| MTDH | -1.669506452 | 0 |
| GALNT2 | -1.833067072 | 0 |
| HEBP1 | -1.669935822 | 0 |
| CREB1 | -1.575412636 | 0 |
| MED28 | -1.597558480 | 0 |
| HAT1 | -1.764380703 | 0 |
| PRPS2 | -1.774057283 | 0 |
| IQGAP1 | -1.690552685 | 0 |
| FUT4 | -1.664731465 | 0 |
| SMARCA2 | -1.644197617 | 0 |
| CHST11 | -1.607529805 | 0 |
| ATRX | -1.508151875 | 0 |
| MRI1 | -1.623314282 | 0 |
| HNRNPA3 | -1.631771650 | 0 |
| RPN2 | -1.671469114 | 0 |
| CSE1L | -1.635810854 | 0 |
| CDIPT | -1.607473980 | 0 |
| POP5 | -1.950388618 | 0 |

| | | |
|---|---|---|
| CERS6 | -1.576263698 | 0 |
| PABPC4 | -1.564610187 | 0 |
| EIF2S1 | -1.524529778 | 0 |
| ANAPC5 | -1.644134570 | 0 |
| COMT | -1.755580438 | 0 |
| FKBP3 | -1.715694327 | 0 |
| ADI1 | -1.677097022 | 0 |
| ARHGEF6 | -1.704883272 | 0 |
| NAA38 | -1.536053753 | 0 |
| FTSJ2 | -1.629868572 | 0 |
| UPF3B | -1.559236927 | 0 |
| VPS45 | -1.508193508 | 0 |
| GSTO1 | -1.621813438 | 0 |
| LINC00674 | -1.915732740 | 0 |
| CARD8 | -1.641764444 | 0 |
| SLC35A3 | -1.518026136 | 0 |
| NMI | -1.643053029 | 0 |
| GLRX3 | -1.621577627 | 0 |
| IMPDH2 | -1.618793107 | 0 |
| TPM4 | -1.596196603 | 0 |
| UROD | -1.667437691 | 0 |
| GDI2 | -1.597170643 | 0 |
| SLC7A1 | -1.620430163 | 0 |
| EIF3M | -1.671803686 | 0 |
| CCNC | -1.681659593 | 0 |
| PCNX | -1.540633179 | 0 |
| ASAP1 | -1.904186133 | 0 |
| EIF2B1 | -1.635004325 | 0 |
| BORCS7 | -1.573331859 | 0 |
| CAPRIN1 | -1.626231887 | 0 |
| DPYSL2 | -1.944105934 | 0 |
| ANKRD27 | -1.529334089 | 0 |
| RAD23B | -1.589117016 | 0 |
| FOXO3 | -1.646938098 | 0 |
| SLIRP | -1.616908061 | 0 |
| CCT7 | -1.522872402 | 0 |
| BCL11A | -1.697077445 | 0 |
| ZNF195 | -1.642672866 | 0 |
| POLR2H | -1.524468648 | 0 |
| LEPROT | -1.659248923 | 0 |
| FAF1 | -1.531006783 | 0 |
| C1orf43 | -1.572361224 | 0 |
| SCOC | -1.687821144 | 0 |
| RAB10 | -1.536557204 | 0 |

| | | |
|---|---|---|
| ARCN1 | -1.606480549 | 0 |
| CMAHP | -1.595891191 | 0 |
| MRPL36 | -1.626997306 | 0 |
| PDIA6 | -1.626600437 | 0 |
| PPP2R5C | -1.681832566 | 0 |
| OLA1 | -1.584893941 | 0 |
| FAM98B | -1.687972021 | 0 |
| TMEM173 | -1.664794469 | 0 |
| STK17B | -1.737641158 | 0 |
| EIF2AK2 | -1.824363063 | 0 |
| SPG21 | -1.572496040 | 0 |
| ZC3H15 | -1.616629497 | 0 |
| NASP | -1.566731656 | 0 |
| COQ5 | -1.510466084 | 0 |
| ANGEL2 | -1.630539306 | 0 |
| RUNX1 | -1.567624797 | 0 |
| FLNB | -1.647543400 | 0 |
| CTSS | -1.526623492 | 0 |
| EFHC1 | -1.542416693 | 0 |
| CFAP97 | -1.590457226 | 0 |
| MAEA | -1.582809877 | 0 |
| ADA | -1.623784065 | 0 |
| ZBTB44 | -1.548043095 | 0 |
| EIF2D | -1.548953411 | 0 |
| EXOC1 | -1.636257949 | 0 |
| CROCCP2 | -1.674887201 | 0 |
| MEAF6 | -1.535817108 | 0 |
| ARMC1 | -1.501361720 | 0 |
| ECI2 | -1.590463936 | 0 |
| DYNLL1 | -2.095292704 | 0 |
| CREBZF | -1.562716385 | 0 |
| ZNF32 | -1.518710335 | 0 |
| PTBP1 | -1.636172453 | 0 |
| KRCC1 | -1.861686872 | 0 |
| HENMT1 | -1.521276482 | 0 |
| AHCY | -1.564498374 | 0 |
| ALDH6A1 | -1.627901732 | 0 |
| ATRAID | -1.577392725 | 0 |
| SRSF1 | -1.578640666 | 0 |
| IRAK3 | -1.848750269 | 0 |
| ADCY9 | -1.650675972 | 0 |
| GNL3 | -1.612148294 | 0 |
| GTF3C2 | -1.507729685 | 0 |
| GPX1 | -1.614067414 | 0 |

| | | |
|---|---|---|
| C7orf55 | -1.541839458 | 0 |
| HNRNPAB | -1.690236076 | 0 |
| COMMD8 | -1.589518389 | 0 |
| ZNHIT6 | -1.554718303 | 0 |
| PRPF4 | -1.508894916 | 0 |
| MRPS12 | -1.520368183 | 0 |
| PIK3R1 | -1.701084190 | 0 |
| PSIP1 | -1.662921800 | 0 |
| RSL1D1 | -1.653972021 | 0 |
| ARID1B | -1.640520914 | 0 |
| CCDC59 | -1.538441656 | 0 |
| TMEM230 | -1.701592068 | 0 |
| MOAP1 | -1.580683543 | 0 |
| PRIM1 | -1.619422627 | 0 |
| GTF2A2 | -1.641214880 | 0 |
| SNX6 | -1.523824044 | 0 |
| PSMA4 | -1.627391891 | 0 |
| PPP2R5A | -1.546269970 | 0 |
| PTPN11 | -1.545235292 | 0 |
| ZMYND11 | -1.556975630 | 0 |
| C20orf194 | -1.518793017 | 0 |
| PPP2CB | -1.599760168 | 0 |
| RPL36A | -1.511039586 | 0 |
| MBD2 | -1.598333860 | 0 |
| BRD7 | -1.504262486 | 0 |
| DHX29 | -1.574165029 | 0 |
| ZNF83 | -1.596413358 | 0 |
| ZNF559 | -1.531606506 | 0 |
| UBE2J1 | -1.763626377 | 0 |
| NDUFB6 | -1.601668599 | 0 |
| VIMP | -1.529508314 | 0 |
| UBR7 | -1.650859458 | 0 |
| ASMTL | -1.569381855 | 0 |
| TMBIM4 | -1.585942035 | 0 |
| PDZD11 | -1.528919505 | 0 |
| KCTD18 | -1.509058898 | 0 |
| SORD | -1.759023113 | 0 |
| LSM2 | -1.503413921 | 0 |
| HACD4 | -1.764301451 | 0 |
| IER3IP1 | -1.679592483 | 0 |
| HDLBP | -1.508299947 | 0 |
| ATP6AP2 | -1.669720705 | 0 |
| ERGIC2 | -1.515102268 | 0 |
| KARS | -1.506337460 | 0 |

| | | |
|---|---|---|
| OSBPL9 | -1.541651880 | 0 |
| CCT3 | -1.652875433 | 0 |
| TMEM14C | -1.693144346 | 0 |
| ZNF45 | -1.522322250 | 0 |
| SCP2 | -1.786974999 | 0 |
| STX7 | -1.563600320 | 0 |
| APOBEC3G | -1.557455785 | 0 |
| CTBP1 | -1.633189623 | 0 |
| RLIM | -1.794757102 | 0 |
| ERGIC3 | -1.503980887 | 0 |
| FAM96A | -1.744496202 | 0 |
| SLC25A46 | -1.624328348 | 0 |
| CD164 | -1.650786878 | 0 |
| ISG20 | -1.788104767 | 0 |
| MESDC2 | -1.605403465 | 0 |
| ETV6 | -1.971248918 | 0 |
| TTC28 | -1.925616686 | 0 |
| IARS2 | -1.563713243 | 0 |
| MLLT10 | -1.534396809 | 0 |
| CIITA | -1.601454141 | 0 |
| SCAI | -1.547542568 | 0 |
| IMMT | -1.540180355 | 0 |
| TMEM70 | -1.593205354 | 0 |
| CYCS | -1.545055080 | 0 |
| TMEM14B | -1.586988900 | 0 |
| PCMT1 | -1.585187061 | 0 |
| CMPK1 | -1.531765553 | 0 |
| EIF4G3 | -1.542696847 | 0 |
| HAUS1 | -1.624565150 | 0 |
| ZNF700 | -1.529651124 | 0 |
| BECN1 | -1.568773345 | 0 |
| UBE2D2 | -1.515384912 | 0 |
| MTHFD1L | -1.609201699 | 0 |
| USP48 | -1.531401554 | 0 |
| C5orf15 | -1.528757129 | 0 |
| SSBP1 | -1.775628484 | 0 |
| MRPL51 | -1.573513877 | 0 |
| DERL1 | -1.578029769 | 0 |
| SSR1 | -1.536885626 | 0 |
| HACL1 | -1.537075054 | 0 |
| APEX1 | -1.504246458 | 0 |
| RNF130 | -1.960776547 | 0 |
| CCNG1 | -1.585480939 | 0 |
| RNASET2 | -1.593994703 | 0 |

| | | |
|---|---|---|
| GUCY1A3 | -1.837275039 | 0 |
| IFI16 | -1.715550657 | 0 |
| TECR | -1.508002770 | 0 |
| ATIC | -1.696649206 | 0 |
| TAF1D | -1.565059066 | 0 |
| TOR1AIP1 | -1.658940214 | 0 |
| PPARA | -1.508913664 | 0 |
| DDIT4 | -1.913968214 | 0 |
| EEF1A1 | -1.645107962 | 0 |
| SEPHS1 | -1.500759627 | 0 |
| C18orf32 | -1.534793136 | 0 |
| ACAT1 | -1.605982045 | 0 |
| CDC26 | -1.574964824 | 0 |
| MALT1 | -1.592416245 | 0 |
| DAD1 | -1.809703756 | 0 |
| CTSB | -1.622885615 | 0 |
| STAP1 | -2.534686265 | 0 |
| CBFB | -1.708272077 | 0 |
| CD58 | -2.125364747 | 0 |
| CCZ1 | -1.513517249 | 0 |
| MAP3K4 | -1.532597673 | 0 |
| TPD52L2 | -1.701062987 | 0 |
| UGGT1 | -1.541018875 | 0 |
| TPI1 | -1.507048447 | 0 |
| TRIM73 | -1.740648881 | 0 |
| EIF1AX | -1.561081703 | 0 |
| RCSD1 | -2.010203726 | 0 |
| NSMCE4A | -1.534638615 | 0 |
| LOC102606465 | -1.529628404 | 0 |
| MTIF3 | -1.582239455 | 0 |
| THAP9-AS1 | -1.557602559 | 0 |
| PGK1 | -1.551827210 | 0 |
| GLT8D1 | -1.599733220 | 0 |
| PRMT1 | -1.519266334 | 0 |
| NOP56 | -1.567845499 | 0 |
| NUP43 | -1.553975269 | 0 |
| UBR5-AS1 | -1.529312578 | 0 |
| RASGRP2 | -1.628335181 | 0 |
| BNIP2 | -1.644406202 | 0 |
| FAM104A | -1.545654111 | 0 |
| RAB37 | -1.682370756 | 0 |
| B3GNT2 | -1.580543890 | 0 |
| DDX60 | -1.528525165 | 0 |
| GNAI3 | -1.562194912 | 0 |

| | | |
|---|---|---|
| VPS13B | -1.668951807 | 0 |
| AFG3L2 | -1.521014652 | 0 |
| POLR3E | -1.554286374 | 0 |
| RASD1 | -2.019916871 | 0 |
| SMARCC1 | -1.522895398 | 0 |
| ADNP | -1.572187131 | 0 |
| ZEB2 | -1.829078707 | 0 |
| NUDT4 | -1.556390754 | 0 |
| CNPY3 | -1.595840475 | 0 |
| PSMA7 | -1.549260297 | 0 |
| ZNF410 | -1.520145504 | 0 |
| THOC7 | -1.600079341 | 0 |
| STK26 | -1.577930875 | 0 |
| CETN2 | -1.574414431 | 0 |
| LYST | -1.889263035 | 0 |
| CDK4 | -1.542167565 | 0 |
| HMGN4 | -1.560171676 | 0 |
| ANKRD28 | -1.711333058 | 0 |
| TAP2 | -1.585397566 | 0 |
| CFDP1 | -1.540535594 | 0 |
| LSG1 | -1.541319809 | 0 |
| PSMB8-AS1 | -1.890534367 | 0 |
| RNF125 | -1.724417832 | 0 |
| CPSF3 | -1.531330308 | 0 |
| GNA15 | -1.887466954 | 0 |
| GUSB | -1.518614327 | 0 |
| SSB | -1.519328842 | 0 |
| RBM8A | -1.517371545 | 0 |
| HACD2 | -1.520506720 | 0 |
| P2RX5 | -2.244658486 | 0 |
| GTF2H1 | -1.524735049 | 0 |
| CREG1 | -1.929765552 | 0 |
| TIGAR | -1.597184424 | 0 |
| KLF12 | -1.536785941 | 0 |
| STAT1 | -1.840272171 | 0 |
| HLA-DOA | -1.580733524 | 0 |
| FLI1 | -1.712546957 | 0 |
| FDFT1 | -1.525874031 | 0 |
| LIMS1 | -1.502261348 | 0 |
| SAP30 | -1.911431093 | 0 |
| NSUN6 | -1.506556428 | 0 |
| CKLF | -1.811882110 | 0 |
| EIF5 | -1.557485423 | 0 |
| SMIM24 | -1.681780930 | 0 |

| | | |
|---|---|---|
| ANP32A | -1.513730835 | 0 |
| TARSL2 | -1.519679589 | 0 |
| ERP44 | -1.515159063 | 0 |
| IL17RA | -1.611862198 | 0 |
| TUG1 | -1.538032683 | 0 |
| TSNAX | -1.563844078 | 0 |
| SPCS2 | -1.610444689 | 0 |
| TBCC | -1.553013427 | 0 |
| LIPA | -1.732079999 | 0 |
| SEP15 | -1.570693026 | 0 |
| NDUFA8 | -1.671055730 | 0 |
| CRYZL1 | -1.533725521 | 0 |
| SRSF7 | -1.685240715 | 0 |
| LOC285812 | -1.707213438 | 0 |
| CHSY1 | -1.665588155 | 0 |
| CYP2R1 | -1.524707984 | 0 |
| NAP1L1 | -1.627737058 | 0 |
| HNRNPH3 | -1.524451698 | 0 |
| ARL8B | -1.660954690 | 0 |
| CLIC4 | -1.662667662 | 0 |
| UBAP2 | -1.589688441 | 0 |
| LSM5 | -1.586807849 | 0 |
| MAGT1 | -1.641469629 | 0 |
| MRPS31 | -1.524980520 | 0 |
| DECR1 | -1.505363448 | 0 |
| PIGY | -1.530767102 | 0 |
| ZNF655 | -1.529575251 | 0 |
| HCST | -1.508821694 | 0 |
| PIKFYVE | -1.573443102 | 0 |
| MYLIP | -1.789888025 | 0 |
| BOLA3 | -1.575719867 | 0 |
| NDUFAB1 | -1.514929623 | 0 |
| PTRHD1 | -1.522179252 | 0 |
| LRRC59 | -1.558392736 | 0 |
| TAF13 | -1.864038097 | 0 |
| HLTF | -1.583955133 | 0 |
| CLPTM1L | -1.707327733 | 0 |
| ERMP1 | -1.508006804 | 0 |
| CCNH | -1.535030795 | 0 |
| CYB5A | -1.541954907 | 0 |
| VRK1 | -1.552029469 | 0 |
| MYC | -1.669760413 | 0 |
| DAAM1 | -1.605688548 | 0 |
| AMZ2 | -1.545744673 | 0 |

| | | |
|---|---|---|
| PSMD14 | -1.561213476 | 0 |
| JMJD6 | -1.647027476 | 0 |
| RAN | -1.702345862 | 0 |
| RRM1 | -1.620527745 | 0 |
| GNPAT | -1.523150934 | 0 |
| RB1 | -1.751098041 | 0 |
| OTUB1 | -1.509159875 | 0 |
| LSM4 | -1.541887449 | 0 |
| GALC | -1.608378603 | 0 |
| SIK1 | -2.012187561 | 0 |
| KIAA0368 | -1.614631993 | 0 |
| NDUFA5 | -1.539753631 | 0 |
| FAM96B | -1.521791169 | 0 |
| FAM101B | -2.687098371 | 0 |
| TOMM5 | -1.613076971 | 0 |
| SKAP2 | -1.731523443 | 0 |
| LOC100130458 | -1.628724209 | 0 |
| TUBGCP3 | -1.516823325 | 0 |
| SH3GLB1 | -1.541868447 | 0 |
| PDE7A | -1.602561772 | 0 |
| ETFA | -1.670914894 | 0 |
| PHF20 | -1.577487526 | 0 |
| FANCI | -1.515563635 | 0 |
| DHFR | -1.618410797 | 0 |
| CD46 | -1.522518208 | 0 |
| GGCX | -1.535781850 | 0 |
| MCM4 | -1.570811885 | 0 |
| ERLIN1 | -1.513458855 | 0 |
| SUPT20H | -1.504029768 | 0 |
| PRDX1 | -1.731800957 | 0 |
| SH3BGRL | -1.587333876 | 0 |
| LOC105369243 | -1.566875870 | 0 |
| ANXA4 | -1.689945718 | 0 |
| COX11 | -1.599381149 | 0 |
| CLTA | -1.541810331 | 0 |
| NDE1 | -1.553173551 | 0 |
| LOC374443 | -1.654606110 | 0 |
| LINC00938 | -1.523415373 | 0 |
| HSPB11 | -1.605678412 | 0 |
| CHTOP | -1.549384012 | 0 |
| RAP1GAP2 | -1.598205223 | 0 |
| RCBTB2 | -1.860112918 | 0 |
| ERAP1 | -1.565343348 | 0 |
| MAPK1IP1L | -1.544470473 | 0 |

| | | |
|---|---|---|
| ADGRE2 | -1.742996888 | 0 |
| FAM213A | -1.684069556 | 0 |
| HDHD2 | -1.895361715 | 0 |
| TPD52 | -1.583824225 | 0 |
| ACTR3 | -1.534164887 | 0 |
| NUDT21 | -1.545052341 | 0 |
| PIGP | -1.556510591 | 0 |
| CSDE1 | -1.563540593 | 0 |
| KLRK1 | -1.836271964 | 0 |
| AAED1 | -1.516882279 | 0 |
| FOXN3 | -1.580078745 | 0 |
| DPM1 | -1.521874914 | 0 |
| BMP2K | -1.586775381 | 0 |
| CD44 | -2.127613070 | 0 |
| PPT1 | -1.699312794 | 0 |
| PSMB10 | -1.599259939 | 0 |
| CALM1 | -1.607722373 | 0.022862369 |
| ARHGAP15 | -1.503066854 | 0.022862369 |
| RPS6KA3 | -1.615140132 | 0.022862369 |
| UTP4 | -1.509372194 | 0.022862369 |
| ADCY7 | -1.575970424 | 0.022862369 |
| RNF135 | -1.664934516 | 0.022862369 |
| RAB34 | -1.543227785 | 0.022862369 |
| COPB2 | -1.547852407 | 0.022862369 |
| RILPL2 | -1.670584040 | 0.022862369 |
| MCL1 | -1.621050934 | 0.022862369 |
| ANKRD49 | -1.503288142 | 0.022862369 |
| SERPINB1 | -1.673874698 | 0.022862369 |
| IFT57 | -1.572404996 | 0.022862369 |
| ACTR10 | -1.505537371 | 0.022862369 |
| VMP1 | -1.587724340 | 0.022862369 |
| PLSCR1 | -1.667007522 | 0.022862369 |
| MTHFD2 | -1.656708290 | 0.022862369 |
| RFC4 | -1.679871245 | 0.022862369 |
| ANAPC13 | -1.505621585 | 0.022862369 |
| ADPRM | -1.520237164 | 0.022862369 |
| DYNLT1 | -1.563292567 | 0.022862369 |
| ZBTB11 | -1.587277700 | 0.022862369 |
| TMEM258 | -1.524627154 | 0.022862369 |
| NCF4 | -1.585238085 | 0.022862369 |
| LNPEP | -1.567083368 | 0.022862369 |
| SLC27A3 | -1.614206928 | 0.022862369 |
| PTTG1IP | -1.603294986 | 0.022862369 |
| CDKN1B | -1.668109497 | 0.022862369 |

| | | |
|---|---|---|
| CSRP2 | -1.717138633 | 0.022862369 |
| MDH1 | -1.542549706 | 0.022862369 |
| CEP152 | -1.523266543 | 0.022862369 |
| LYRM4 | -1.518755917 | 0.022862369 |
| PRPF4B | -1.571704936 | 0.022862369 |
| SLC9A7 | -1.581188972 | 0.022862369 |
| EVI2B | -1.772670273 | 0.022862369 |
| ATP11A | -1.568414163 | 0.022862369 |
| LILRA2 | -1.698815803 | 0.022862369 |
| TXN | -1.587851345 | 0.022862369 |
| SNHG12 | -1.508138175 | 0.022862369 |
| RGS19 | -1.506867052 | 0.022862369 |
| TTC14 | -1.630355484 | 0.022862369 |
| AK3 | -1.509259942 | 0.022862369 |
| TRAPPC10 | -1.521668613 | 0.022862369 |
| ZNF331 | -1.661185409 | 0.022862369 |
| GTF3C6 | -1.522857018 | 0.022862369 |
| VAV3 | -1.582082137 | 0.022862369 |
| MRPL9 | -1.614950541 | 0.022862369 |
| CCDC85B | -1.507500562 | 0.022862369 |
| BTN3A2 | -1.692803779 | 0.022862369 |
| SLU7 | -1.572445128 | 0.022862369 |
| ALG2 | -1.505270900 | 0.022862369 |
| ZMAT1 | -1.587803728 | 0.022862369 |
| HIST1H2AC | -1.873331462 | 0.022862369 |
| TRAM1 | -1.502838493 | 0.022862369 |
| RNF138 | -1.595696871 | 0.022862369 |
| MCM6 | -1.718241053 | 0.022862369 |
| SNW1 | -1.515768992 | 0.022862369 |
| BCAT1 | -1.897397264 | 0.022862369 |
| SMAP1 | -1.572618054 | 0.022862369 |
| COA3 | -1.530064635 | 0.022862369 |
| MDH2 | -1.505490708 | 0.022862369 |
| TDP2 | -1.508240856 | 0.022862369 |
| NDUFAF3 | -1.527118377 | 0.022862369 |
| IRF7 | -1.568893178 | 0.022862369 |
| DBI | -1.588999524 | 0.022862369 |
| CUX1 | -1.546154881 | 0.022862369 |
| RABEP2 | -1.531510196 | 0.022862369 |
| AGAP1 | -2.177605811 | 0.022862369 |
| PABPC1L | -1.574640734 | 0.022862369 |
| ST3GAL1 | -1.535769640 | 0.022862369 |
| RNF6 | -1.578851866 | 0.022862369 |
| TTC3 | -1.536040039 | 0.022862369 |

| | | |
|---|---|---|
| MBNL1 | -1.559718550 | 0.022862369 |
| SRGN | -2.125509367 | 0.022862369 |
| USP1 | -1.585615673 | 0.022862369 |
| HLA-DPA1 | -1.851583014 | 0.022862369 |
| NREP | -1.865779569 | 0.022862369 |
| TNFSF13B | -1.669473309 | 0.022862369 |
| GNB4 | -1.572245270 | 0.022862369 |
| KLHL6 | -1.575911060 | 0.022862369 |
| PYCARD | -1.714727660 | 0.022862369 |
| PRKRA | -1.575945918 | 0.022862369 |
| SUN1 | -1.516403061 | 0.022862369 |
| PROSER1 | -1.523519857 | 0.022862369 |
| HSP90B1 | -1.570114820 | 0.022862369 |
| IL13RA1 | -1.529881248 | 0.042478845 |
| PSD3 | -1.621564604 | 0.042478845 |
| PKIG | -1.783039975 | 0.042478845 |
| HLA-DQA1 | -2.147668202 | 0.042478845 |
| CCNJ | -1.696525938 | 0.042478845 |
| RAC2 | -1.672680255 | 0.042478845 |
| HSH2D | -1.513629181 | 0.042478845 |
| ZNF394 | -1.593414236 | 0.042478845 |
| U2SURP | -1.554984308 | 0.042478845 |
| COMMD7 | -1.558646725 | 0.042478845 |
| PCNXL2 | -1.531860432 | 0.042478845 |
| FOSL2 | -1.982583355 | 0.042478845 |
| UBE2Q2 | -1.648656448 | 0.042478845 |
| UTRN | -1.523317415 | 0.042478845 |
| ACTN1 | -1.826296296 | 0.042478845 |
| CTNNA1 | -1.528783654 | 0.042478845 |
| PAM | -1.795713601 | 0.042478845 |
| TNFSF4 | -1.619183499 | 0.042478845 |
| PRNP | -1.623149288 | 0.042478845 |
| AZIN1 | -1.568706307 | 0.042478845 |
| CD72 | -2.019541975 | 0.042478845 |
| SGK223 | -1.507872982 | 0.042478845 |
| CD24 | -1.821030386 | 0.042478845 |
| ZBTB20 | -1.540892084 | 0.042478845 |
| UGP2 | -1.630130071 | 0.042478845 |
| USP36 | -1.713115528 | 0.042478845 |
| C18orf8 | -1.537811543 | 0.042478845 |
| MCM7 | -1.525092494 | 0.042478845 |
| SH2B3 | -1.826198826 | 0.042478845 |
| HERC5 | -1.566068954 | 0.042478845 |
| BZRAP1-AS1 | -1.548758608 | 0.042478845 |

| | | |
|---|---|---|
| IL2RG | -1.509844224 | 0.042478845 |
| CLEC14A | -1.585782913 | 0.042478845 |
| TIMM17A | -1.524767845 | 0.042478845 |
| RCAN3 | -1.523363557 | 0.042478845 |
| TMED5 | -1.502972459 | 0.042478845 |
| RNF11 | -1.542243568 | 0.042478845 |
| SMIM19 | -1.645495163 | 0.042478845 |
| PCDH9 | -1.856448432 | 0.042478845 |
| GALNT3 | -1.568684898 | 0.042478845 |
| TXLNGY | -1.521596712 | 0.042478845 |
| YBX3 | -1.717356717 | 0.042478845 |
| TRIM14 | -1.537832058 | 0.042478845 |
| PARP8 | -1.559942285 | 0.042478845 |
| PXK | -1.505573152 | 0.042478845 |
| LINC00623 | -1.550938314 | 0.042478845 |
| MED17 | -1.540529752 | 0.042478845 |
| PRKACB | -1.707473660 | 0.042478845 |
| TIMM23 | -1.550379052 | 0.042478845 |
| RFC5 | -1.588664769 | 0.042478845 |
| FAM69C | -1.545500243 | 0.042478845 |
| INAFM2 | -1.890672164 | 0.042478845 |
| ZHX2 | -1.533453107 | 0.042478845 |
| EAF2 | -1.712618714 | 0.042478845 |
| ICAM2 | -1.613897170 | 0.042478845 |
| PTPRM | -2.019355039 | 0.042478845 |
| PIK3AP1 | -1.511818427 | 0.042478845 |
| MGME1 | -1.561576416 | 0.042478845 |
| NRIP1 | -1.728645822 | 0.042478845 |
| MARCH6 | -1.537808458 | 0.120202147 |
| CDCA7 | -1.729942188 | 0.120202147 |
| OAT | -1.582227633 | 0.120202147 |
| HBEGF | -1.739609792 | 0.120202147 |
| MRC1 | -1.587383812 | 0.120202147 |
| PSMB9 | -1.610747674 | 0.120202147 |
| PPIB | -1.504839153 | 0.120202147 |
| DOK3 | -1.575418335 | 0.120202147 |
| PNP | -1.643111011 | 0.120202147 |
| GSN | -1.609439838 | 0.120202147 |
| SEC16A | -1.505967212 | 0.120202147 |
| RAC1 | -1.550490154 | 0.120202147 |
| NKG7 | -1.632224192 | 0.120202147 |
| DR1 | -1.502840433 | 0.120202147 |
| GMNN | -1.577369891 | 0.120202147 |
| XBP1 | -1.524552279 | 0.120202147 |

| | | |
|---|---|---|
| CXCR4 | -1.642367330 | 0.120202147 |
| CMPK2 | -1.591895348 | 0.120202147 |
| ZNF664 | -1.567831354 | 0.120202147 |
| CYTIP | -1.924821796 | 0.120202147 |
| ARFGAP3 | -1.539673988 | 0.120202147 |
| AFF3 | -1.813320576 | 0.120202147 |
| C4orf3 | -1.661922118 | 0.120202147 |
| GFOD1 | -1.500557131 | 0.120202147 |
| SEC14L1 | -1.645807143 | 0.120202147 |
| SLBP | -1.575127583 | 0.120202147 |
| SOX7 | -1.665151356 | 0.120202147 |
| NDUFA12 | -1.584707708 | 0.120202147 |
| CEBPG | -1.563624363 | 0.120202147 |
| PMS2P5 | -1.686692572 | 0.120202147 |
| RBBP6 | -1.560742863 | 0.120202147 |
| SIPA1L2 | -2.141979041 | 0.120202147 |
| CBX4 | -1.513465744 | 0.120202147 |
| BICD2 | -1.556610046 | 0.120202147 |
| FTH1 | -1.733514991 | 0.120202147 |
| VNN1 | -1.538516045 | 0.120202147 |
| MOB3A | -1.743966712 | 0.120202147 |
| SERINC1 | -1.513690775 | 0.120202147 |
| GLIPR1 | -1.538396507 | 0.120202147 |
| SOCS2 | -2.257776230 | 0.120202147 |
| PIEZO1 | -1.500359038 | 0.120202147 |
| HPS4 | -1.598737519 | 0.120202147 |
| CDCA7L | -1.500761976 | 0.120202147 |
| NFE2L2 | -1.507925926 | 0.120202147 |
| MALAT1 | -1.729022329 | 0.120202147 |
| TM9SF3 | -1.521545484 | 0.120202147 |
| TMEM181 | -1.551140355 | 0.120202147 |
| ACSL1 | -1.934649426 | 0.120202147 |
| DUSP10 | -1.538846074 | 0.120202147 |
| TRAF3IP3 | -1.545300935 | 0.173850264 |
| RCOR1 | -1.511346072 | 0.173850264 |
| LILRB1 | -1.560033817 | 0.173850264 |
| IGFBP7 | -1.944476578 | 0.173850264 |
| TXLNG | -1.523275715 | 0.173850264 |
| ISG15 | -1.537560612 | 0.173850264 |
| MATR3 | -1.514264357 | 0.173850264 |
| CHD2 | -1.536281283 | 0.173850264 |
| SDC2 | -1.650075190 | 0.173850264 |
| CCNA1 | -1.585961397 | 0.173850264 |
| EIF1AY | -1.639911750 | 0.173850264 |

| | | |
|---|---|---|
| MACROD2 | -1.718367878 | 0.173850264 |
| TSC22D3 | -1.501833711 | 0.173850264 |
| FYTTD1 | -1.533462041 | 0.173850264 |
| GAB1 | -1.643352097 | 0.173850264 |
| FHL1 | -1.604107779 | 0.173850264 |
| TCEAL4 | -1.558186860 | 0.173850264 |
| EMB | -1.595222307 | 0.173850264 |
| STOM | -1.549829679 | 0.173850264 |
| LINC01215 | -1.557849610 | 0.173850264 |
| SOCS2-AS1 | -1.515658934 | 0.173850264 |
| TSC22D1 | -1.619078231 | 0.173850264 |
| C5orf56 | -1.512525132 | 0.173850264 |
| ITGB2 | -1.630021862 | 0.173850264 |
| IFI44 | -1.843214847 | 0.173850264 |
| CENPU | -1.529623125 | 0.173850264 |
| SFT2D1 | -1.533485559 | 0.173850264 |
| C21orf91 | -1.505455727 | 0.173850264 |
| METTL3 | -1.574637585 | 0.173850264 |
| PLAC8 | -1.649033463 | 0.173850264 |
| AKAP2 | -1.636764987 | 0.173850264 |
| HOXA9 | -1.723326274 | 0.173850264 |
| FOS | -1.589397339 | 0.173850264 |
| SDE2 | -1.512657754 | 0.173850264 |
| KCNK12 | -1.609720761 | 0.173850264 |
| ERGIC1 | -1.513767041 | 0.244260609 |
| ZNF91 | -1.504403338 | 0.244260609 |
| IFI27 | -1.633103112 | 0.244260609 |
| NR4A3 | -1.867900437 | 0.244260609 |
| UBE2G1 | -1.512265610 | 0.244260609 |
| STAG3 | -1.694531720 | 0.244260609 |
| AGPS | -1.601198880 | 0.244260609 |
| RPL18A | -1.572976960 | 0.244260609 |
| RASGRP1 | -1.573469543 | 0.244260609 |
| PRKCB | -1.561387145 | 0.244260609 |
| ABHD17C | -1.584712311 | 0.244260609 |
| PPP1R2 | -1.524252815 | 0.244260609 |
| EIF5A | -1.540240902 | 0.244260609 |
| CDH11 | -1.547121660 | 0.244260609 |
| HLA-DQB1 | -1.630832820 | 0.244260609 |
| NFIL3 | -1.792512080 | 0.244260609 |
| TIMP2 | -1.635087672 | 0.244260609 |
| NR4A2 | -1.731147668 | 0.303598806 |
| HS3ST3B1 | -1.574444973 | 0.303598806 |
| VAT1L | -1.618114684 | 0.303598806 |

| | | |
|---|---|---|
| ARRDC4 | -1.622266868 | 0.303598806 |
| MX1 | -1.665993707 | 0.303598806 |
| NSMCE1 | -1.597606626 | 0.303598806 |
| WT1 | -1.511797886 | 0.303598806 |
| DDIT4L | -1.728120032 | 0.303598806 |
| SPON2 | -1.509658385 | 0.303598806 |
| TMEM107 | -1.520923605 | 0.303598806 |
| P2RY14 | -1.584747189 | 0.303598806 |
| RUNX2 | -1.761844058 | 0.303598806 |
| GPM6B | -1.662246842 | 0.303598806 |
| LOC728175 | -1.535659122 | 0.303598806 |
| SMIM3 | -1.543417948 | 0.303598806 |
| FRMD4B | -1.503164797 | 0.303598806 |
| CLEC11A | -1.563397704 | 0.396061751 |
| VPREB1 | -1.704356153 | 0.396061751 |
| ID2 | -1.655371397 | 0.396061751 |
| KDM5D | -1.654383400 | 0.396061751 |
| IFI44L | -1.862688731 | 0.396061751 |
| THEMIS2 | -1.510670136 | 0.396061751 |
| CD34 | -1.553206666 | 0.396061751 |
| HDGFRP3 | -1.517064413 | 0.396061751 |
| RCAN1 | -1.607797224 | 0.396061751 |
| TERF2 | -1.548317445 | 0.396061751 |
| GNG11 | -1.570283258 | 0.396061751 |
| IFIT3 | -1.546882271 | 0.396061751 |
| MYO5C | -1.520283625 | 0.396061751 |
| CHST2 | -1.625439387 | 0.396061751 |
| SLC2A5 | -1.682240116 | 0.428147117 |
| PCDH17 | -1.531244382 | 0.428147117 |
| AREG | -1.634399482 | 0.428147117 |
| BANK1 | -1.601423078 | 0.428147117 |
| DDX3Y | -2.400746386 | 0.428147117 |
| ERG | -1.534940140 | 0.428147117 |
| LATS2 | -1.529710795 | 0.428147117 |
| PROM1 | -1.843319255 | 0.452851896 |
| CYTL1 | -1.684853433 | 0.452851896 |
| ITGA6 | -1.588916791 | 0.452851896 |
| DNTT | -1.589878943 | 0.477641309 |
| TCFL5 | -1.545876705 | 0.477641309 |
| RGS1 | -1.573761618 | 0.520863121 |
| TCL1A | -1.502766816 | 0.520863121 |
| SMAD1 | -1.538122376 | 0.520863121 |
| RPS4Y1 | -1.899305904 | 0.520863121 |
| DSTN | -1.518244423 | 0.554043571 |

| | | |
|---|---|---|
| PRDX2 | -1.501452892 | 0.554043571 |
| EGR1 | -1.513122622 | 0.562119550 |
| LGALS1 | -1.519237299 | 0.562119550 |

# Uncropped western blots for figures 2d-j

2d

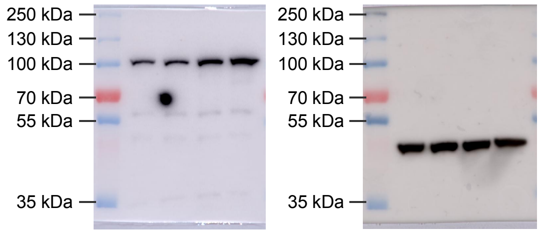


ß-Catenin ß-Actin

8% SDS PAGE (home-made).
Tris-Glycine-SDS running buffer.

Ladder: PageRuler (ThermoFisher cat# 26619).

The blot was probed with anti-ß-Catenin antibody.

After stripping, blot was probed with anti-ß-Actin antibody.

2e

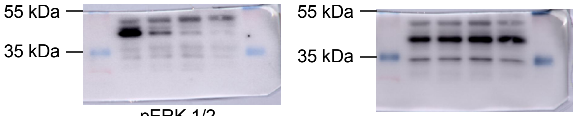


pERK 1/2 ERK2

8% SDS PAGE (home-made).
Tris-Glycine-SDS running buffer.
Ladder: PageRuler (ThermoFisher cat# 26619).
Blot was cut at 55 kDa and lower part was used.
Blot was first probed with anti-phospho-ERK 1/2 antibody.
After stripping, blot was probed with anti-ERK2 antibody.

2f

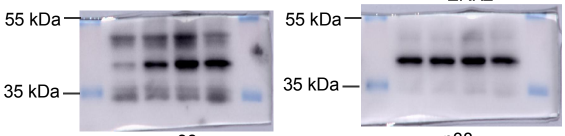


p-p38 p38

8% SDS PAGE (home-made).
Tris-Glycine-SDS running buffer.
Ladder: PageRuler (ThermoFisher cat# 26619).
Blot was cut at 55 kDa and lower part was used.
Blot was probed with anti-phospho p38 antibody.
After stripping, the blot was probed with anti-p38 antibody.

2g

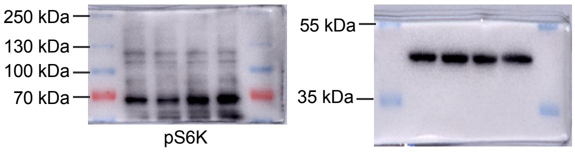


pS6K ß-Actin

8% SDS PAGE (home-made).
Tris-Glycine-SDS running buffer.
Ladder: PageRuler (ThermoFisher cat# 26619).
Blot was cut at 55 kDa.
Upper part of the blot was probed with anti-phospho S6K antibody.
Lower part of the blot was probed with anti-ß-Actin antibody.

2h

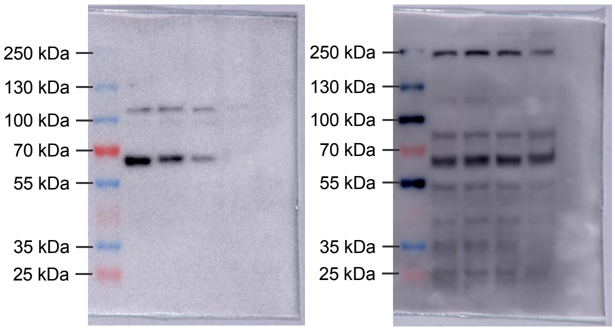


pAKT AKT

8% mPAGE Bis-Tris (Millipore).
MES-SDS running buffer.

Ladder: PageRuler (ThermoFisher cat# 26619).

The blot was probed with anti-phospho-AKT antibody.

After stripping, blot was probed with anti-AKT antibody.

2i

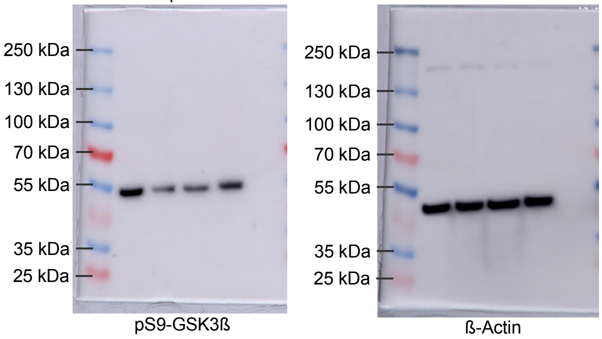


pS9-GSK3ß ß-Actin

8% mPAGE Bis-Tris (Millipore).
MES-SDS running buffer.

Ladder: PageRuler (ThermoFisher cat# 26619).

The blot was probed with anti-phospho-GSK3ß antibody.

After stripping, blot was probed with anti-ß-Actin antibody.

2j

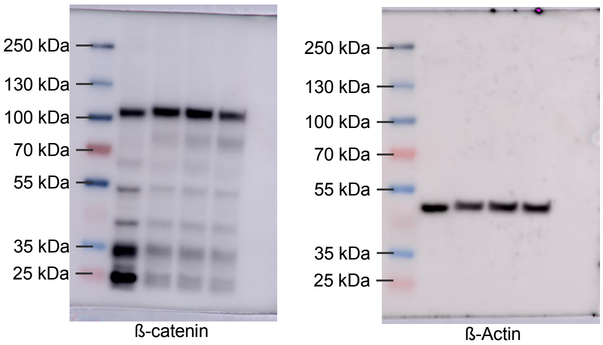


ß-catenin ß-Actin

8% mPAGE Bis-Tris (Millipore).
MES-SDS running buffer.

Ladder: PageRuler (ThermoFisher cat# 26619).

The blot was probed with anti-ß-catenin antibody.

After stripping, blot was probed with anti-ß-Actin antibody.

# Uncropped western blots for figures 5e-f

**5e**

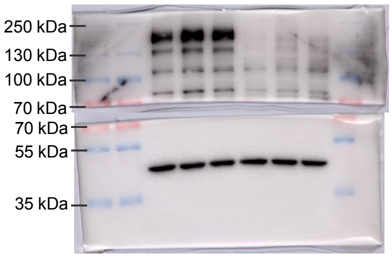


Blot was cut at 70 kDa (8% SDS PAGE).

Upper part was probed with anti-FLT3 antibody.

Lower part was probed with anti-beta-Actin antibody.

**5f**

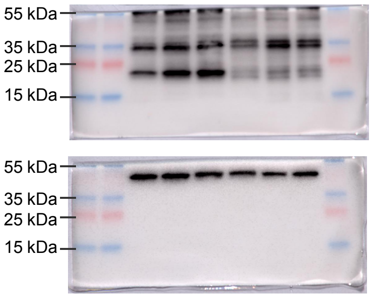


Blot was cut at 55 kDa and the lower part was used (4-15% SDS PAGE).

Blot was first probed with anti-SOCS2 antibody.

After stripping,
the blot was probed with anti-beta-Actin antibody.
